# Proceedings of the 14^th^ Annual Conference on the Science of Dissemination and Implementation in Health: Virtual. 14-16 December 2021

**DOI:** 10.1186/s13012-022-01210-x

**Published:** 2022-06-03

## I1 Introduction

### Gila Neta^1^, David Chambers^1^, Lisa Simpson^2^

#### ^1^National Cancer Institute, Rockville, MD, USA; ^2^ AcademyHealth, Washington, DC, USA

The past few years have shown the dissemination and implementation science community the importance of integrating an equity perspective and addressing social determinants of health, as well as the blurring of health and healthcare and the influences and impacts of other sectors of society. In response, dissemination and implementation science has increasingly recognized the importance of multisectoral collaborations. The resulting theme of the 14^th^ Annual Conference on the Science of Dissemination and Implementation in Health was Broadening Horizons for Impact: Incorporating Multisectoral Approaches into D&I Science. The National Institutes of Health and AcademyHealth again co-hosted the conference in collaboration with our co-sponsors the Agency for Healthcare Research and Quality (AHRQ), the Patient Centered Outcomes Research Institute (PCORI), the Robert Wood Johnson Foundation (RWJF), and the US Department of Veterans Affairs (VA). Over fourteen hundred people joined the online conference December 14-16, 2021, including 115 trainees, 15 patient scholarship recipients, and 48 participants from 20 low- and middle-income countries, including in sub-Saharan Africa, Latin America, South and Southeast Asia, Eastern Europe, and the Middle East.

As part of our continuing commitment to assess and enhance the diversity, equity, inclusion, and accessibility of the D&I Science community, for the first time all abstract submitters were asked to voluntarily provide demographic information. A review of these data showed that over all the lead presenters of accepted individual papers were 79% women, 16.6% men, and 0.2% non-binary/gender conforming, with 4.2% choosing not to disclose. Comparison of the racial/ethnic distribution among submitted vs accepted abstracts showed no systematic differences; however, from those who provided data on race or ethnicity, the total number of abstracts submitted from underrepresented minorities (URM) made up less than 12% of all individual papers including 5.8% Black or African American, 5.4% Latino/Hispanic, and 0.6% American Indian or Alaska Native.

The conference opened with keynote presenter Marcella Nunez Smith discussing the centrality of health equity to unlocking the promise of D&I science and the importance of a broad multisectoral approach to maximize population health. Building on the experience and expertise from academic and Federal leadership of equity initiatives, including those in response to the COVID pandemic, Nunez Smith discussed opportunities to focus more comprehensively on equity as a central goal of improving health and healthcare. Her comments were followed by a facilitated panel discussion with PCORI Executive Director Nakela Cook and NIMHD Director Eliseo Pérez-Stable on how equity and D&I science can be advanced conceptually and empirically through targeted research initiatives as well as systematic changes to soliciting and funding research.

Building on the keynote discussions, two subsequent plenary panels addressed critical themes of building trust for and organization of effective multisectoral partnerships. The first of these featured leading experts in cross-sectoral collaborations, Monica Peek and Stacy Lindau, both researchers based in academic institutions, joined by Nia Abdullah from MAPSCorp and Kathleen Noonan from Camden Coalition. The discussion focused on ways in which trust influences implementation, including trust in evidence and trust in the effort to implement interventions, as well as how to build and measure trust as a key outcome. The second plenary then focused on a specific case study of multisectoral approaches for transitioning military service members, which included education and employment as part of a public health approach to suicide prevention. The final keynote with Drs. Herminia Palacio and discussant Leopoldo Cabassa drew on Dr. Palacio’s wealth of experience and expertise in leading responses to the major public health and healthcare challenges over multiple decades, to inform the next generation of D&I studies.

In addition to these plenary sessions, the conference included concurrent podium and poster sessions, workshops and discussion forums, and multiple networking events. The call for abstracts generated 669 submissions, including individual paper presentations, individual posters, and panel presentations spread across nine thematic tracks: Behavioral Health, Clinical Care Settings (separated into two tracks: Patient-Level Interventions and System-Level Interventions), Global Dissemination and Implementation Science, Promoting Health Equity and Eliminating Disparities, Health Policy Dissemination and Implementation Science, Prevention and Public Health, and Models, Measures and Methods, and Building the Future of D & I Science: Training, Infrastructure, and Emerging Research Areas. This supplement is organized by those tracks and includes 113 abstracts from the concurrent paper and panel sessions, which represents a variety of dissemination and implementation research funded by our conference sponsors as well as other agencies, organizations, and systems. The additional 427 abstracts from the poster sessions are not included here but can be viewed at https://biomedcentral.spi-global.com/authorproofs/bmcproofs/index.php?id=yYzPofvuAO05132022122711lqtKrxZnjn.

Through the virtual platform, attendees were able to engage in conversations through the chat function during the plenary and concurrent sessions, allowing for participants to drive the interaction with presenters and create a valuable repository of relevant references and web-based resources which both speakers and participants shared during each of the sessions. The conference also featured virtual yoga, a social musical gathering, and daily morning coffee chats with D&I experts facilitating open discussions about key priorities for the field. These networking sessions again were hugely popular and well attended, providing attendees with the opportunity to connect with the leaders in the field. Another tremendous value of the virtual conference was the ability for us to host a significant number of participants from LMICs.

After two years of virtual conferences, we look forward to welcoming attendees back to Washington, DC, for the next D&I Science conference this December.

## Behavioral Health

### S1 Associations between system, organizational, and individual characteristics with sustained EBP fidelity across mental health systems

#### Shannon Wiltsey Stirman^1,2^, Erin Finley^3^, Jiyoung Song^4^, Tasoula Masina^5^, Syed Aajmain^2^, Jansey Lagdamen^6^, Taylor Loskot^6^, Kera Swanson^6^, Heidi LaBash^7^, Jeanine Lane^5^, Iris Sijercic^5^, Rachel Liebman^5^, Alayna Park^8^, Norman Shields^9^, Candice Monson^5^

##### ^1^National Center for PTSD, Veterans Health Administration, Menlo Park, CA, USA; ^2^Stanford University, Stanford, CA, USA; ^3^UT Health San Antonio, San Antonio, TX, USA; ^4^University of California, Berkeley, Berkeley, CA, USA; ^5^Ryerson University, Toronto, ON, Canada; ^6^National Center for PTSD, Menlo Park, CA, USA; ^7^National Center for PTSD, Palo Alto, CA, USA; ^8^Palo Alto University, Palo Alto, CA, USA; ^9^Royal Canadian Mounted Police, Westmount, QC, Canada

###### **Correspondence:** Shannon Wiltsey Stirman (sws1@stanford.edu)

**Background:**

Cognitive Processing Therapy (CPT) has been implemented in multiple mental health care systems in North America. While CPT training is standardized, there is variation in policies, support, populations served and therapist characteristics. This study examined predictors of CPT fidelity in clinics that had implemented CPT between one and ten years earlier, as part of baseline data collection for a randomized controlled trial of implementation strategies to support sustained CPT delivery in state and national healthcare systems.

**Methods:**

This study was conducted in 34 clinics across public and private healthcare systems. We examined system- and clinic-levels of fidelity (comprising adherence to the CPT protocol and skill/competence of treatment delivery) at baseline, using clinic data and observation of randomly selected CPT sessions that were recorded by therapists (*n*=137).

**Findings:**

Preliminary analyses indicated that there were no significant differences in adherence or competence across systems, *F*(5, 43) = 1.40, *p* = .244. However, at the therapist level, younger age, *b* = -0.04, *t*(36) = -2.36, *p* = .024, working at a PTSD specialty clinic, *b* = 0.65, *t*(36) = 2.22, *p* = .032, and working primarily with military or veteran patients, *b* = 0.66, *t*(36) = 2.52, *p* = .016, significantly predicted higher competence. Greater implementation leadership (ILS; Aarons, Ehrhart, & Farahnak, 2014), *b* = 0.30, *t*(33) = 2.42, *p* = .02, and Learning Organization Survey Building Block III (Leadership that reinforces learning; Garvin et al., 2008), *b* = 0.01, *t*(36) = 2.01, *p* = .050, were also significantly associated with higher competence. In addition, greater time passed since the last CPT training, *b* = -0.002, *t*(43) = -2.40, *p* = .021, predicted lower adherence.

**Implications for D&I Research:**

Few studies have investigated sustainment of EBPs across multiple systems. While fidelity did not appear to vary across systems, our findings regarding leadership and time since training suggest that fidelity may be improved by strong implementation leadership and support for therapists. These data are of critical importance for systems and organizations in understanding what contributes to sustained fidelity after initial implementation.

**Primary Funding Source:** National Institutes of Health

### S2 Trauma–focused cognitive behavioral therapy outcomes of a community based learning collaborative (CBLC): Benchmarking treatment effectiveness during a statewide implementation initiative

#### Hannah Espeleta^1^, Samuel Peer^2^, Rochelle Hanson^3^

##### ^1^Medical University of SC, Charleston, SC, USA; ^2^Stop 8112, Idaho State University, Pocatello, ID, USA; ^3^Medical University of South Carolina, Charleston, SC, USA

###### **Correspondence:** Hannah Espeleta (espeleta@musc.edu)

**Background:**

Research has underscored the importance of evaluating clinical training models used to improve community access to and implementation of evidence-based treatments (EBTs). Community-Based Learning Collaboratives (CBLCs) represent one promising training/implementation package developed to provide systematic, multidisciplinary training and support for the sustained adoption of EBTs, such as Trauma-Focused Cognitive Behavioral Therapy (TF-CBT), among community agencies. Although growing evidence suggests that CBLCs can significantly improve community-, organization-, and clinician-level factors related to EBT–and specifically TF-CBT–implementation, limited research has evaluated the impact of CBLCs on the children and families they intend to serve.

**Methods:**

The present study examined treatment outcomes for 542 youth receiving TF-CBT from community therapists enrolled in a CBLC for TF-CBT. Pre- and post-treatment, youth and caregivers completed measures of post-traumatic stress (PTS; Child PTSD Symptom Scale) and depression (Short Mood and Feelings Questionnaire–Short Version).

**Findings:**

Based on youth- and caregiver-responses, youth receiving TF-CBT from CBLC-trained therapists had large, significant pre- to post-treatment decreases in PTS (*d*s = 1.01–1.26, *p*s < .001) and depressive symptoms (*d* = 0.97, *p* < .001). Additionally, McNemar’s tests revealed a significant pre- to post-treatment decrease in the number of children who met clinical criteria for PTS, depression, and comorbid PTS and depression (*ps* < .001), with 51%–66% of youth demonstrating a clinically significant treatment response. Furthermore, benchmarking analyses indicated that the above symptom reductions were statistically equivalent to Lenz and Hollenbaugh’s (2015) meta-analytic estimates of TF-CBT efficacy trial outcomes for PTS (*g* = 1.48, 95% CI [0.83, 2.13]) and depression (g = 0.78, 95% [0.15, 1.41])–but were significantly *better* than client outcomes of other TF-CBT community initiatives that used different implementation models (i.e., Lang et al., 2015; Rudd et al., 2019), to a small-to-large degree for PTS (LL *d*-difference = 0.34–0.81) and a small degree for depression outcomes (LL *d*-difference = 0.22).

**Implications for D&I Research:**

Collectively, these results suggest that CBLC implementation strategies may help to not only improve community access to TF-CBT and other EBTs but may also better ensure that the quality and outcomes of those EBTs remain high when implemented in community settings.

**Primary Funding Source:** Duke Endowment

### S3 Implementation of a trauma center-based, technology-enhanced stepped care mental health program for traumatic injury survivors

#### Tatiana Davidson^1^, Leigh Ridings^1^, Hannah Espeleta^2^, Olivia Bravoco^1^, Kristen Higgins^3^, Kenneth Ruggiero^1^

##### ^1^Medical University of South Carolina, Charleston, SC, USA; ^2^Medical University of SC, Charleston, SC, USA; ^3^Medical University of South Carolina, Charleston, USA

###### **Correspondence:** Tatiana Davidson (davidst@musc.edu)

**Background:**

Annually, over 600,000 adults served in U.S. trauma centers (≥ 20%) develop posttraumatic stress disorder (PTSD) and/or depression in the first year after injury. American College of Surgeons guidelines strongly recommend screening and addressing mental health recovery in traumatic injury patients. The *Trauma Resilience and Recovery Program (TRRP*) is a scalable and sustainable, technology-enhanced stepped model of care – one of the few in the US - that provides early intervention and direct services after traumatic injury via 4 steps: education, risk screening, and brief intervention at the bedside (Step 1); symptom self-monitoring via text messaging (Step 2); mental health screening at 30 days by chatbot or phone (Step 3); and, when appropriate, referral to mental health treatment (Step 4). This presentation describes the TRRP implementation process, program acceptability, and preliminary dissemination roadmap.

**Methods:**

We used the Exploration, Preparation, Implementation, Sustainment (EPIS; Aarons et al., 2011) model to implement TRRP in four Level I-II trauma centers. First, we collaborated with center stakeholders to assess trauma center’s needs, resources and workflow to identify implementation strategies (Exploration). Second, we worked with stakeholders to outline an implementation plan, taking into account center resources, workflow, and barriers to implementation to identify program adaptations (Preparation). Third, we implemented TRRP and addressed factors associated with engagement at the patient, provider, and center level (Implementation). Finally, we implemented strategies to promote long-term sustainability (Sustainability).

**Findings:**

These programs have reached more than 10,000 patients, we identified a high prevalence of PTSD and depression after discharge, and observed high patient engagement. Several lessons were learned that shaped our implementation protocol, including: model adaptations are needed for integration into center infrastructure, and early application of billing and reimbursement practices are critical to enhancing buy-in during the initial stages of implementation and promoting long-term sustainability.

**Implications for D&I Research:**

Trauma-center based, sustainable models of mental health care are needed to ensure that all patients receive the full range of services that they need. This study explored best-practice strategies for implementing and sustaining TRRP with the goal of identifying strategies to maximize adoption and sustained use of behavioral health programs in trauma centers.

**Primary Funding Source:** Duke Endowment Foundation

### S4 Multidisciplinary team functioning and performance in child advocacy centers: Associations with implementation outcomes

#### Elizabeth A. McGuier^1^, Scott D. Rothenberger^1^, Kara Byrne^2^, Kristine A. Campbell^2^, Brooks Keeshin^2^, David Kolko^1^

##### ^1^University of Pittsburgh School of Medicine, Pittsburgh, PA, USA; ^2^University of Utah, Salt Lake City, UT, USA

###### **Correspondence:** Elizabeth A. McGuier (millerea3@upmc.edu)

**Background:**

Child Advocacy Centers (CACs) use multidisciplinary teams (MDTs) to coordinate interagency responses to child abuse allegations. In this team-based setting, implementation of new practices is likely to be affected by teamwork quality. Mental health screening and referral protocols can increase accurate identification of children’s mental health needs and facilitate access to evidence-based treatment but are infrequently used in CACs. This study tests associations between teamwork quality and implementation outcomes during a statewide initiative to implement a standardized screening and referral protocol for traumatic stress and suicidality in CACs.

**Methods:**

MDT members (N = 433) from 21 CACs completed 5 validated team functioning measures (Affective: liking/trust, psychological safety; Behavioral: learning behavior, coordination about mental health care; Cognitive: clear direction) and 2 team performance measures (overall performance; mental health care quality). Implementation outcomes were the protocol’s acceptability, appropriateness, and feasibility. Team members rated all measures on Likert scales. Implementation timing varied across CACs; the survey occurred 1-18 months after initial training.

The first three models tested associations of team functioning with implementation outcomes (acceptability, appropriateness, feasibility). Then we tested associations of each team performance measure with outcomes. Multilevel models accounted for clustering within CACs.

**Findings:**

For feasibility, there were significant (*p*<.05) associations with liking/trust (unstandardized B=.19) and coordination about mental health care (B=.23). Similarly, for acceptability and appropriateness, there were marginal (*p*<.10) associations with liking/trust (B=.17; B=.16) and significant associations with coordination about mental health care (B=.27; B=.22). Psychological safety, learning behavior, and clear direction were not associated with any outcome, perhaps because of high intercorrelations between team functioning measures (*r*’s=.45-.71).

Team member-rated performance was significantly associated with acceptability (B=.10), appropriateness (B=.10), and feasibility (B=.09). Similarly, mental health care quality was significantly associated with acceptability (B=.15), appropriateness (B=.18), and feasibility (B=.24).

**Implications for D&I Research:**

Team performance and aspects of affective and behavioral team functioning are associated with perceived acceptability, appropriateness, and feasibility of a mental health screening protocol in a multidisciplinary team-based setting. Future analyses will test associations of teamwork and CAC-level outcomes (i.e., adoption, reach). Implementation strategies targeting teamwork may improve teams’ capacity to implement evidence-based practices and the quality of care in team-based settings.

**Primary Funding Source:** National Institutes of Health

### S5 Cost-effectiveness of a novel Medicaid reimbursement strategy for sustaining collaborative care in primary care clinics across New York State

#### Nathalie Moise^1^, Tod Mijanovich^2^, Amy Jones^3^, Danielle Gadbois^3^, John Billings^4^, Jay Carruthers^3^

##### ^1^Columbia University Irving Medical Center, New York, NY, USA; ^2^NYU Steinhardt School, New York, NY, USA; ^3^New York State, Albany, NY, USA; ^4^New York University Wagner Graduate School of Public Service, New York, NY, USA

###### **Correspondence:** Nathalie Moise (nm2562@cumc.columbia.edu)

**Background:**

Experts maintain that external facilitation and financial incentives are essential to successful collaborative care (CC) implementation. Few studies explore real-world cost implications, particularly from payor perspectives. In 2015, the New York State (NYS) Office of Mental Health created the CC Medicaid Program (CCMP) to support implementation and sustainability. CCMP, now covering ~350 primary care clinics, includes reimbursement and technical assistance. We assessed CCMP impact on healthcare costs and utilization.

**Methods:**

We used NYS Medicaid claims data from 2014 - 2019. Eligible patients identified by CCMP rate codes were ≥18 years old with ≥1 follow-up year. We used 5:1 Mahalanobis distance matching with replacement and entropy balance weighting to select comparison individuals similar to participants on demographics, and on healthcare costs, utilization and depression diagnosis in a 12-month baseline period. Outcome models were adjusted for baseline utilization and cost and number of Medicaid enrolled months. We conducted subgroup analyses by age (≥55 vs. <55), race/ethnicity, gender, and supplemental security income (SSI) status.

**Findings:**

We identified 6695 CCMP patients. Patients were on average 44 (SD=15.1) years old; 77.0% were female, 28.4% black, 34.8% Hispanic; 30.2% on SSI. During the baseline period, 36.5% had a depression diagnosis and 21.3% had a serious mental illness diagnosis. We identified 36,028 matched comparison group patients. The CCMP program cost $500,000/year for technical assistance/external facilitation and $150/patient/month in claims. In follow-up year 1, the CCMP (vs. comparison) group saw nonsignificant total cost-savings (-$290/patient, 95%CI -$686, $105, p=0.15), which improved by year three (-$1226/patient, 95%CI -$2,106, -$345, p=0.006), particularly amongst males (p-interaction=0.017) and those younger (p=0.007) or without diabetes/heart disease (p=0.003). For every follow-up year, CCMP participants had significantly higher Medicaid enrolled months (p<0.001), particularly amongst black, Hispanic and “other” race patients.

**Implications for D&I Research:**

Overall, the CCMP strategy extended Medicaid coverage and reduced costs by year 3. Our results are limited by reliance on submitted claims and non-randomized design. Nonetheless, our results may inform other centralized implementation efforts, the costs of which may be outweighed by long-term cost savings.

**Primary Funding Source:** Agency for Healthcare Research and Quality

### S6 Collaboration strategies for implementing cross-system interventions with child welfare and behavioral health organizations

#### Alicia Bunger^1^, Rebecca Phillips^1^, Emmeline Chuang^2^, Amanda Girth^1^, Erica Magier^1^, Jared Martin^1^, Rebecca Smith^1^, Kathryn Lancaster^1^

##### ^1^The Ohio State University, Columbus, OH, USA; ^2^University of California, Berkeley, Berkeley, CA, USA

###### **Correspondence:** Alicia Bunger (bunger.5@osu.edu)

**Background:**

Cross-system interventions that integrate social and behavioral health services can improve client outcomes and expland community impact. Successful implementation of cross-system interventions depends on extent to which partners are able to align front-line services, organizational operations, and system-level approaches. However, collaboration strategies and other relational “bridging factors” linking diverse implementation contexts have received limited empirical attention. This study identifies and classifies multi-level collaboration strategies used during implementation of Ohio START, a cross-system intervention designed to integrate child welfare and behavioral health services for families that are involved with child welfare due to parental substance misuse.

**Methods:**

We used a multiple case study design with 17 counties that implemented Ohio START. Qualitative data were gathered from two sources: 1) formal partnership agreements (e.g. contracts, Memoranda of Understanding); and 2) 48 small group interviews about collaborative approaches to implementation conducted with 104 staff from child welfare agencies, behavioral health treatment organizations, and regional behavioral health boards involved in implementation. To examine collaboration responsibilities and impact, qualitative data were analyzed using an iterative template approach and content analysis methods.

**Findings:**

Across the 17 counties, ten types of collaboration strategies in support of program goals were identified. At the administrative and front-line levels, these strategies included establishing formal inter-organizational agreements to (1) expedite access to substance abuse treatment for START families, (2) contract for needed expertise, and (3) share case-level data; (4) joint supervision of START staff; (5) co-location of staff; (6) establish clear frontline referral protocols; and (7) promote child welfare caseworker and behavioral health therapist participation in shared decision-making meetings. At the system level, we found that regional coordinating bodies (e.g. public behavioral health boards) could also support cross-sector collaboration by: (8) sharing information; (9) brokering relationships; and (10) providing funding.

**Implications for D&I Research:**

We identified a range of collaboration strategies used at multiple levels during cross-system intervention implementation. With no standard approach to collaborating for implementation, future studies are needed to determine the specific factors significant for collaboration strategy selection and effectiveness in improving implementation, service delivery, and client outcomes.

**Primary Funding Source:** National Institutes of Health

### S7 Inter-agency collaboration is associated with increased frequency of research use in children’s mental health policymaking

#### Jonathan Purtle^1^, Katherine Nelson^2^, Rebecca Lengnick-Hall^3^, Sarah Horwitz^4^, Lawrence Palinkas^5^, Mary McKay^6^, Kimberly Hoagwood^4^

##### ^1^Health Management &Policy, Drexel University School of Public Health, Philadelphia, PA, USA; ^2^3rd Floor, Dornsife School of Public Health at Drexel University, Philadelphia, PA, USA; ^3^Washington University, St. Louis, MO, USA; ^4^New York University, New York, NY, USA; ^5^University of Southern California, Los Angeles, CA, USA; ^6^Washington University at St Louis, St Louis, MO, USA

###### **Correspondence:** Jonathan Purtle (jpp46@drexel.edu)

**Background:** Inter-agency collaboration among direct services providers is a common target of dissemination and implementation (D&I) strategies focused on the implementation of evidence-based mental health services. Little research, however, has examined the effect of inter-agency collaboration among agency leaders on the use of research evidence in policymaking related the mental health systems. To address this knowledge gap, we sought to determine whether the frequency of mental health agency officials’ (MHA) inter-agency collaboration about children’s mental health issues was associated with the frequency of using research evidence in children’s mental health policymaking, after adjusting for well-established barriers/facilitators to research use in policymaking.

**Methods:** Data were collected from state MHA officials through a web-based survey immediately pre-COVID-19 (N= 221) and a survey of county MHA officials during COVID-19 (N= 117). The primary independent variable was a composite score quantifying the frequency of collaboration about children’s mental health issues between officials in MHAs and six state agencies: substance use, public health, education, child welfare, juvenile justice, and Medicaid. The dependent variables were composite scores quantifying the frequency research use in children’s mental health policymaking in general and for specific purposes (i.e., conceptual, instrumental, tactical, imposed). Covariates were composite scores quantifying well-establihsed barriers/facilitators to research use (e.g., agency leadership supporting research use, skills for research use). Separate multiple linear regression models estimated associations between frequency of inter-agency collaboration and research use scores, adjusting for other barriers/facilitators to research use.

**Findings:** Frequency of inter-agency collaboration was positively and independently associated with the frequency of research use in children’s mental health policymaking among state (β= 0.22, p=. 004) and county (β= 0.39, p<.0001) MHA officials. Inter-agency collaboration was also the only variable significantly associated with the frequency of research use for all four specific purposes among state MHA officials. The magnitudes of associations between inter-agency collaboration and frequency of research use were generally stronger than for more well-established determinants of research use in policymaking.

**Implications for D&I Research:** Strategies that promote inter-agency collaboration are promising targets for D & I strategies that aim to increase the use of research evidence in children’s mental health policymaking.

**Primary Funding Source:** National Institutes of Health

### S8 Theory-informed pre-implementation enhancement strategy to promote staff's intentions to implement evidence-based practices and youth behavioral outcomes

#### Yanchen Zhang^1^, Clayton Cook^2^, Aaron Lyon^3^, Olivia Lickteig^2^, Laura Janzen^2^, Jordan Thayer^2^

##### ^1^University of Iowa, Iowa City, IA, USA; ^2^University of Minnesota, Minneapolis, MN, USA; ^3^University of Washington, Seattle, WA, USA

###### **Correspondence:** Yanchen Zhang (yanchen-zhang@uiowa.edu)

**Background:**

Training and follow-up consultation alone are proven insufficient to facilitate successful implementation of evidence-based practices and programs (EBPPs) and often lead to languishing outcomes. Theory-informed *Pre-Implementation Enhancement Strategies* (PIES) bear the potential to amplify the effect of training and consultation on improving implementation and client outcomes. In social cognitive and implementation theories, individual characteristics (e.g., intentions to implement; ITI) represent putative processes that explain the discrepancy between professionals’ enactment of implementation behaviors against received training and consultation. We used a double-masked randomized controlled trial to examine the efficacy of a brief PIES targeting staff's ITI to improve EBPP implementation in school-based behavioral health.

**Methods:**

We randomly selected participants from two urban schools in the Northwest, which had no prior experience with universal EBPPs, then randomly assigned them to the *treatment* (PIES; n _treatment_ = 22) or *active-control condition* (regular training; n _control_ = 21). The condition assignment remained unknown to either the participants or researchers evaluating outcomes. Baseline equivalences between the two groups regarding key variables were established at pre-test. Several measures were used for pre-/post-tests about staff's ITI, implementation fidelity, academic engaged time (AET; Chafouleas, 2011). Mixed-factor ANOVAs (within-subject factor = pre-/post-tests; between-subject factor = condition) were performed for each outcome (ITI, fidelity, and AET).

**Findings:**

The treatment effects of PIES on ITI, fidelity, and AET were all significant (ITI: *F* (1, 41) = 7.02, *p* < .01; Fidelity: *F* (1, 41) = 18.50, *p* < .01; AET: *F* (1, 41) = 5.07, *p* < .05). It was not until the completion of PIES that the outcomes improved for staff in the *treatment condition*, with little change for those in the *control condition*.

**Implications for D&I Research:**

An implicit yet faulty common assumption is that staff already have favorable intention/motivation to implement new EBPPs before or after training and consultations. Our findings suggested that schools should strategically intervention on staff’s *intentions to implement* to amplify the effectiveness of common implementation strategies such as training and follow-up consultation, which will lead to favorable changes in implementation behaviors (e.g., fidelity) and client behavioral outcomes. Details about how to carry out PIES will be discussed.

### S9 First episode digital monitoring: A qualitative study to inform a novel mHealth intervention for psychosis

#### Ana Stefancic^1^, Russell Rogers^2^, Sarah Styke^2^, Ilana Nossel^2^, Leopoldo J. Cabassa^3^, Scott Stroup^2^, David Kimhy^4^

##### ^1^Columbia University, New York, NY, USA; ^2^New York State Psychiatric Institute, New York, NY, USA; ^3^Washington University in St. Louis, St. Louis, MO, USA; ^4^Icahn School of Medicine at Mount Sinai, New York, NY, USA

###### **Correspondence:** Ana Stefancic (as2463@columbia.edu)

**Background:**

Mobile health (mHealth) applications using patient questionnaires to gather real-time information have been used extensively in psychosis research. However, their use in treatment has been limited, with few studies aiming to enhance integration of mHealth data into “real world” clinical settings. This qualitative study solicited service providers’ input to inform adaptations to an app-based mHealth intervention within first-episode psychosis (FEP) treatment for young adults prior to implementation of a pilot trial.

**Methods:**

Researchers developed an initial mHealth intervention consisting of app-based questionnaires that could solicit information from patients (e.g., symptoms, side-effects) and provide data reports to prescribers to enhance treatment. Semi-structured interviews with 11 FEP treatment team providers at three clinical sites elicited their input on planned implementation procedures and the intervention. Data sources consisted of interview summaries, interview transcripts, and notes from research meetings when adaptations were deliberated. We conducted matrix analysis to categorize providers’ suggested adaptations using the Framework for Reporting Adaptations and Modifications to Evidence-based interventions (FRAME) and tracked whether adaptations were made, and when applicable, why not.

**Findings:**

Suggestions to add/refine content were most common, including re-wording questionnaires to reflect person-centered vs. medical language and presenting more information in clinician reports. Adaptations to context were most often related to an implementation strategy, how report data were displayed, and with whom reports were shared. Reasons for suggesting modifications included responsiveness to patients’ motivation and readiness (e.g., time burden), providers’ clinical judgment (e.g., need for clinically relevant information), and organizations’ mission/culture (e.g., shared decision-making principles). Adaptations not made reflected suggestions to collect additional patient information, facilitate patients’ access to their own data, and change timing of intervention components (e.g., tailoring ping frequency). Reasons for not incorporating suggestions included additional resources being required, modifications being beyond intervention scope, concerns regarding diluting intervention components, and managing participant time burden.

**Implications for D&I Research:**

This study illustrates a pragmatic application of the FRAME to track provider-suggested adaptations to an mHealth intervention and its implementation within FEP treatment to increase alignment with key patient, provider, and organizational factors. By tracking adaptations suggested but not made and reasons why, it provides further insight into adaptation decision-making processes.

**Primary Funding Source:** National Institutes of Health

### S10 Whose opinion matters most? the differential perspectives of providers and leaders on implementation readiness

#### Rachel Kim^1^, Angelina Ruiz^1^, Daniel Cheron^1^, Amy Doyle^2^, Kerry O'Loughlin^1^

##### ^1^Judge Baker Children's Center, Boston, MA, USA; ^2^Judge Baker Children’s Center, Boston, MA, USA

###### **Correspondence:** Rachel Kim (rkim@jbcc.harvard.edu)

**Background:**

Organizational readiness and capacity impact implementation outcomes. Participants’ perspectives of readiness may have a differential impact on outcomes based on their role within the initiative. This session will expand prior work (Kim et al., 2019; Kim et al., 2020) to highlight implementation and client engagement outcomes associated with behavioral health clinician and senior leader perspectives of readiness and capacity during the active implementation phase in five government-funded Modular Approach to Therapy for Children (MATCH) Learning Collaboratives with publicly-funded children’s community mental health centers (CMHCs).

**Methods:**

The adapted Readiness Diagnostic Scale, based on Scaccia and colleagues (2015) *R=MC*^*2*^ heuristic of organizational readiness (readiness for implementation = an organization’s *motivation*, *general organizational capacities*, and *innovation-specific capacities*), was completed by 135 clinicians and 56 senior leaders representing 27 CMHCs early during active implementation of 5 learning collaboratives (i.e., shortly following the MATCH clinical training). Implementation metrics for each CMHC were collected during the active implementation and sustainability phases. Correlations between clinician and senior leader readiness scores and implementation outcomes were analyzed to explore the differential impact of readiness and capacity based on participant role.

**Findings:**

Clinician perspectives of readiness and capacity were more strongly and frequently associated with implementation outcomes than those of senior leaders. Specifically, clinicians’ views on organizational leadership, staff capacity, and process capacity most often and most strongly correlated with implementation outcomes. For example, better perspectives on these three subdomains were related to more frequent therapy sessions and more frequent client and caregiver outcome survey completion. Ten other clinician-reported readiness subdomains were associated with implementation outcomes. Only organizational culture, resource utilization, and leadership as reported by senior leaders mildly or moderately correlated with two of six implementation outcomes examined.

**Implications for D&I Research:**

Understanding the impact of clinicians’ perspectives of organizational readiness and capacity on implementation outcomes is crucial. Readiness and capacity assessed during active implementation can inform tailored consultation and quality improvement targets throughout the Learning Collaborative, such as supporting organizations in developing strategies to improve process capacities, or the organizational ability to strategize, implement, evaluate, and improve. Implications for providing tailored consultation to initiative participants in different roles will be discussed.

**Primary Funding Source:** State and County Department of Health and Human Services

### S11 Effects of economic information and local data on state legislator engagement with behavioral health dissemination materials: A cluster-randomized dissemination experiment

#### Jonathan Purtle^1^, Katherine Nelson^2^, Luwam Gebrekristos^3^, Felice Le-Scherban^4^, Sarah Gollust^50^

##### ^1^Health Management &Policy, Drexel University School of Public Health, Philadelphia, PA, USA; ^2^3rd Floor, Dornsife School of Public Health at Drexel University, Philadelphia, PA, USA; ^3^Drexel University, Philadelphia, PA, USA; ^4^Epidemiology and Biostatistics, Drexel University School of Public Health, Philadelphia, PA, USA; ^5^University of Minnesota, Minneapolis, MN, USA

###### **Correspondence:** Jonathan Purtle (jpp46@drexel.edu)

**Background:** State legislators make decisions that affect behavioral health systems and outcomes. Effective dissemination of behavioral health evidence can increase the likelihood that these decisions are aligned with evidence; and effective dissemination requires end-users to engage with dissemination materials. Based on prior research and the elaboration likelihood model of persuasive communication, we tested the hypothesis that inclusion of economic evidence and local data would increase legislator engagement with behavioral health dissemination materials.

**Methods:** A pre-registered, three-arm cluster-randomized dissemination experiment was conducted. A university researcher sent two personalized e-mails (two weeks apart) containing evidence summaries and policy brief links about child maltreatment and behavioral health problems to state legislators (12,691 e-mails delivered to 6,523 legislators). The e-mail subject lines, text, and policy brief content were manipulated across the study arms. Legislators in the intervention condition received evidence about the incidence of child maltreatment cases in their state and the economic impacts of these cases for their state’s public systems, the enhanced control condition received state child maltreatment incidence evidence but not economic evidence, and the control condition received national child maltreatment incidence evidence and no economic evidence. Outcomes were rates of e-mail views, policy brief link clicks, requests for researcher consultation, and mentions of child maltreatment terms in legislators’ social media posts.

**Findings:** For the first e-mail, the e-mail view rate was 42.6% higher in the intervention than enhanced control condition (22.8% vs. 14.8%) and 20.8% higher than in the control condition (22.8% vs. 18.5%) (both p<.0001). Similar results were observed for the second e-mail. These differences remained significant after adjustment in single-level models, but not multi-level models adjusting for state clustering. There was a significant interaction between experimental condition and political party (p<.0001) in which the intervention increased e-mail view rates among Democrats but not Republicans. The experimental condition had no effect on policy brief link clicks, requests for consultation, or social media posts.

**Implications for D&I Research:** Inclusion of economic information and local data in dissemination materials can increase engagement with behavioral health evidence e-mailed from a university researcher among Democrat, but not Republican, legislators. Dissemination strategies tailored for legislators’ political party affiliation may be needed.

**Primary Funding Source:** The Robert Wood Johnson Foundation

### S12 A pilot study of participatory and rapid implementation approaches to increase depression screening in primary care

#### Briana Last^1^, Alison Buttenheim^2^, Anne Futterer^3^, Cecilia Livesey^4,5^, Jeffrey Jaeger^3^, Rebecca Stewart^3,6^, Megan Reilly^3^, Matthew Press^3,7^, Maryanne Peifer^3^, Courtney Benjamin Wolk^3^, Rinad Beidas^8,9,10,11^

##### ^1^University of Pennsylvania, Philadelphia, PA, USA; ^2^423 Guardian Drive, Perelman School of Medicine, University of Pennsylvania, Philadelphia, PA, USA; ^3^Perelman School of Medicine, University of Pennsylvania, Philadelphia, PA, USA; ^4^UnitedHealth Group, Philadelphia, PA, USA; ^5^Department of Psychiatry, Perelman School of Medicine University of Pennsylvania, Philadelphia, PA, USA; ^6^Penn Implementation Science Center at the Leonard Davis Institute of Health Economics (PISCE@LDI), Philadelphia, PA, USA; ^7^Primary Care Service Line, Penn Medicine, Philadelphia, PA, USA; ^8^Perelman School of Medicine at the University of Pennsylvania, Philadelphia, PA, USA; ^9^University of Pennsylvania, Leonard Davis Institute of Health Economics, Philadelphia, PA, USA; ^10^University of Pennsylvania, Penn Medicine, Philadelphia, PA, USA; ^11^University of Pennsylvania, Perelman School of Medicine, Philadelphia, PA, USA

###### **Correspondence:** Briana Last (brishiri@sas.upenn.edu)

**Background:** Most individuals with depression go unidentified and untreated. In 2016 the US Preventive Services Task Force released guidelines recommending universal screening in primary care to identify patients with depression and to link them to treatment. Feasible, acceptable, and effective strategies to implement these guidelines are needed.

**Methods:** This three-phased study employed rapid participatory methods to design and test strategies to increase depression screening at Penn Medicine, a large health system with 90 primary care practices. First, researchers solicited ideas and barriers from stakeholders to increase screening using an innovation tournament—a crowdsourcing method that invites stakeholders to submit ideas to address a workplace challenge. Second, a panel of stakeholders and scientists deliberated over and ranked the tournament ideas. An instant runoff election was held to select the winning idea. Third, the research team piloted the winning idea in a primary care practice using rapid prototyping, an approach that quickly refines and iterates strategy designs.

**Findings:** The innovation tournament yielded 31 ideas and 32 barriers from diverse stakeholders (12 primary care physicians, 10 medical assistants, 4 nurse practitioners, 2 practice managers, and 4 patient support assistants). A panel of 6 stakeholders and scientists deliberated on the ideas and voted for patient self-report (i.e., through tablet computers, text message, or an online patient portal) as the winning idea. The research team rapid prototyped tablets in one primary care practice with one physician over 5 five-hour shifts to examine the feasibility, acceptability, and effectiveness of the strategy. Most patients, the physician, and medical assistants found the tablets acceptable and feasible. However, patient support assistants struggled to incorporate them in their workflow and expressed concerns about scaling up the process. Depression screening rates were higher using tablets compared to usual care; follow-up was comparable between tablets and usual care.

**Implications for D&I Research:** Rapid participatory methods engaged and amplified the voices of diverse stakeholders in primary care. These methods helped design an acceptable and feasible implementation strategy that showed promise for increasing depression screening in a primary care setting. The next step is to evaluate the strategy in a randomized controlled trial across primary care practices.

**Primary Funding Source:** National Institutes of Health

### S13 Developing an implementation facilitation strategy for a mental health screening and ehealth positive affect intervention for people living with HIV

#### Kristen Ethier^1^, Tammy Stump^2^, Lisa Hirschhorn^3^, Andrea Dakin^4^, Nora Bouacha^4^, Angela Freeman^2^, Jacqueline Bannon^2^, Walter Gomez^5^, Judith Moskowitz^2^, Alida Bouris^1^

##### ^1^University of Chicago, Chicago, IL, USA; ^2^Northwestern University, Chicago, IL, USA; ^3^Northwestern University Feinberg School of Medicine, Chicago, IL, USA; ^4^AIDS Foundation of Chicago, Chicago, IL, USA; ^5^University of Illinois at Chicago, Chicago, IL, USA

###### **Correspondence:** Kristen Ethier (kethier@uchicago.edu)

**Background:** Depression is common in people living with HIV and is a significant barrier to optimal engagement in HIV care. Further, mental health treatment access remains a significant challenge, worsened by COVID-19. Positive affect, independent of depression, predicts slower disease progression, better medication adherence, and a higher likelihood of viral suppression. Thus, eHealth interventions to increase positive affect may support more effective engagement in HIV care and reduce access barriers to mental health treatment. This study presents findings from pre-implementation research guided by the Consolidated Framework for Implementation Research (CFIR) to develop system-wide implementation strategies for the clinic-based implementation of a behavioral health screener and eHealth positive affect intervention for people living with HIV and depression.

**Methods:** We analyzed survey and interview data from staff at 16 HRSA-funded Ryan White Medical Case Management sites in Chicago. Medical case managers (MCMs) and supervisors completed a survey covering CFIR domains. Survey data informed a purposive sampling frame and CFIR-driven interview protocol with MCMs and supervisors. Interview data were analyzed using Rapid Qualitative Analysis to inform an Implementation Research Logic Model (IRLM) and the selection of implementation strategies.

**Findings:** Survey respondents (n=58; 68% response) had fairly positive views of the inner setting, including team culture, learning climate, and implementation readiness (*M*s=3.81-3.88 on a 5-point scale). Other potential implementation barriers included intervention complexity (*M*=2.54), human resource cost (*M*s=2.91-3.33), and a moderate relative advantage (*M*s=2.44-2.81). Interview results identified additional barriers in the patient and clinic domains, which informed refinements in the implementation strategies, including: training on using the intervention to complement co-located behavioral health services (relative advantage), ongoing attention to equity barriers to accessing the eHealth platform (address patient needs and resources), and regular feedback to each clinic regarding referral processes (rewards/incentives). These findings informed the development of an initial IRLM and implementation plan that will be evaluated during the implementation phase.

**Implications for D&I Research:** Pre-implementation evaluation using the CFIR model can serve as a useful guide to adapt strategies before implementation, potentially boosting the reach and effectiveness of interventions for people living with HIV and depression.

**Primary Funding Source:** National Institutes of Health

### S14 Characteristics of workforce development trainings and technical assistance that are associated with implementation outcomes among school mental health practitioners

#### Jonathan Olson^1^, Marianne Kellogg^1^, Kelcey Schmitz^1^, Eric Bruns^2^

##### ^1^University of Washington, Seattle, WA, USA; ^2^Department of Psychiatry and Behavioral Sciences, University of Washington School of Medicine, Seattle, WA, USA

###### **Correspondence:** Jonathan Olson (jro10@uw.edu)

**Background:**

Models of implementation science have highlighted the role of workforce development in supporting evidence-based behavioral health practices. The current study focused on the impact of training and technical assistance (TA) provided to School Mental Health (SMH) professionals by the Northwest Mental Health Technology Transfer Center (MHTTC). Specific goals were to: 1) Identify characteristics of training/TA efforts associated with outcomes; 2) Determine if outcomes vary across demographics; and 3) Investigate if associations vary by context.

**Methods:**

Participants were 2,941 SMH administrators, teachers, counselors, and direct service providers who participated in one or more MHTTC training/TA sessions since 2019. Outcomes included self-reported intended use of training/TA content and changes in mastery of training content. Independent variables included gender, race/ethnicity, education, perceived quality of training/TA, degree to which the training/TA challenged organizational practices, perceived importance of the training/TA, training format (in-person versus online), and timing (pre- versus post-COVID-19 restrictions).

**Findings:**

Results of multiple regression models suggest that the following variables were associated with both intended use and content mastery: 1) Education level (β_*use*_ = -.054, p < .01; β_*mastery*_ = - .065, p < .001); 2) Event quality (β_*use*_ = .363, p < .001; β_*mastery*_ = .267, p < .001); 3) Challenge to organizational practices (β_*use*_ = .088, p < .001; β_*mastery*_ = .083, p < .001). In addition, the following were related to intended use: 4) BIPOC race/ethnicity (β_*use*_ = .045, p < .01); 5) Perceived importance of topic (β_*use*_ = .152, p < .001). Training/TA formats and timing were unrelated to outcomes, and interaction effects among independent variables and demographics were non-significant.

**Implications for D&I Research:**

The lack of differences across training/TA formats suggest that participants gain benefits from both in-person and online support. This is encouraging since a previous study showed that an increase in online MHTTC formats in response to COVID-19 resulted in increased attendance among BIPOC participants and workforce members with lower levels of education. Our current results suggest that members of these groups experienced positive outcomes. Furthermore, these findings underscore the importance of implementing high-quality support that matches the needs of intended audiences and challenges current practices rather than repeating the status quo.

**Primary Funding Source:** Substance Abuse and Mental Health Services Administration

### S15 Implementation and student goal attainment outcomes of compass training

#### Lindsey Ogle^1^, Lisa Ruble^1^, John McGrew^2^

##### ^1^Ball State University, Muncie, IN, USA; ^2^Indiana University Purdue University Indianapolis, Indianapolis, IN, USA

###### **Correspondence:** Lindsey Ogle (lnogle@bsu.edu)

**Background:** Teachers are the primary intervention providers for students with autism, but many still face challenges designing effective intervention programs. The Collaborative Model for Promoting Competence and Success (COMPASS) is in an empirically based consultation intervention designed to support parents and teachers of students with autism in designing intervention plans focused on three social emotional learning skills (communication, social, and independent learning) followed by coaching with performance feedback to ensure fidelity of implementation and maximize student goal attainment. However, in all previous studies the developers acted as COMPASS consultants. Thus, the primary aim of this study was to develop and attempt to replicate equivalent implementation outcomes using a COMPASS training package for COMPASS-naïve school-based consultants. A secondary aim was to understand the impact of coaching feedback type (face-to-face coaching vs. written feedback) and dosage (1 or more than 1) vs. those who received the initial consultation but no coaching feedback.

**Methods:** Participants were recruited across two school years resulting in a total of nine consultant trainees (CT) and 28 teacher/parent/student triads. Each CT consulted with 2-4 teacher/parent/student triads, and each triad was randomly assigned to one of four different dosage and type conditions following their initial consultation.

**Findings:** Findings suggest that our training package was effective in training school-based consultants to implement COMPASS with high fidelity (adherence and quality of delivery), acceptability, feasibility, and appropriateness. With respect to coaching, electronically delivered written performance feedback of intervention plan adherence and student goal attainment was equally effective to face-to-face coaching and increased opportunities for performance feedback (2 or 4) produced stronger results compared to having only one or no opportunities.

**Implications for D&I Research:** Consultation interventions such as COMPASS are an innovative method to improve the uptake of EBPs by teachers, improve the behavioral health of students with autism, and help to address disparities regarding lack of access to high quality intervention. Additionally, our findings that written reports had similar outcomes to face-to-face coaching may make the process of coaching teachers more feasible for consultants.

**Primary Funding Source:** National Institutes of Health

## Building the Future of D&I Science: Training, Infrastructure, and Emerging Research Areas

### S16 Teaching for implementation: A framework for building implementation research and practice capacity within the translational science workforce

#### Aaron Leppin^1^, Rachel Shelton^2^, Kathleen Stevens^3^, Brittany Rudd^4^, Ana Baumann^5^, Bethany Kwan^6^, David Warner^7^, Maria Fernandez^8^

##### ^1^Mayo Clinic, Rochester, MN, USA; ^2^Columbia University Mailman School of Public Health, New York, NY, USA; ^3^University of Texas Health San Antonio, San Antonio, TX, USA; ^4^University of Illinois at Chicago, Chicago, IL, USA; ^5^Washington University in St. Louis, St. Louis, MO, USA; ^6^University of Colorado Anschutz Medical Campus, Aurora, CO, USA^7^Mayo Clinic, Rochester, Rochester, MN, USA; ^8^The University of Texas Health Science Center at Houston School of Public Health, Houston, TX, USA

###### **Correspondence:** Brittany Rudd (bnrudd@uic.edu)

**Background:** Implementation is core to evidence translation and public health impact. As such, many translational science stakeholders—including Clinical and Translational Science Award hubs (CTSAs) --are seeking to recruit, teach, and train an implementation science workforce. The type of workforce that will make implementation happen consists of both implementation researchers and practitioners, yet little guidance exists on how to train such a workforce.

**Methods:** We—members of the Advancing Dissemination and Implementation Sciences in CTSAs Working Group—were commissioned to write a special communication concerning best practices and opportunities for teaching implementation science within the CTSA infrastructure. Over a period of 10 months, we reviewed the existing literature and shared experiences via email communication and Zoom meetings. We created initial outlines, sought feedback from translational science stakeholders, and drafted and edited outlines, figures, and manuscript text.

**Findings:** Our writing group consisted of 8 experts in teaching implementation and translational science from 7 institutions and representing diverse career stages. Early on, we committed to the idea that translation requires both implementation research and practice. We acknowledged current limitations in implementation practitioner training and recognized opportunities for CTSAs and other “real-world” translational infrastructures (e.g. public health systems) to innovate. We ultimately developed the Teaching For Implementation (TFI) Framework. TFI highlights similarities and differences in role and competencies among researchers and practitioners. It shows how CTSAs—with cores dedicated to community engagement and education—are ideally positioned to train both groups together and in ways that prepare them for operationalizing real-world learning systems and contexts. It provides specific recommendations on including a more diverse group of learners in implementation training, prioritizing educational competencies that accomplish implementation tasks in real-world systems, strategies for adapting a common core curriculum to meet the diverse educational objectives of different learners, useful pedagogical methods such as researcher and practitioner co-learning opportunities, and strategies for infrastructure development (implementation labs, resource sharing) that support this approach.

**Implications for D&I Research:** Reliable evidence translation and public health impact depends on implementation researchers and practitioners working together and learning from each other. TFI provides a roadmap for teaching and training an implementation science workforce equipped to co-exist in this way.

**Primary Funding Source:** National Institutes of Health

### S17 Competencies for supporting evidence use: The role of trusting relationships in implementation

#### Allison Metz^1^, Todd Jensen^2^, Amanda Farley^3^, Leah Bartley^4^

##### ^1^UNC School of Social Work, Chapel Hill, NC, USA; ^2^University of North Carolina at Chapel Hill School of Social Work, Chapel Hill, NC, USA; ^3^FPG Child Development Institute, UNC Chapel Hill, Chapel Hill, NC, USA; ^4^National Implementation Research Network, Carrboro, NC, USA

###### **Correspondence:** Allison Metz (allison.metz@unc.edu)

**Background:** There is an increasing call for the advancement of a workforce capable of integrating implementation research – models, frameworks, and strategies – into practice to support evidence use, advance equity, and improve population outcomes. The shortage of individuals trained in the practice of implementation has been cited as a reason for failure to optimize evidence use. Metz and colleagues (2021) articulated 15 plausible competencies of implementation practice within three domains: (a) *co-creation* (i.e., active involvement of stakeholders throughout the implementation process), (b) *ongoing improvement* (i.e., deliberate use of data, information, and feedback to bolster implementation), and (c) *sustaining change* (i.e., building capacity to support implementation support recipients’ ongoing and autonomous use of evidence). This William T. Grant funded study explored the use of competencies by professionals who support evidence use in human service systems and the conditions under which specific implementation strategies were perceived as most effective.

**Methods:** A hybrid purposive-convenience sampling approach resulted in a sample of 17 individuals, each with more than 15 years’ experience providing implementation support. Data were collected via in-depth, semi-structured interviews. Core research questions included: What implementation support strategies are used to support the use of evidence? Under what conditions have specific implementation support strategies contributed to supporting evidence use? Data were analyzed using a qualitative content analysis approach.

**Findings:** Respondents reported using strategies across domains to support evidence-use, including co-creation and engagement, ongoing improvement and sustaining change. Trusting relationships emerged as a ubiquitous fixture of the implementation support process. Respondents described trusting relationships as directly associated with successful implementation and use of evidence and bidirectionally associated with (and reinforcing of) all other implementation strategies.

**Implications for D&I Research:** Findings reflect that implementation support is a multi-faceted endeavor that requires a broad range of skills. Respondents enacted technical strategies (e.g., supporting data use), while simultaneously carrying out relational strategies (e.g., building trusting relationship, addressing power differentials). Relationships appear to be as important as technical strategies and may explain why perfectly offered implementation support at times remains unsuccessful in leading to sustained evidence use. Building a workforce capable of supporting evidence-use will require developing skills for building trusting relationships.

**Primary Funding Source:** William T Grant Foundation

### S18 The TDR dissemination and implementation massive open online course (MOOC): Evaluation and lessons learned from seven low-and middle-income countries

#### Ashlin Rakhra^1^, Cole Hooley^2^, Meredith Fort^3^, Mary Beth Weber^4^, Mina Hosseinipour^5^, Manuel Ramirez^6^, Kezia Adjei^7^, Adamson S. Muula^8^, Annette Fitzpatrick^9^, Hoa L. Nguyen^10^, Victor Davila^11^, Josephine Andesia^12^, Kingsley Apusiga^7^, LeShawndra Price^13^, Pascal Launois^14^, Ana Baumann^15^

##### ^1^Department of Population Health, New York University Grossman School of Medicine, New York, NY, USA; ^2^Brigham Young University, Provo, USA; ^3^University of Colorado at Denver, Aurora, CO, USA; ^4^Emory University, Atlanta, GA, USA; ^5^Institute for Global Health and Infectious Diseases, University of North Carolina, Chapel Hill, NC, USA; ^6^Institute of Nutrition of Central America and Panama, Guatemala City, Guatemala; ^7^Kwame Nkrumah University of Science & Technology, Kumasi, Ghana; ^8^The University of Malawi, Zomba, Malawi; ^9^Departments of Epidemiology, Family Medicine, Global Health, School of Public Health, University of Washington, Seattle, WA, USA; ^10^University of Massachusetts Medical School, Worcester, MA, USA; ^11^Washington University School of Medicine, St. Louis, USA; ^12^Academic Model Providing Access to Healthcare (AMPATH), Eldoret, Kenya; ^13^National Institute of Allergy and Infectious Diseases, National Institutes of Health, Bethesda, MD, USA; ^14^World Health Organization, Geneva, MO, Switzerland; ^15^Washington University in St. Louis, St. Louis, MO, USA

###### **Correspondence:** Ashlin Rakhra (Ashlin.Rakhra@nyulangone.org)

**Background:**

Non-communicable diseases (NCDs) are a leading cause of morbidity and mortality in low-and middle-income countries (LMICs). Despite this, there is a lack of funding, training and mentorship for NCD investigators in LMICs. To address this, participants from the Global Research on Implementation and Translation Science (GRIT) consortia of studies in eight LMICs and their networks, attended the dissemination and implementation (D&I) massive open online course (MOOC) developed by the Special Programme for Research and Training in Tropical Diseases at the World Health Organization to strengthen capacity building and D&I research concepts. Here, we report on the feasibility of this MOOC, which was implemented during the SARS COVID-19 pandemic.

**Methods:**

Participants completed pre- and post- training questionnaires to assess D&I competencies, general research skills, and research mentor access and quality. D&I competencies were measured by use of a scale developed for a US-based training program, with change in competency scores assessed by paired *t* test. We used univariate statistics to analyze the data for all other outcomes.

**Findings:**

Of the 247 participants enrolled, 32 (13%) completed all MOOC components. D&I competency scores showed strong evidence of improvement for those who had complete pre- and post-competency scores. Trainee’s average score on the full competency scale improved 1.45 points (0-5 scale) from pre- to post-test; all four subscales also showed strong evidence of improvements. Trainee’s average competency for grant writing was 2.4 and 3.4 (0-5 scale) for proposal/ manuscript writing and presentations. 40% of trainees reported access to a research mentor, 12% reported access to a D&I specific mentor. Participants reported barriers (unstable internet access and challenges due to COVID-19) and facilitators (topical interests and course collaboration with colleagues) to completing the MOOC.

**Implications for D&I Research:**

Although COVID-19 affected program usage and completion, the MOOC was feasible and effective, showing that among LMIC participants completing the course, there was strong improvement in D&I competency scores. Recommendations for future D&I trainings in LMICs should include 1) adding more topic specific modules (i.e. NCD research, general research skills) for scalability; 2) fostering more collaboration with participants across LMICs; and 3) establishing partnerships with D&I mentors for course participants.

**Primary Funding Source:** National Institutes of Health

### S19 Highly motivated but lacking skills and collaborators to engage in health equity-oriented dissemination and implementation (D&I) research: Survey findings from health researchers regarding conducting health equity d&I research

#### Prajakta Adsul^1^, Ana Baumann^2^, Rajinder Sonia Singh^3^, Eva Woodward^4^, Rachel Shelton^5^

##### ^1^University of New Mexico, Albuquerque, NM, USA; ^2^Washington University in St. Louis, St. Louis, USA; ^3^South Central Mental Illness Research, Education and Clinical Center, Veterans Health Administration, AR, USA; ^4^VA Center for Mental Healthcare and Outcomes Research, North Little Rock, AR, USA; ^5^United States, Columbia Mailman School of Public Health, New York, NY, USA

###### **Correspondence:** Prajakta Adsul (padsul@salud.unm.edu)

**Background:** The striking racial and ethnic health disparities that have been exacerbated through the ongoing COVID-19 pandemic have encouraged the collective reflection of several scientific disciplines in considering the extent to which their research has focused sufficiently on addressing health disparities and promoting health equity. To make progress towards these goals in dissemination and implementation science (D&I), it is critical to understand researchers’ perceived knowledge, skills, and opportunities to conduct equity-oriented D&I research.

**Methods:**

We conducted a quantitative inquiry of health researcher’s perspectives using a brief online survey that was distributed during the Annual Dissemination and Implementation Science workshops and through social media and email, after obtaining IRB approval from Washington University of St. Louis.

**Findings:**

We received 180 survey responses over three months of data collection. Most respondents were women (81.7%), white (66.1%), academics (78.9%), and faculty members (53.9%). Many participants reported they were Advanced (36.7%) or Advanced Beginners (27.8%) in the D&I field. When queried about their motivations, respondents indicated high agreement regarding their interest in applying theories, models, and frameworks for promoting health equity in D&I research (91.7%); identifying implementation strategies to promote equity-oriented D&I research (91.1%); and defining and operationalizing equity-oriented D&I research (95.6%). In contrast, respondents reported higher levels of disagreement on having the skills necessary to conduct contextual assessments (33.9%) or information needed to define, operationalize, or measure health equity in D&I research (43.9%). When asked about factors that could influence their ability to incorporate health equity into D&I research, 44.4% reported not having the skills necessary to conduct this type of research, 32.2% stated difficulties in receiving funding for this type of research, and 30% stated that it was challenging to find appropriate collaborators to engage in health equity-oriented D&I research.

**Implications for D&I Research:**

In light of limited empirical work in this area, these study findings provide important initial in-depth empirical insights into health researcher’s perceptions of capacity for proposing and conducting equity-oriented D&I research. Collectively, these data highlight key targets that can directly inform the development of training opportunities for conducting equity-oriented D&I research for researchers that represent a wide range of D&I and health equity-related experience.

### S20 Conceptualizing and measuring public opinion as outer-setting in the emerging area of policy-focused implementation science

#### Jonathan Purtle

##### Health Management &Policy, Drexel University School of Public Health, Philadelphia, PA, USA

###### **Correspondence:** Jonathan Purtle (jpp46@drexel.edu)

**Background:** Policy-focused implementation science is an emerging area. With this growth, an increasing number of policy-focused implementation researchers are struggling to conceptualize and measure domains of constructs that were initially developed for clinically-focused implementation research questions. “Outer-setting” is one such domain of constructs. For policy-focused implementation researchers, confusion stems from policy typically being considered an outer-setting factor that is distal to the evidence-based practice that is central to the research question. Thus, what constitutes outer-setting when adoption of an evidence-based policy is central to the research question? Political science research suggests that public opinion is a major determinant of policy adoption that should be conceptualized as an important outer-setting factor in policy-focused implementation science. This presentation will detail methodological approaches for measuring public opinion in policy-focused implementation science research.

**Methods:** Narrative review of approaches used in political science research to conceptualize and measure public opinion about public policies.

**Findings:** Three primary methodological approaches were identified. First, public opinion surveys can produce region-specific estimates of prevalence and correlates of support for an evidence-based policy and beliefs about the issue that the policy addresses. Relatedly, public opinion survey experiments—in which respondents are randomized to read messages before answering questions—can inform the development of dissemination messages that cultivate public support for evidence-based policies, which can subsequently influence policy adoption. Second, social media content analysis can generate similar insights about public opinion related to an evidence-based policy and the issue it addresses. Natural language processing methods can be used to efficiently identify themes and cluster analysis techniques can be applied to identify discrete audience segments and constituent groups that could affect policy adoption. Third, news media content analysis can elucidate how causes, consequences, and solutions related to policy issues are portrayed in public discourse. These media portrayals can be conceptualized as influencing, and also being influenced by, public opinion and regionally linked to data from public opinion surveys and social media content analyses.

**Implications for D&I Research:** Opportunities exist to integrate public opinion research methodologies into policy-focused implementation science. Such integration can improve the conceptualization and measurement of outer-setting factors surrounding the adoption of evidence-based policies.

**Primary Funding Source:** National Institutes of Health

### S21 Using machine learning and an implementation research logic model to facilitate the evaluation of a quality improvement program

#### Jiancheng Ye^1^, Jennifer Bannon^2^, Justin Smith^1^, Abel Kho^3^, Theresa Walunas^3^

##### ^1^Northwestern University Feinberg School of Medicine, Chicago, IL, USA; ^2^Northwestern University, Chicago, IL, USA; ^3^Northwestern University, Feinberg School of Medicine, Chicago, IL, USA

###### **Correspondence:** Jiancheng Ye (jiancheng.ye@u.northwestern.edu)

**Background:** The digitalization of healthcare systems has resulted in a deluge of big data and prompted the rapid growth of data science in medicine. Machine learning (ML)—a field of study dedicated to the principled extraction of knowledge from complex data—can also benefit implementation science, quality improvement (QI), and primary care research. Despite the increasing number of studies and publications in healthcare, there have been few examples of combining ML and implementation models/frameworks to evaluate practice facilitation-supported QI programs in primary care.

**Methods:** We applied ML algorithms to data from Healthy Hearts in the Heartland (H3), including practice facilitation data, practice and staff participation survey data, to assess the relationship between practice attributes and practice facilitator strategies and their impact on successful implementation of QI interventions. ML was used to impute missing data, select important features from a large number of variables, identify patterns in the correlations between variables, and infer the underlying latent variables. We also incorporated practice facilitators’ knowledge into the feature selection to include expert perspectives in the results.

**Findings:** We selected 21 features (e.g., using clinical guidelines, reporting QI measures, practice size, etc.) and mapped them onto the five domains (Intervention characteristics, outer setting, inner setting, characteristics of individuals, and implementation process) of the Consolidated Framework for Implementation Research (CFIR). Cronbach’s alphas of the five domains are 0.71, 0.86, 0.82, 0.89, 0.72, separately. We used structural equation modeling to analyze the relationships among features, latent variables, practice facilitation strategies (Doing Tasks, Project Management, Consulting, Teaching, and Coaching), and outcomes (implemented QI interventions). Some latent factors, such as inner setting, have impact on “Doing task” strategies (*P*=0.02). All the five facilitation strategies have statistically significant associations with the implementation outcome (all *P*<0.001).

**Implications for D&I Research:** ML can be used to investigate the relationships between data elements in large and complex datasets which may unearth insights that can help to improve practice facilitation-driven QI in primary care and other multicomponent implementation strategies. The combination of ML and the theory behind the Implementation Research Logic Model (IRLM) holds promise for improving implementation strategy evaluation.

**Primary Funding Source:** Agency for Healthcare Research and Quality

### S22 Combining the brownson capacity building model and the translational science benefits model to inform the evaluation of the UC San Diego actri dissemination and implementation science center

#### Clare Viglione, Borsika Rabin, Jamie Lee Pilapil, Gregory Aarons, Lauren Brookman-Frazee, Nicole Stadnick

##### UC San Diego ACTRI Dissemination and Implementation Science Center, La Jolla, CA, USA

###### **Correspondence:** Clare Viglione (cviglione@health.ucsd.edu)

**Background:**

The UC San Diego Altman Clinical and Translational Research Institute Dissemination and Implementation Science Center (DISC) launched in 2020 to establish a flagship for dissemination and implementation science (DIS) through training, technical assistance, community engagement, and research advancement. To prioritize DIS capacity building, the DISC developed a program-wide logic model integrating domains from the Translational Science Benefits Model (TSBM) to inform the evaluation of member engagement and impact related to DISC services.

**Methods:**

The DISC Logic Model (DLM) was developed by combining the Brownson Capacity Building model with TSBM domains to link capacity building and scientific activities with outputs and downstream public benefit. The DLM served as the framework for a 37-item survey capturing quantitative and qualitative information about Scientific Activities, Outputs, and Impact. The survey was distributed to 335 DISC Members via electronic newsletter with two reminders, one Twitter post, and an incentive of a $25 raffle.

**Findings:**

The final DLM included four Inputs (i.e., Financial, Human, Infrastructural, and Knowledge), seven Scientific Activities (e.g., DIS Professional Networking, DIS Grant Development), 20 Scientific Outputs (e.g., number of grants submitted and awarded), and several areas of Impact organized into the TSBM Societal Benefits domains. Regarding inputs, 114 (34%) responded to the survey including 55% DISC Members and 30% DISC Investigators. 98% reported participating in at least one Scientific Activity with the following as the most popular: Monthly Journal Club (47%), Annual DIS Methods Workshop (39%), and DISC Consultation (30%). With respect to outputs and impact, 56% endorsed at least one of the following as a result of DISC engagement: grant preparation, submission, or receipt; paper submission; developing a new scientific collaborator or community partnership; operationalizing a program; and presenting at a scientific or community conference.

**Implications for D&I Research:**

The DLM facilitated a comprehensive evaluation of our center. Engagement in the DISC is high with nearly all members participating in at least one activity. Technical assistance offerings such as Journal Club and Consultation were most popular. Actionable steps include prioritizing technical assistance and identifying streamlined approaches to facilitate DIS grant writing through targeted writing workshops, “office hours” or Organized Writing Leagues.

**Primary Funding Source:** UC San Diego Health Sciences

### S23 Advancing review criteria for dissemination and implementation science grant proposals

#### Nicole Stadnick^1,2,3^, Clare Viglione^1^, Erika Crable^1,3,4^, Jessica Montoya^1,2^, Sarah Linke^1,2^, Maryam Gholami^1,2^, Irene Su^1,2^, Borsika Rabin^1,4^

##### ^1^UC San Diego ACTRI Dissemination and Implementation Science Center, La Jolla, CA, USA; ^2^University of California San Diego, La Jolla, CA, USA; ^3^Child and Adolescent Services Research Center, San Diego, CA, USA; ^4^University of California, San Diego, La Jolla, CA, USA

###### **Correspondence:** Nicole Stadnick (nstadnic@health.ucsd.edu)

**Background:**

Existing grant review criteria do not consider unique methods and priorities of Dissemination and Implementation Science (DIS). The ImplemeNtation and Improvement Science Proposals Evaluation CriTeria (INSPECT) scoring system includes 10 criteria based on the Proctor et al. “ten key ingredients” and was developed to support assessment of DIS research proposals. We describe how we adapted INSPECT and used it in combination with the NIH scoring system to evaluate pilot D&I proposals through our DIS Center.

**Methods:**

We adapted the INSPECT criteria to broaden considerations for DIS settings and concepts. Five PhD-level researchers with intermediate to advanced DIS knowledge were trained to conduct reviews of seven pilot grants using INSPECT and NIH criteria. INSPECT overall scores range from 0-30 (higher scores are better) and NIH overall scores range from 1-9 (lower scores are better). Each grant was independently reviewed by two reviewers followed by a group meeting to identify experiences using both criteria and to finalize decisions about the five awardees. A follow-up survey was sent to reviewers to expand on reflections using each criteria.

**Findings:**

Averaged across reviewers, NIH overall scores ranged from 2-5 while INSPECT overall scores ranged from 13-24. Reflections from reviewers highlighted unique value and utility for each scoring criteria. NIH criteria had a broad scientific purview and were better suited to evaluate more effectiveness-focused and pre-implementation proposals with less formed implementation strategies. The INSPECT criteria were easier to rate in terms of the quality of integrating DIS considerations into the proposal and to assess potential for generalizability, real world feasibility and impact. Overall, reviewers noted that INSPECT was a helpful tool to guide DIS research proposal writing.

**Implications for D&I Research:**

We confirmed complementarity in using both scoring criteria in our pilot grant proposal review and highlighted the utility of INSPECT as a potential DIS resource for training and capacity building. Possible refinements to INSPECT include more explicit reviewer guidance on assessing pre-implementation proposals, inviting reviewer commentary on specific ratings, and greater clarity on rating criteria with overlapping descriptions. Next steps are to evaluate inter-rater reliability and qualitative reviewer reflections to refine the scoring system for our next pilot application cycle.

### S24 The state of D&I sciences in 2020-2021

#### Jonathan Tobin

##### Clinical Directors Network, Inc. (CDN), New York, NY, USA

###### **Correspondence:** Jonathan Tobin (jntobin@cdnetwork.org)

**Background:**

This presentation provides definitional and conceptual foundations for discussing D&I research and science. Key messages extracted from the *Journal of Clinical and Translational Science* Special Issue on D&I science (2020) and other *JCTS* articles set the stage by describing the current state of D&I sciences through the collective perspectives of these *JCTS* authors and editors. The overview summarizes this representative selection of leading scientific work and points to key areas for developing scientific consensus to advance the field, as well as emerging topics worthy of our attention.

**Methods:**

Manuscripts in the *JCTS* special issue on D&I Research^1^ described (1) innovative strategies and frameworks designed to enhance and improve the translation of research to practice; (2) opinions of D&I researchers and CTSA leaders as to which strategies to could promote team science and improve population health; (3) partnerships between academic and public health systems; (4) stakeholder engagement; (5) workforce development.

**Findings:**

Several emerging areas for research were identified: (1) the role of practice facilitation; (2) neglected stakeholders (3) monitoring study quality/maintaining fidelity; (4) reporting, including return of results and the changing role of data and safety monitoring boards (DSMBs) and monitoring systems-level implementation outcomes. Key ongoing challenges for D&I research include the development of full-spectrum translational research teams that engage stakeholders from across the research continuum, by bringing basic science investigators together with practicing clinicians, patients, caregivers, and other stakeholders.

**Implications for D&I Research:**

This raises the question: How can team science facilitate learning healthcare systems that improve clinical and population health? The creation of learning healthcare systems will depend on leveraging clinical informatics and training the workforce to use this information, both at the point of care and for monitoring at the population health level for continuous feedback. Only then can we truly translate research into practice for the enhancement of health equity.

**Reference:**

Stevens, K.R. and Tobin, J.N., 2020. Introduction to the JCTS special issue on Dissemination and Implementation Sciences. *Journal of Clinical and Translational Science*, *4*(3), pp.149-151.

**Primary Funding Source:** National Institutes of Health

### S25 Benefits of d&I in every stage of translational science

#### Dr. Aaron Leppin^1^, Jane Mahoney^2^

##### ^1^Mayo Clinic, Rochester, MN, USA; ^2^Department of Medicine, University of Wisconsin-Madison, Madison, WI, USA

###### **Correspondence:** Jane Mahoney (jm2@medicine.wisc.edu)

**Background:**

Translation of research knowledge into routine practice requires dissemination and implementation (D&I), yet D&I is often not central to the goals and activities of CTSA hubs. Further, the sciences that inform the processes of D&I are not well described regarding their value in improving translation. Embedding D&I into the identity of CTSAs will require a clearer conceptualization of how these sciences relate and articulation of recommendations based on the implications. In two manuscripts, we—members of the Advancing D&I in CTSAs Working Group—address these challenges.

**Methods:**

We convened a writing group of experts from multiple CTSAs with different types of translational research. We reviewed existing conceptual definitions, frameworks, and examples. We then sought to explain the relationship between D&I sciences and translational science and show how D&I can support research translation at all stages^1^. For the second paper, we convened a separate group of experts with experience in different areas of translational science infrastructure (workforce development, community engagement, methods, processes, evaluation). This group met regularly to discuss implications of the first body of work and develop consensus recommendations for advancing D&I in CTSAs^2^.

**Findings:**

We developed the Integrative Framework of Dissemination, Implementation, and Translation (IFDIT), conceptually distinguished the processes of research conduct and application, and showed with tables and examples how this work can be interpreted and applied. In our second paper, we described implications of this work and made recommendations for NCATs and CTSA leaders in the areas of workforce development, methods and processes, and evaluation.

**Implications for D&I Research:**

This body of work provides conceptual rationale and practical steps for advancing D&I sciences in CTSAs to hasten translation and impact.

**References**

1. Leppin, A. L., Mahoney, J. E., Stevens, K. R., et al., (2020). Situating dissemination and implementation sciences within and across the translational research spectrum. *JCTS*, *4*(3), 152-158.

2. Mehta, T., Mahoney, J., Leppin, A. L., Stevens, K. R., Yousefi-Nooraie, R., Pollock, B. H., ... & Moore, J. B. (2021). Integrating dissemination and implementation sciences within Clinical and Translational Science Award Programs to advance translational research: Recommendations to national and local leaders. *JCTS*, 1-22.

**Primary Funding Source:** National Institutes of Health

### S26 Building shared d&I competencies across clinician/practitioners, community leaders, and researchers

#### Jane Mahoney^1^, Kathleen Stevens^2^

##### ^1^Department of Medicine, University of Wisconsin-Madison, Madison, WI, USA; ^2^University of Texas Health San Antonio, San Antonio, TX, USA

###### **Correspondence:** Kathleen Stevens (stevensk@uthscsa.edu)

**Background:**

Investment in scientific workforce can close gaps in D&I research across CTSAs. The members of the Advancing D&I in CTSAs Working Group convened two working groups to develop recommendations for training the scientific and practitioner workforces in D&I competencies.

**Methods:**

The Group identified gaps in existing D&I training programs. IFDIT^1^ was used to identify target groups for training in D&I sciences, identify D&I principles that apply across all translational sciences, and develop exemplar competencies related to those principles. The Group addressed how training could be modified to fit type of translational research, learner stage, and type of workforce.

**Findings:**

We conceptualize four fundamental D&I science principles that apply across all translational stages and provide examples of competencies that maximize design for ultimate translation from one stage to the next. The TFI^2^ framework articulates that all people who carry out implementation related tasks are poised to benefit from training, which should be tailored to the context and goals of the learner, understanding that competencies differ depending on the setting of implementation practice. The TFI framework proposes a common curricular foundation, identifies mentoring as critical to meeting learners’ needs, and recommends maximizing co-learning opportunities for researchers and practitioners. Novel D&I training programs in CTSAs provide examples of tailoring curriculum to meet workforce development needs. The Learning Health System affords a new model that illustrates multi-directional translational research and quality improvement partnership between health system operations and scientists.

Effective D&I training requires tailoring to the stage of translation (T1 through T4), learner levels, and workforce role (researcher, consultant, and practitioner).

**Implications for D&I Research:**

Further work is needed to train the scientific and practitioner workforce in D&I competencies that will maximize translational research and its application to practice. These recommendations provide a roadmap to integrate D&I training into workforce development in CTSAs.

**References**

1. Leppin, A. L., et al. (2020). Situating dissemination and implementation sciences within and across the translational research spectrum. *JCTS*, *4*(3), 152-158.

2. Leppin, A. L., et al. (2021). Teaching for Implementation: A framework for building implementation research and practice capacity within the translational science workforce. *Journal of Clinical and Translational Science*, 1-26.

**Primary Funding Source:** National Institutes of Health

### S27 Bridging translational and d&I research: The future of d&I research in the CTSA landscape

#### Andrew Quanbeck

##### University of Wisconsin, Madison, WI, USA

###### **Correspondence:** Andrew Quanbeck (andrew.quanbeck@fammed.wisc.edu)

**Background:**

D&I research has an important conceptual place in CTSA-funded translational science institutes, given that D&I is integral to the NCATS translational science model. Despite this, D&I research is frequently unrepresented or underrepresented at many CTSIs. While the disciplines of translational and D&I research share overlapping goals, there appears to be relatively little academic crossover between disciplines, presenting an opportunity for greater integration in the future.

This discussion begins by offering a briefing on past and current NIH/NCATS D&I Working Group activities. A history of the first D&I Working Group points to the objectives achieved. It is followed by a discussion of the newly approved NCATS D&I working group, outlines the future, and invites participants to join the effort.

**Methods:**

To illustrate conceptual gaps between implementation and translational science and to highlight opportunities for future collaboration between disciplines, we will present: 1). An overview of the most cited publications within the journal *Implementation Science* from 2006-2019, including a directed citation network with findings illustrated as a “heat map”; and 2). Comparison of the implementation research directed citation network to a translational research directed citation network published by Fort et al., 2017.

**Findings:**

Findings illustrate a striking lack of cross-referencing that has occurred between the most influential papers published in the domains of translational and implementation research since 2006. We examine the coevolution of the two fields and possible causes of the academic divergence that has occurred (including differing origins, structures of funding, timelines, and geography). Finally, we highlight themes that may be helpful to researchers in bridging the two disciplines.

**Implications for D&I Research:**

When academic disciplines develop in isolation there is less opportunity for researchers to inform each other's work, and it can take longer to implement relevant theories, methods, and practices in the communities that CTSAs serve.

The discussion will advocate for approaches from systems engineering and team science to facilitate the bridging process. Increased collaboration across fields may reduce redundancies, disseminate lessons learned more quickly, and subsequently improve the fields of translational and implementation science to assist in bringing research into practice more quickly, efficiently, and equitably.

**Primary Funding Source:** National Institutes of Health

## Clinical Care Settings: Patient-level Interventions

### S28 Implementation strategy bundling to support positive communication for long-term care residents living with dementia

#### Natalie Douglas

##### Central Michigan University, Mt. Pleasant, MI, USA

###### **Correspondence:** Natalie Douglas (natalie.douglas@cmich.edu)

**Background**: Communicating with residents living with dementia in long-term care settings is challenging and can cause significant burden on care partners, especially certified nursing assistants who provide the most direct care.^1^ Traditional staff training programs have limited evidence to support staff learning outcomes in some cases.^2^

**Methods**: An implementation bundle was designed to increase adoption of a collaborative coaching program among residents with dementia, speech-language pathologists, and certified nursing assistants. This program was shown to improve communication interactions in a pilot study.^3^ The CFIR-ERIC Implementation Strategy Matching Tool ^4^ was used to match implementation strategies to documented barriers to implementation in the areas of knowledge and beliefs about the intervention, self-efficacy, individual stage of change, and individual identification with organization.

**Findings**: The implementation strategy bundle consisted of a booklet of operationalized items related to identifying and preparing champions and early adopters, building a coalition, informing local opinion leaders, promoting network weaving, and capturing and sharing local knowledge. Preliminary data regarding the scale-up of the collaborative coaching program via the implementation bundle in partnership with a national rehabilitation company in 6 skilled nursing facilities will also be provided.

**Implications for D&I Research:** While implementation strategies are often used, the logistics of matching and operationalizing strategies to documented barriers is often vague. This presentation will present the process of operationalizing implementation strategies as part of intervention delivery which may support the uptake of non-pharmacological interventions in a complex environment such as long-term care.

**Primary Funding Source:** American Speech-Language-Hearing Foundation

### S29 Communicating communication preferences: Provider perspectives on implementing a person-centered communication intervention

#### Miranda Corpora^1^, Megan Kelley^1^, Alexandra Heppner^2^, Kelly Knollman-Porter^1^, Vanessa Burshnic-Neal^3^, Kimberly Van Haitsma^4^, Katy Abbott^1,2^

##### ^1^Miami University, Oxford, OH, USA; ^2^Scripps Gerontology Center at Miami University, Oxford, OH, USA; ^3^Tampa, FL, USA; ^4^The Pennsylvania State University, University Park, PA, USA

###### **Correspondence:** Miranda Corpora (corpormr@miamioh.edu)

**Background:** The *Preferences for Everyday Living Inventory* (PELI) is an evidenced-based, validated instrument used to enhance the delivery of person-centered care. The Preferences for Activity and Leisure (PAL) Cards were developed to communicate important resident preferences across care team members and offered as a Quality Improvement Project. The purpose of this study was to replicate PAL Card implementation and test its newest feature, communication preference icons. Preferences included using gesture to present key words, use of glasses and hearing aids, need for increased volume, and need for visual and written cues.

**Methods:** Providers were recruited to create PAL cards for 15-20 residents. Provider champions participated in (n=57) monthly semi-structured telephone interviews that were recorded, transcribed verbatim, and checked for accuracy before thematic analysis. The interviews provided insight into the barriers and facilitators to implementing PAL Cards, as well as feedback on the new communication preference icons.

**Findings:** Participating providers (N=16) were from not-for-profit 50% (*n=8*), for-profit 38% (*n=7*), and private pay 13% (*n=2*) communities. Providers attempted 154 PELI interviews with residents and completed 147 (95%). Providers attempted to create 147 PAL cards and completed 126 (85%). On average, it took providers 15 minutes (SD = 7 minutes) to create PAL Cards. While many providers commented on the benefit of using the icons and a desire to keep using the icons, some expressed lack of clarity in interpreting the icons and concern for violating HIPAA privacy.

**Implications for D&I Research:** Communication icons on PAL Cards provide direct care workers with a quick and easy way to implement residents’ communication preferences into care delivery, which is of particular importance for nursing homes who often have high provider turnover and a resident population with high incidence of communication challenges.

Communicating communication preferences along with activity and leisure preferences may help lead to more meaningful relationships between staff and residents, higher resident satisfaction with care, and higher job satisfaction among providers.

**Primary Funding Source:** Ohio Department of Medicaid

### S30 Measuring outcomes of home-delivered meal recipients: Feasibility of implementing the interrai home care frailty scale

#### Lisa Juckett

##### The Ohio State University, Columbus, OH, USA

###### **Correspondence:** Lisa Juckett (lisa.juckett@osumc.edu)

**Background:** Each year, over 2.4 million older adults across the United States receive home-delivered meals (HDMs). HDM recipients typically present with multiple chronic medical conditions, require three or more daily medications, and are unable to independently complete routine activities. These health characteristics place HDM recipients at elevated risk for frailty—a complex condition associated with health decline and hospitalizations. Though HDM staff (e.g., assessors) are well-positioned to implement frailty outcome measures and monitor changes in recipients’ frailty levels, little is known about the measurement of frailty in HDM settings. As such, this study assessed the feasibility of implementing the interRAI Home Care Frailty Scale (HCFS) with HDM recipients over a 6-month period.

**Methods:** An interrupted time series design was used to examine HDM staff’s implementation of the HCFS between June 2020 – December 2020 in one HDM organization in the Midwest United States. Seventeen staff interviews were conducted to explore barriers to outcome measure implementation. Directed content analysis identified major implementation barriers (e.g., few available resources; lack of communication) as guided by the Consolidated Framework for Implementation Research. To overcome these barriers, five implementation strategies were deployed over 6-months: conduct ongoing training, appoint a frailty champion, complete pilot testing, change record systems, and conduct chart audits. Retrospective chart reviews determined rates of HCFS implementation.

**Findings:** Implementation rates of the HCFS ranged from 69.8% - 94.6%. Rates were highest in Month 1 (94.6%) immediately following initial training sessions and the establishment of an electronic record system that facilitated ease of HCFS documentation. Chart audits were discontinued in Month 4, contributing to the decline in implementation rates in Month 5 (69.8%). Implementation increased to 86.0% following the reinstatement of chart audits and feedback provided to HDM staff by the internal frailty champion.

**Implications for D&I Research:** Barriers to outcome measure implementation should be assessed to inform selection of implementation strategies for the community-based context, such as HDM settings. While initial training sessions supported outcome measure implementation, trainings must be supplemented by additional implementation strategies, including the completion of chart audits and engagement of an internal champion to optimize outcome measure use with frail, HDM recipients.

**Primary Funding Source:** Administration for Community Living

### S31 Facilitating aging in place and community by empowering participation in society: A mixed methods study on the co-development of a theory of change with suburban-dwelling older adults

#### Emily Balog

##### Washington, DC, USA; Rutgers University, Newark, NJ, USA

###### **Correspondence:** Emily Balog (emily.balog@rutgers.edu)

**Background**: In suburban communities today, older adults make up 51% of the population. Ninety-five percent of all older adults live in community settings and have expressed a desire to avoid costly institutionalization and to remain in their homes and communities *(aging in place)*. Communities have recognized the need for action planning to support the needs of older adults to age in place; however, plans often lack empirical evidence, they leave out older adults, particularly those with disabilities, and the planning process takes a top-down approach often from the perspective of planners and policy makers.

**Methods**: The goal of this mixed methods study was to understand fidelitous empowerment procedures of older adult participation in society through person-centered community action planning. Older adults from three suburban communities were telephonically surveyed (n=64) and interviewed (n=14).

**Findings**: Three variables, functional mobility (WHODAS score; B= -0.266), availability of healthcare services (B= 8.20), and availability of information to events, services, and programs (B= 8.905) added statistically significantly to the prediction (*p* < .05) of the dependent variable satisfaction with participation. Qualitative findings informed a theory of change which articulates a set of needs that are desired and suggested objectives to address these needs. Stakeholders identified four fidelitous empowerment procedures required to induce person-centered community action planning: leveled engagement, communication, enfranchisement and champions, and social/cultural capital.

**Implications for D&I Research:** Within the context of the Consolidated Framework for Implementation Research (CFIR), domains related to the *individuals involved*, the *inner setting*, and the *outer setting* must first be understood as needs, suggested objectives to meet needs, and desired outcomes so that *interventions* (theory of action) can be designed with the explicit theory of change *process*. The theory of change developed in this study explains that older adults are empowered to participate in one’s community when there is support for basic and home living needs (basic), when options for participation match one’s level of ability, interest, and values (social), and when one is invited and knows where to find information about programs, services, or events (growth). Planning teams now have the translational tools to create customizable, theory-driven, and evidence-based strategies to engage diverse older adults.

### S32 Optimizing dissemination and implementation of pediatric obesity treatment programs: Lessons from bright bodies

#### Emily B. Finn^1^, Marissa Gowey^2^, Caroline Keller^2^, Mary Savoye^1^, Stephanie Samuels^3^, Abby Fleisch^4^, Victoria Rogers^5^, Margaret Grey^3^, Laura Damschroder^6^, Amy Beck^7^, Mona Sharifi^8^

##### ^1^Yale School of Medicine, New Haven, CT, USA; ^2^University of Alabama at Birmingham, Birmingham, AL, USA; ^3^Yale University, New Haven, CT, USA; ^4^Maine Medical Center, Portland, ME, USA; ^5^American Academy of Pediatrics, Itasca, IL, USA; ^6^Implementation Pathways, LLC, Ann Arbor, MI, USA; ^7^University of California San Francisco, San Francisco, CA, USA; ^8^Yale University School of Medicine, New Haven, CT, USA

###### **Correspondence:** Emily B. Finn (e.finn@yale.edu)

**Background:**

With one in three children in the U.S. affected by overweight/obesity and disparities exacerbated by COVID-19, there is urgent need for increased availability of comprehensive, evidence-based interventions. Bright Bodies (BB) is a multi-disciplinary, family-based, intensive pediatric obesity treatment program developed and proven effective in an urban population from diverse racial/ethnic backgrounds. While BB has been disseminated to nearly 40 sites globally over the last two decades, little is known about implementation at these heterogeneous sites in clinical, community, and school-based settings. We conducted a mixed-methods formative evaluation with dissemination sites to optimize the intervention and implementation package.

**Methods:**

We invited key personnel from U.S-based dissemination sites to participate in a survey and semi-structured interview exploring relevant constructs from the Consolidated Framework for Implementation Research and the Organizational Readiness to Change Assessment. Using the constant comparative method, investigators independently coded interview transcripts and achieved consensus through iterative discussions. We used a merging analysis approach to integrate descriptive analyses of surveys with qualitative findings.

**Findings:**

Of 30 sites invited, contacts from 16 (53.3%) completed surveys and seven (23%) interviews. Participants represented multiple disciplines (nursing, nutrition, behavioral/mental health, etc.) and settings (academic practices, community health centers, schools, etc.). Of 14 sites with participants involved in the acquisition/use of the BB curriculum, 11 (79%) used it in a new program or adapted an existing program. Of those, four (36%) still use BB while six (55%) discontinued BB for reasons other than COVID-19. High quality of evidence and demand for obesity programming were facilitators of acquisition. Several barriers negatively impacted adoption, implementation, and sustainability, including: (1) funding, the most common barrier cited across sites; (2) dissemination site diversity that required considerable adaptation (e.g., staffing, delivery modes); and (3) insufficient training and implementation facilitation, which have been optional and variable.

**Implications for D&I Research:**

The findings suggest insights into opportunities for implementation package enhancements, including: (1) support for dissemination sites in work across sectors, including payers, to develop sustainable funding models; (2) specific guidance about core functions vs. adaptable forms to help sites balance flexibility and fidelity; and, (3) standardized training and ongoing support within the implementation package.

**Primary Funding Source:** National Institutes of Health

### S33 Applying the RE-AIM framework to guide a process evaluation of telewound implementation in the veterans health administration

#### Marissa Wirth^1^, Bella Etingen^1^, Jamie Patrianakos^1^, Bridget Smith^1^, Timothy Hogan^2^, Kevin Stroupe^1^, Elizabeth Tarlov^1^, Salva Balbale^1^, Rebecca Kartje^1^, Carol Kostovich^1^, Frances Weaver^1^

##### ^1^Center of Innovation for Complex Chronic Healthcare (CINCCH), Edward Hines Jr. VA Hospital, Veterans Health Administration, Hines, IL, USA; ^2^Edith Nourse Rogers Memorial Veterans Hospital, Veterans Health Administration, Hines, IL, USA

###### **Correspondence:** Marissa Wirth (Marissa.Wirth@va.gov)

**Background:**

The Veterans Health Administration (VHA) began a roll-out of Telewound Practice (TWP) in 2019. TWP was designed to reduce Veteran travel burden and cost related to wound care by utilizing telehealth. The VHA Diffusion of Excellence office partnered with VHA researchers to conduct a mixed-methods program evaluation assessing barriers and facilitators to implementation and associated outcomes.

**Methods:**

TWP implementation evaluation was guided by the Reach, Effectiveness, Adoption, Implementation and Maintenance (RE-AIM) framework. Evaluation data for four implementation pilot sites were collected using VHA administrative databases and surveys conducted with Veterans and wound care team members (WCTM). In VHA databases, TWP visits were identified using a TWP specific clinic code. Survey responses on 5-point Likert scale were combined to strongly agree/agree and strongly disagree/disagree/neutral. Data were analyzed using descriptive statistics.

**Findings:**

*Reach*. After TWP implementation 655 Veterans at the 4 pilot sites received telehealth for wound care. *Effectiveness*. Most reported TWP aided their understanding (84%;140/167) and motivation (70%;112/160) for wound management. WCTM survey respondents indicated TWP improved (80%;12/15) Veterans access to wound care, (60%;9/15) decision-making related to wound care, and (73%;11/15) overall quality of care for Veterans. Both (85%;132/156) Veterans and (80%;12/15) WCTMs indicated that TWP reduced Veteran travel costs. *Adoption*. Four of the five original pilot sites participated in the implementation of the program, two of those four provided TWP to Veterans. *Implementation*. 67%(10/15) of WCTMs indicated they received adequate training to confidently provide TWP services, and (53%;8/15) indicated TWP was easy to use. However, around half (53%;8/15) indicated TWP increased their daily workload and (50%;5/10) time spent on clinical documentation/paperwork. *Maintenance*: The number of Veterans who received TWP care increased over time following implementation.

**Implications for D&I Research:**

Veterans who received TWP were highly satisfied, felt TWP improved their ability to manage their wounds, and were interested in continuing to receive TWP care. Likewise, WCTMs perceived TWP to be beneficial for Veterans and easy to integrate. However, half of the initial TWP pilot sites were unable to successfully implement TWP. Future efforts to implement TWP should account for these barriers from the outset.

**Primary Funding Source:** Department of Veterans Affairs

### S34 A community health worker delivered intervention to address hypertension among adults engaged in HIV care in northern Tanzania: Outcomes from a hybrid type 1 effectiveness-implementation pilot study

#### Preeti Manavalan^1,2,3^, Deng Madut^3,4^, Lisa Wanda^5^, Ally Msasu^5^, Blandina Mmbaga^5,6,7,8^, Nathan Thielman^3,4^, Melissa Watt^3,9^

##### ^1^University of Florida, Gainesville, FL, USA; ^2^University of Florida, Durham, NC, USA; ^3^Duke Global Health Institute, Durham, NC, USA; ^4^Duke University, Durham, NC, USA; ^5^Kilimanjaro Clinical Research Institute, Moshi, Tanzania, United Republic of; ^6^Kilimanjaro Christian Medical University College, Moshi, Tanzania, United Republic of; ^7^Duke Global Health Institute, Moshi, Tanzania, United Republic of; ^8^Kilimanjaro Christian Medical Center, Moshi, Tanzania, United Republic of; ^9^University of Utah, Salt Lake City, UT, USA

###### **Correspondence:** Preeti Manavalan (preeti.manavalan@medicine.ufl.edu)

**Background:**

The burden of hypertension is increasing among persons with HIV (PWH) in sub-Saharan Africa; yet current care models are inadequate to address this epidemic.

**Methods:**

Utilizing a health belief framework, we developed a patient-directed educational intervention delivered by a community health worker (CHW) and integrated into existing HIV care to address hypertension. The intervention was piloted among hypertensive PWH in an HIV clinic in northern Tanzania over 4 weeks. Intervention sessions included three in-person clinic visits and two telephone calls. A detailed educational curriculum was created for each session. Blood pressure was measured at all in-person sessions and the 2-week post-intervention assessment. We used a hybrid type 1 approach and conducted a pilot study to examine implementation outcomes and potential clinical impacts of the intervention. Key implementation outcomes were feasibility, fidelity and acceptability of the intervention and included the proportion of participants retained in the intervention and those referred to a prescribing provider for additional hypertension care. Clinical outcomes included changes in hypertension care engagement and systolic and diastolic blood pressure (SBP and DBP).

**Findings:**

Among 16 eligible participants, 14 (64% women, median age 54.5, IQR 46.0–62.0, years) were recruited into the study, and 13 (92.9%) were retained throughout all intervention sessions. The intervention was delivered with 98.8% fidelity to curriculum content. All participants reported they found it helpful to meet the CHW in-person and speak with them on the phone. Prior to the intervention, 2 (15.4%) participants had seen a doctor previously for hypertension, compared to 11 (84.6%) participants post-intervention (p=0.0027). No participant was using antihypertensives pre-intervention, compared to 10 (76.9%) post-intervention (p=0.0016). Pre-intervention median SBP was 164 (IQR 152–170) mmHg, compared to post-intervention SBP of 146 (IQR 134–154) mmHg (p=0.0029). Pre-intervention median DBP was 102 (IQR 86–109) mmHg, compared to post-intervention DBP of 89 (IQR 86–98) mmHg (p=0.0023).

**Implications for D&I Research:**

A patient-directed educational intervention, delivered by a CHW and integrated into existing HIV care, is highly feasible, has the potential to improve hypertension care engagement, and reduce blood pressure. Further testing and scale-up of such interventions are urgently needed among hypertensive PWH in Tanzania and similar settings.

**Primary Funding Source:** National Institutes of Health

### S35 Interactive care coordination and navigation (iCAN) study: End-user feedback for implementation of a mHealth intervention for people experiencing homelessness

#### Leticia Moczygemba^1^, Monika Semwal^1^, James Baffoe^1^, Emily Seales^2^, Chinyere Okoh^1^, Mark Hilbelink^2^, Carolyn Brown^1^

##### ^1^The University of Texas at Austin College of Pharmacy, Austin, TX, USA; ^2^Sunrise Homeless Navigation Center, Austin, TX, USA

###### **Correspondence:** Leticia Moczygemba (lrmoczygemba@austin.utexas.edu)

**Background:** The ability of mobile technology to reach “anyone, anywhere” makes mHealth interventions a potential solution for improving care coordination among people experiencing homelessness (PEH). However, successful implementation hinges upon understanding how mHealth fits into everyday lives of PEH. The study purpose was to obtain end-user feedback and implementation strategies about a Smartphone-based mHealth intervention aimed to improve care coordination and decrease emergency department (ED)/hospital use among PEH.

**Methods:** Four focus groups were conducted in April/May 2021 with 26 PEH at a community navigation center in Austin, Texas. A summary of the mHealth intervention was provided prior to the focus group. Then, participants indicated what they liked/disliked about the intervention and gave suggestions for improvement. Participants also shared what would make it easy/hard to participate in the intervention and strategies for overcoming any barriers. The constant comparison method, whereby similarities/differences within and across groups guided coding of categories, was used for data analysis.

**Findings:** Participants were 45.5±10.7 years old, 84.6% male, 50.0% white and 19.2% black. The mean duration of homelessness was 5.9±7.5 years. Participants had 3.1±1.4 ED/hospital visits in the previous six months. PEH liked many aspects of the text messages including the convenience of being able to connect with people, resources, and health/social services in the community and receive reminders for daily tasks such as taking medications. It was suggested to increase messages about general community information, tailor messages to specific conditions, and add motivational/goal-oriented messages. Participants found the GPS component to be helpful in being able to access resources; few participants expressed privacy concerns. There were concerns about breaking/losing a phone, phone durability, and finding places to charge it. Recommendations for phone safety included keeping phone in pocket, using a phone case/arm band, activating facial security/passcodes, and keeping location services on. Strategies for increasing usability included education/training on how to use the phone, keeping apps simple, and solar-powered batteries.

**Implications for D&I Research:** For mHealth interventions to be effectively translated to PEH, the context of homelessness must be considered. Engaging PEH to give feedback resulted in many practical strategies that can be used to facilitate implementation.

**Primary Funding Source:** Agency for Healthcare Research and Quality

### S36 Adapt or not adapt – lessons learned in culturally adapting an evidence-based decision aid to reduce mammography overuse among older women

#### Jessica Austin^1,2^, Parisa Tehranifar^1^, Gina Wingood^1^, Carmen Rodriguez^1^, Rachel Shelton^3^

##### ^1^Columbia University Mailman School of Public Health, New York, NY, USA; ^2^Mayo Clinic Cancer Center Arizona, Scottsdale, AZ, USA; ^3^United States, Columbia Mailman School of Public Health, New York, NY, USA

###### **Correspondence:** Jessica Austin (Austin.Jessica2@mayo.edu)

**Background:** Enhancing shared decision-making (SDM) is a promising strategy for reducing mammography overuse among older women. A paper-based decision-aid (DA) has shown to be an effective patient-level intervention for enhancing SDM, but few, if any, studies have culturally adapted the DA for use among older, racial/ethnic minority, non-English speaking women. We describe lessons learned culturally adapting the paper-based DA to predominately, older, Hispanic, Spanish-speaking women in New York City.

**Methods:** Using the *Core Phases of the Adaptation Process (Chenel et al., 2018),* we met with 3 stakeholders (researcher, clinician, and patient advocate) to appraise the original DA to identify modifiable content and core components. Next, we conducted semi-structured interviews with 6 Hispanic (66.6% Dominican, 16.7% Mexican, 16.7% Ecuadorian), Spanish-speaking, women (mean age 78) who received a mammogram within the last 12 months (60% marginal/low literacy). Interviews lasted between 60-90 minutes, were audio-recorded, translated and transcribed. Data from stakeholder discussions and older women interviews were combined and analyzed using content analysis.

**Findings:** While potentially useful, all women perceived the content around mammography overuse to be confusing, counter to their beliefs about the importance of mammogram screening, and against their clinicians’ recommendation. Stakeholders were also concerned that the content would induce anxiety rather than empower women to be involved in screening decisions. Findings suggest that culturally adapting the DA may be insufficient or inappropriate for enhancing SDM in this low-literacy population without significant edits to content. Such adaptations may be so extensive that development of a new DA may be warranted, and new modes of delivery (e.g. in-person with clinician) may be useful.

**Implications for D&I Research:** Understanding how interventions and strategies are adapted to fit the cultural and literacy needs of populations is critical for applying implementation science to promote health equity. While several implementation science frameworks include consideration of ‘fit’ prior to implementation, empirical work is needed to assess compatibility of an intervention or strategy. This includes consideration of if adaptation is warranted and the nature of adaptations to make - particularly when the original interventions and strategies are developed and tested in populations with limited diversity and high literacy.

**Primary Funding Source:** Geographic Management of Cancer Health Disparities Program Region 4

### S37 Stakeholder perspectives on implementing an evidence-based behavioral intervention targeting medication adherence and care retention for people living with HIV: A rapid qualitative analysis

#### Amanda Sanchez^1^, Katelin Hoskins^2^, Amy Pettit^3^, Florence Momplaisir^1^, Robert Gross^1^, Kathleen Brady^4^, Carlin Hoffacker^1^, Kelly Zentgraf^1^, Rinad Beidas^1^

##### ^1^University of Pennsylvania, Perelman School of Medicine, Philadelphia, PA, USA; ^2^University of Pennsylvania, Philadelphia, PA, USA; ^3^Independent Consultant, Boston, MA, USA; ^4^Philadelphia Department of Public Health, Philadelphia, PA, USA

###### **Correspondence:** Amanda Sanchez (Amanda.Sanchez1@pennmedicine.upenn.edu)

**Background:** Managed Problem Solving (MAPS) is an evidence-based intervention that can boost HIV medication adherence and increase viral suppression, but it is not widely used in community clinics. Deploying community health workers to deliver MAPS could facilitate broader implementation by adding an additional resource to overburdened clinics. MAPS delivered by CHWs could aid in the Ending the HIV Epidemic (EHE) initiative’s goal of reducing new HIV infections in the US by 90% by 2030.

**Methods:** Semi-structured stakeholder interviews were conducted with stakeholders from 13 Ryan White-funded clinics in Philadelphia, one of 48 US counties prioritized in the EHE. The 4 stakeholder groups included prescribing clinicians, non-prescribing clinical team members (e.g., medical case managers, behavioral health consultants), clinic administrators, and policymakers. Interviews were based on the Consolidated Framework for Implementation Research and investigated perceived barriers to and facilitators of MAPS delivery by community health workers. Rapid qualitative analysis techniques were utilized to efficiently synthesize interview data and identify key categories of determinants along an implementation pathway to serve as inputs for implementation strategy development. Core determinants (i.e., barriers and facilitators) of MAPS implementation were grouped within each category. The determinants derived from the qualitative interviews are the inputs for implementation strategy development for MAPS implementation.

**Findings:** Stakeholders (N=31) were receptive to CHW delivered MAPS and offered critical information on potential implementation determinants including preferences for identification and referral of patients, the importance of integration and communication within the care team, role clarity between staff and the CHW, and the potential of the CHW to improve trust. Outer setting systemic and structural factors also arose as important to consider in MAPS implementation.

**Implications for D&I Research:** This study generated insights regarding barriers and facilitators to implementing an evidence-based behavioral intervention in clinics serving people living with HIV and extends a rapid analysis approach to HIV care, enabling stakeholder data to be incorporated into the development of implementation strategies in real time. This study also offers insights for national implementation efforts associated with EHE.

**Primary Funding Source:** the Penn Center for AIDS Research, Penn Mental Health AIDS Research Center

### S38 Using the theory of planned behavior to improve implementation of mindfulness in people with chronic low back pain

#### Salene Jones^1^, Karen Sherman^2^, Zoe Bermet^2^, Lorella Palazzo^2^

##### ^1^Fred Hutchinson Cancer Research Center, Seattle, WA, USA; ^2^Kaiser Permanente Washington Health Research Institute, Seattle, WA, USA

###### **Correspondence:** Salene Jones (smjones3@fredhutch.org)

**Background:** Chronic low back pain is one of the leading causes of disability across the world. Mindfulness-Based Stress Reduction (MBSR) is recommended by the American College of Physicians as a first line of therapy for chronic low back pain. However, the time commitment and strict structure of MBSR may impede implementation of this evidence-based practice. The Theory of Planned Behavior is a useful framework to conceptualize patient-level determinants of MBSR use such as self-efficacy and attitudes. This study aimed to identify which Theory of Planned Behavior constructs could be a focus of strategies to improve implementation of MBSR.

**Methods:** People with chronic low back pain (n=457) completed an online survey. They read a description of evidence for MBSR and what an MBSR training program involves. They then completed survey items assessing Theory of Planned Behavior constructs: attitudes, norms, self-efficacy, perceived control, intentions to try MBSR training and hours willing to spend learning MBSR. Structural equation modeling was used to assess the association of attitudes, norms, self-efficacy and perceived control with intentions and hours. Procedures were approved by the institutional review board.

**Findings:** Based on preliminary exploratory factor analyses, the self-efficacy and control factors were combined. Self-efficacy/control (0.564), norms (0.245) and attitudes (standardized coefficient: 0.131) were all positively associated with intentions to try mindfulness trainings. Self-efficacy/control (0.408) and norms (0.235) were positively associated with hours a participant was willing to commit to MBSR whereas the association with attitudes (-0.249) was negative. The attitudes factor was highly correlated with norms (0.610) and self-efficacy/control (0.674) and the bivariate correlations between the attitudes items and hours were positive (range: 0.074-0.190), suggesting a possible suppressor effect.

**Implications for D&I Research:** Results suggest self-efficacy/control may be the most strongly related Theory of Planned Behavior construct with intentions to try MBSR. Implementation of MBSR for chronic low back pain should focus on adapting the intervention and improving available resources to overcome logistical barriers. MBSR could be adapted to online formats or drop-in classes to improve adoption for chronic low back pain. Available resources may be needed for childcare, transportation and other logistic challenges to MBSR adoption.

**Primary Funding Source:** National Institutes of Health

## Clinical Care Settings: System-level Interventions

### S39 System determinants of treatment impact: A configurational analysis of weight management programming within the veterans health administration

#### Laura Damschroder^1^, Edward J. Miech^2^, Richard R. Evans^1^, Michelle B. Freitag^1^, Jennifer A. Burns^1^, Susan D. Raffa^3^, Michael G. Goldstein^3^, Ann Annis^4^, Stephanie A. Spohr^3^, Wyndy L. Wiitala^1^

##### ^1^VA Center for Clinical Management Research, VA Ann Arbor Healthcare System, Veterans Health Administration, Ann Arbor, MI, USA; ^2^VA EXTEND QUERI, Veterans Health Administration, Indianapolis, IN, USA; ^3^VA National Center for Health Promotion and Disease Prevention (NCP), Veterans Health Administration, Ann Arbor, MI, USA; ^4^Michigan State University East Lansing, MI, USA

###### **Correspondence:** Edward J. Miech (edward.miech@va.gov)

**Background:**

Obesity is a well-established risk factor for increased morbidity, particularly diabetes and hypertension, and increased mortality. Although behavioral weight loss programs, pharmacotherapy, and bariatric surgical procedures are effective treatments for obesity, effectively implementing integrated weight management care poses a major challenge to healthcare systems, including the Veterans Health Administration (VHA). The aim of this study was to explore the relationship between weight management program options, facility characteristics, and outcomes across VHA facilities using a novel configurational analysis methodology.

**Methods:**

A systemwide survey of all VHA medical centers was conducted in 2017 to elicit program structural characteristics and options for weight management. Survey responses were linked with facility-level population impact, which was computed as a product of reach (patients who participated in treatment as a percentage of overweight/obese patients) and weight loss (prevalence of patients who lost at least 5% of their baseline body weight at 12 months). Facilities in the top two impact quintiles were compared to those in the bottom two quintiles. Coincidence Analysis methods were used to identify program conditions led to highest impact.

**Findings:**

Of 140 facilities with complete survey data, 69 were included in the analyses with n=33 in the higher impact category and n=36 in the lower impact category. Nine conditions across four categories of factors (facility complexity/size, CLI, pharmacotherapy, and bariatric surgery options) were represented by five different configurations with overall 91% consistency (29 of 32 facilities identified by the model were higher impact) and 88% coverage (29 of the 33 higher impact facilities were explained by the model). Conditions leading to higher impact included configurations of CLI maintenance programming, pharmacotherapy and/or bariatric surgery offered within CLI programs, prescription restrictions, and bariatric surgery referrals. Notably, every configuration was dependent on facility complexity/size.

**Implications for D&I Research:**

No single condition explained implementation of program components across the 33 facilities with higher population impact. Configurational pathways revealed the importance of context and that specific combinations of specific program conditions consistently and uniquely distinguished higher impact facilities from lower impact facilities. These analyses demonstrate how context interplays with local programming decisions, leading to optimal outcomes.

**Primary Funding Source:** Department of Veterans Affairs

### S40 Increasing universal autism screening in primary care: A pragmatic trial with an 18-month follow-up

#### Lisa Ibanez^1^, Wendy Stone^2^

##### ^1^University of Washington, Seattle, WA, USA; ^2^Seattle, WA, USA

###### **Correspondence:** Lisa Ibanez (libanez1@uw.edu)

**Background:** The American Academy of Pediatrics recommends universal autism-specific screening starting at 18 months, yet compliance by primary care providers (PCPs) is limited due to time constraints, lack of comfort identifying early signs, and hesitancy to discuss concerns with parents. These issues have interfered with the adoption of existing validated screeners including the Modified Checklist for Autism in Toddlers–Revised with Follow-up (M-CHAT-R/F; Robins et al., 2014). This two-stage screener comprises a 20-item parent checklist and a time-intensive follow-up interview for positive initial screens (critical for reducing false positives). This pragmatic trial examines the “real world” effectiveness of a system-level intervention that provides a *digital version* of the M-CHAT-R/F (i.e., webM-CHAT-R/F) to automate and shorten the screening process--along with a training workshop on early autism signs, resources, and communication strategies--for increasing routine autism screening at 18 months.

**Methods:** Fifty-nine PCPs from 10 practices across four Washington State counties participated. A stepped-wedge, RCT design was used to randomly assign counties to the timing of the intervention, which comprised a two-hour workshop focused on early detection of autism and use of the webM-CHAT-R/F. PCPs’ perceived self-efficacy regarding autism detection and screening practices were measured by self-report surveys at baseline (T1, T2) and 6-, 12-, 18-months post-training (T3-T5); webM-CHAT-R/F use was measured via REDCap records.

**Findings:** The percent of PCPs using the M-CHAT correctly (i.e., with the follow-up interview) increased from 37% at T1 to 89% at T5, p<.01. A multi-level model indicated that PCPs had higher levels of self-efficacy regarding autism detection relative to baseline at T3-T5, ps< .02. While 7/10 practices were using the webM-CHAT-R/F at T3, 6 practices continued their use through T5, reaching over 7,000 patients. Reasons for discontinuing use were workflow issues (e.g., not integrated with electronic medical records systems), wifi issues, and access to behavioral health staff who conduct the M-CHAT-R/F follow-up interview in person.

**Implications for D&I Research:** This brief system-level intervention may provide a scalable template for increasing adoption of evidence-based practices and tools. It will be critical to identify practice factors associated with early and sustained adoption and identify additional supports/features that may lead to more widespread uptake.

**Primary Funding Source:** National Institutes of Health

### S41 Strategies for successful implementation of advance care planning conversations in primary care clinics

#### Seiko Izumi^1^, Chrystal Barnes^1^, Jeanette Daly^2^, Megan Schmidt^2^, Shelbey Hagen^3^, Kylie Lanman^4^, Taryn Bogdewiecz^5^, Jessica Ma^6^, Rowena Dolor^7^

##### ^1^Oregon Health & Science University, Portland, OR, USA; ^2^University of Iowa, Iowa City, IA, USA; ^3^University of Wisconsin, Madison, WI, USA; ^4^Oregon Rural Practice and Research Network, La Grand, OR, USA; ^5^University of Colorado, Denver, CO, USA; ^6^Duke University, Durham, NC, USA; ^7^Duke University, Duke Clinical & Translational Science Institute, Durham, NC, USA

###### **Correspondence:** Seiko Izumi (izumis@ohsu.edu)

**Background:** Initiating advance care planning (ACP) conversations in primary care is recommended as best practice to support patient-centered care. Yet, many clinics struggle to implement ACP into routine practice. As part of PCORI funded trial (PLC-1609-36277) comparing two approaches to facilitate ACP, we implemented the Serious Illness Care Program (SICP: ariadnelabs.org), an evidence-based ACP intervention, in 30 primary care clinics across the US. We tracked implementation process and outcomes in each clinic to identify strategies for successful implementation of ACP in primary care.

**Methods:** This cluster randomized trial assigned 15 clinics to one of two SICP arms. The research team developed standardized SICP training, plus provided materials/resources to support implementation of the intervention (e.g., implementation practice facilitators). Practice facilitators worked with each clinic to adapt SICP to fit to their needs, resources, and workflow through regular visits facilitating, monitoring, and documenting the process of adaptation and implementation. Visit documents were coded using a modified list of the Expert Recommendations for Implementing Change (ERIC)^1, 2^ and qualitatively analyzed to identify strategies used to implement SICP in each clinic. This study is approved by the Trial Innovation Network Single IRB at Vanderbilt University Medical Center (IRB#181084).

**Findings:** The number and types of strategies used by each clinic varied greatly (median number of strategies used=11.5: IQR=10-17). Implementation strategies used in all clinics included: 1) Distributing standardized educational materials, 2) assessing the readiness and identifying barriers/facilitators, and 3) promoting adaptability. Most clinics (66%) used strategy 4) assessing and redesigning workflow. Clinics that successfully implemented SICP had conducted in-depth and continuous assessments of barriers/facilitators and engaged multiple team members to redesign their clinic workflow.

**Implications for D&I Research:** Clear descriptions of strategies for successful implementation are an important goal of implementation research. This presentation will provide specific descriptions of strategies for successful implementation of ACP in primary care clinics with hopes to increase uptake of ACP implementation in primary care settings.

**Primary Funding Source:** Patient-Centered Outcomes Research Institute

### S42 Primary care team perspectives on integrating opioid use disorder treatment into care delivery via collaborative care

#### Elizabeth Austin^1^, Elsa Briggs^1^, Lori Ferro^2^, Paul Barry^1^, Geoffrey Curran^3^, Andrew Saxon^4^, John Fortney^1^, Dr. Anna Ratzliff^1^, Emily Williams^5^

##### ^1^University of Washington, Seattle, WA, USA; ^2^University of Washington, Seattle, USA; ^3^University of Arkansas for Medical Sciences, Little Rock, AR, USA; ^4^VA Center of Excellence in Substance Addiction Treatment and Education, Seattle, WA, USA; ^5^VA Puget Sound Health Care System, Health Services Research & Development, Center of Innovation for Veteran-Centered & Value-Driven Care, Veterans Health Administration, Seattle, WA, USA

###### **Correspondence:** Elizabeth Austin (austie@uw.edu)

**Background:** With rising incidence of opioid use disorder (OUD) and availability of effective medication treatment (MOUD), there is an urgency to identify best practices for implementing MOUD in clinical care. The collaborative care model (CoCM) is an evidence-based approach to behavioral health care delivery within primary care settings and could extend to address co-occurring disorders (CD), such as OUD. However, the integration of OUD care with CoCM (CoCM-CD) will require engagement and buy-in among primary care teams, and their perspectives are not well explored.

**Methods:** We utilized formative mixed methods evaluation to understand clinic experiences among 10 clinics preparing to implement CoCM-CD. We observed and took careful fieldnotes on implementation calls (held remotely due to the COVID-19 pandemic) over 8 months. Fieldnotes were analyzed weekly using a Rapid Assessment Process, where data were coded using structured templates guided by the Consolidated Framework for Implementation Research (CFIR) and iteratively reviewed with multiple team members. We surveyed primary care team members (n=51) involved in the delivery of CoCM-CD, including primary care providers, behavioral health care managers, and psychiatrists. Survey and qualitative data were triangulated to assess primary care team perspectives on integrating CoCM-CD.

**Findings:** Qualitative data illuminated that providers recognized the need for OUD services in their patient populations, but expressed stigma and hesitancy to treat OUD because they felt it was beyond the scope of their role. Similarly, survey data (85% response rate) found that 96% of providers agreed that CoCM-CD was important and 98.1% believed that providing MOUD saved lives. However, many providers also believed that treating OUD was time consuming (68.6%), that it detracted from other clinical responsibilities (11.8%), was more dangerous than providing care for other chronic conditions (11.8%), and felt discomfort working with patients with OUD (27.4%). Qualitative work that spanned early implementation found that establishing clinical champions, connecting CoCM-CD to the organizational missions of each clinic, and providing access to OUD knowledge experts, all worked to facilitate greater CoCM-CD acceptance.

**Implications for D&I Research:** In order to leverage the opportunity to expand access to OUD care via primary care delivery, greater attention is needed to address stigma, role clarity, comfort, and clinic priorities.

**Primary Funding Source:** National Institutes of Health

### S43 Systemwide external validation of an acute ambulatory antibiotic stewardship implementation toolkit

#### Gregory Tchakalian^1^, Apurva Barve^2^, Ross Fleischman^1^, Larissa May^3^, Daniella Meeker^4^, Kim Newton^5^, Breena Taira^6^, Kabir Yadav^1^

##### ^1^Harbor-UCLA Medical Center, Torrance, CA, USA; ^2^Hack for LA, Fremont, CA, USA; ^3^UC Davis School of Medicine, Sacramento, CA, USA; ^4^University of Southern California, Los Angeles, CA, USA; ^5^LAC+USC Medical Center, Los Angeles, CA, USA; ^6^Olive View-UCLA Medical Center, Los Angeles, CA, USA

###### **Correspondence:** Kabir Yadav (kabir@emedharbor.edu)

**Background:** Inappropriate antibiotic use within emergency department (ED) and urgent care center (UCC) settings remains a major public health concern. Our group previously published a proof-of-concept implementation toolkit to adapt the CDC outpatient stewardship campaign (Get Smart) for academic EDs and UCCs using behavioral nudges. Here we conduct a rigorous implementation adaptation validation for the toolkit in diverse acute care settings within the second largest public health system in the country.

**Methods:** The previously published acute ambulatory care antibiotic stewardship implementation toolkit (http://tinyurl.com/mitigatetoolkit) was applied to the 9 highest inappropriate prescribing sites throughout Los Angeles County. We first conducted a mixed-methods analysis of the barriers and facilitators to adapt stewardship programs for diverse settings and provider types (academic, non-teaching, public employee, contractor, physicians, advanced practice providers). Then, effectiveness of the adapted program, along with implementation outcomes, was measured through a 12-month cluster randomized stepped wedge implementation of stewardship interventions.

**Findings:** Adoption of the intervention was 100% at the site level, with fidelity to the toolkit components being 100% identification of local champions, 97% completion of stakeholder interviews, 58% response rate of confidential surveys from frontline providers, and 100% sending of monthly individualized peer comparison emails. Grounded theory content analysis of interviews was triangulated with survey results to guide all-setting and setting-specific adaptations of the stewardship intervention. Across 584 providers and 67,767 patient encounters, there was a decrease in prescribing from 8.1% to 4.3%, with an adjusted decrease of 2.1% (95% CI 1.6-2.5) in inappropriate antibiotic prescribing. Penetrance of the intervention, as measured by consent of providers at each site was median 53% (IQR 26-74). According to the survey, acceptability of the intervention was 92% and appropriateness was 93%.

**Implications for D&I Research:** We validated an effective, generalizable framework for adaptation of existing antibiotic stewardship strategies to match the clinical workflow of acute ambulatory care settings that accounts for the unique challenges inherent within those environments. We also explore potential setting- and provider-level factors that could better inform where and to whom to apply targeted behavioral interventions.

**Primary Funding Source:** National Institutes of Health

### S44 How does the eric typology apply to implementation of clinical decision support for genomic medicine?: Specifying and reporting implementation strategies for desired outcomes among a genomic medicine implementation network

#### Nina Sperber^1,2,3,4^, Olivia Dong^5^, Megan Roberts^6^, Paul Dexter^7,8^, Amanda Elsey^9^, Geoffrey Ginsburg^3^, Carol Horowitz^10^, Julie Johnson^9^, Ken Levy^11^, Henry Ong^12^, Josh Peterson^13^, Toni Pollin^14^, Tejinder K. Rakhra-Burris^3^, Michelle Ramos^10^, Todd Skaar^11^, Lori Orlando^3^

##### ^1^Duke University School of Medicine, Department of Population Health Sciences, Durham, USA; ^2^Center of Innovation to Accelerate Discovery and Practice Transformation (ADAPT), Veterans Health Administration, Durham, NC, USA; ^3^Duke University School of Medicine, Durham, NC, USA; ^4^Duke-Margolis Center for Health Policy, Durham, NC, USA; ^5^RTI Health Solutions, Durham, NC, USA; ^6^University of North Carolina at Chapel Hill Eshelman School of Pharmacy, Chapel Hill, NC, USA; ^7^Regenstrief Institute, Inc., Indianapolis, IN, USA; ^8^Clem McDonald Center for Biomedical Informatics, Indianapolis, USA; ^9^University of Florida, Gainesville, FL, USA; ^10^Icahn School of Medicine at Mount Sinai, New York, NY, USA; ^11^Indiana University School of Medicine, Indianapolis, USA; ^12^Vanderbilt University Medical Center,, Nashville, USA; ^13^Vanderbilt University Medical Center, Nashville, USA; ^14^University of Maryland School of Medicine, Baltimore, USA

###### **Correspondence:** Nina Sperber (nina.sperber@duke.edu)

**Background:** Emergence of genomic medicine as a new approach to healthcare follows technological advances in sequencing the human genome and harnessing big datasets. Clinical decision support (CDS) commonly guides clinicians in use and interpretation of personalized data; however, best strategies for integrating into routine care need an evidence-base. We sought to identify and describe core implementation strategies for desired outcomes among members of the Implementing Genomics in Medicine (IGNITE) network.

**Methods:** Participants included six diverse projects led by academic medical centers allied with community healthcare systems. All projects implemented CDS tools into an EHR system: three implemented different pharmacogenomics (PGx) CDS interventions in the EHR and three focused on disease risk or etiology. To obtain detail about implementation strategies and desired outcomes, we adapted a published survey derived from a typology of 73 implementation strategies grouped into thematic clusters, the Expert Recommendations for Implementing Change (ERIC), and conducted follow-up interviews guided by implementation strategy reporting criteria (Proctor 2013) and a planning framework, RE-AIM.

**Findings:** On average, the projects implemented 32 ERIC strategies (range 11–47). The three PGx projects each used more strategies (40-47) compared to the disease-focused ones (11–29). Despite diverse project goals and approaches, all six projects commonly used four strategies from three clusters: (1) developing strategies to obtain and use stakeholder feedback (cluster—using evaluative and iterative strategies), (2) identifying early adopters (cluster—developing stakeholder interrelationships), (3) conducting educational meetings (cluster—training and educating stakeholders), and (4) having an expert meet with clinicians to educate them (cluster—training and educating stakeholders). Detailed reporting criteria revealed different manifestations of the strategies across the projects and a need to integrate the training and educating stakeholder strategies in reporting.

**Implications for D&I Research:** This project represents the first application of the full ERIC typology in conjunction with Proctor’s detailed reporting criteria to genomic medicine implementation. ERIC, developed in the context of mental health research and practice, provides a useful guide for highlighting generalizable core strategies as a starting point; however, it did not capture all relevant strategies. We present ideas for future work to develop a version of the ERIC typology specifically for genomic medicine implementation.

**Primary Funding Source:** National Institutes of Health

### S45 Residual influences of quality improvement collaboratives on practice change: A longitudinal study of Maryland hospitals participating in a collaborative to reduce primary cesarean delivery

#### Jennifer Callaghan-Koru^1^, Inaya Wahid^2^, Andreea Creanga^3^, Bonnie DiPietro^4^, Geoffrey Curran^5^

##### ^1^Sociology, Anthropology, and Health Administration and Policy, University of Maryland-Baltimore County, Baltimore, MD, USA; ^2^University of Maryland, Baltimore County, Baltimore, MD, USA; ^3^Johns Hopkins Bloomberg School of Public Health, Baltimore, MD, USA; ^4^Maryland Patient Safety Center, Elkridge, MD, USA; ^5^University of Arkansas for Medical Sciences, Little Rock, AR, USA

###### **Correspondence:** Jennifer Callaghan-Koru (jck@umbc.edu)

**Background:** Quality improvement collaboratives (QICs) are a common implementation strategy for evidence-based practices, and state-based QICs are a key component of the national strategy to improve perinatal health. The mechanisms of QICs are thought to include shared learning across participating hospitals and positive peer pressure that result from collaborative activities like performance reporting and expert seminars. The extent to which participating organizations continue to make new practice changes after collaborative activities have ended is not well studied.

**Methods:** Between June 2016 and December 2018, 31 birthing hospitals in Maryland voluntarily participated in a statewide QIC to reduce primary cesarean deliveries. As a condition of participation, hospitals agreed to implement new practices from a consensus patient safety bundle with 23 recommended unit-level practices. To assess hospitals’ adoption and maintenance of practices in the bundle, we distributed surveys to the hospital-designated collaborative leads at the end of the collaborative (November 2018) and sixteen months later (March 2020).

**Findings:** Full responses to both surveys were obtained from 27 hospitals (87% response rate). Respondents for 24 of these hospitals (89%) indicated that their labor & delivery unit continued working on bundle implementation after the formal end of the collaborative. The median number of practices implemented was 12 (range: 0 to 22) at the end of the collaborative, and 17 (range: 9 to 22) at the follow up survey. At follow up, hospitals also reported discontinuing a median of 1 practice that was in place at the end of the collaborative (range: 0 to 12). Practices with the highest post-collaborative adoption were protocols to encourage early labor at home (15 hospitals) and ongoing staff training on labor support techniques (14 hospitals). The practice with the highest discontinuation (9 hospitals) was training on external cephalic version technique.

**Implications for D&I Research:** These data suggest that QICs may have residual impacts on practice changes after the completion of planned activities. Follow up assessments of QICs should measure adoption of new practices in addition to maintenance of practice changes. More research is needed to understand whether this effect is widespread and the implications for the design of QICs (e.g., activities, length) to maximize their impact.

**Primary Funding Source:** National Institutes of Health

### S46 Refining implementation of complex clinical practices addressing VA clinical priorities: A dynamic diffusion network to address moral injury and suicide

#### George Jackson^1,2^, Melissa Smigelsky^1,3^, Keith Meador^3,4^, Summer Anderson^1^, Victoria Trimm^1,3^, Ryan Vega^5^, Blake Henderson^5^, Jason Nieuwsma^1,2,3^

##### ^1^Durham Veterans Affairs Health Care System, Veterans Health Administration, Durham, NC, USA; ^2^Duke University, Durham, NC, USA; ^3^VA Integrative Mental Health, Durham, NC, USA; ^4^Vanderbilt University, Nashville, TN, USA; ^5^Veterans Health Administration Innovation Ecosystem, Washington, DC, USA

###### **Correspondence:** George Jackson (george.l.jackson@duke.edu)

**Background:** The Dynamic Diffusion Network (DDN) implementation strategy brings together healthcare facilities seeking to address a shared, complex, clinical challenge for which there are core evidence-based principles and/or practices (EBPs) available. However, the challenge is a lack of clarity concerning the specific ways in which application of EBPs can or should vary across facilities to ensure effective implementation.

**Methods:** The first DDN occurred from June 2019-November 2020 with the goal of refining suicide prevention strategies and moral injury care practices being conducted by 12 chaplain-mental health provider teams across the Veterans Health Administration (VHA). It included a cyclical improvement model based on: identifying quality goals; describing practices; measuring impact; quality improvement; and telling the improvement story. This was combined with structured facilitation calls, subject matter expertise, and shared accountability. The DDN was evaluated based on analysis of improvement and clinical activities (733 weekly reports and 46 quarterly phase summary reports (~4 per team). All participants completed a program satisfaction survey (n=22) and 20 participated in a semi-structured qualitative interview.

**Findings:** Participants reported: 1) improvements in facility practices (more clearly defined clinical practices and materials, refined quality/practice objectives, identification of core and adaptable components, and sustainability efforts); 2) feeling “part of something” (opportunity and accountability to make changes, access to constructive, outside perspectives, broader applicability of improvement process, being part of a “greater good”); and 3) coping with COVID-19 through an established network with structural support. All participants agreed or strongly agreed (SA) that they were confident practices improved (SA=82%), were proud of DDN work (SA=77%), and would recommend DDN participation to a colleague (SA=85%). Cross-pollination of ideas was most beneficial when practices shared commonalities in objectives and procedures (i.e., groups addressing moral injury). Despite COVID-19, 87 “products” (e.g., papers, reports, presentations) have resulted from the DDN, an indication of the reach of the effort.

**Implications for D&I Research:** Even during COVID-19, the DDN was an effective strategy for supporting implementation and refinement of complex clinical interventions aimed at addressing VA clinical priorities. It provides a mechanism to address the uncertainty and need for continuous learning as complex innovations spread across health system sites.

**Primary Funding Source:** Department of Veterans Affairs

### S47 Change in implementation leadership, climate, and provider reach for motivational interviewing: A cluster randomized trial of the leadership and organizational change for implementation (LOCI) strategy in substance use disorder treatment

#### Marisa Sklar^1,2,3^, Mark Ehrhart^4^, Scott Roesch^5^, Gregory Aarons^6,7,8^

##### ^1^University of California, San Diego, La Jolla, CA, USA; ^2^Child and Adolescent Services Research Center, San Diego, CA, USA; ^3^UC San Diego ACTRI Dissemination and Implementation Science Center, La Jolla, CA, USA; ^4^University of Central Florida, Orlando, FL, USA; ^5^San Diego State University, San Diego, CA, USA; ^6^UC San Diego, San Diego, CA, USA; ^7^UC San Diego Altman Clinical and Translational Research Institute Dissemination and Implementation Science Center, La Jolla, CA, USA; ^8^UC San Diego, La Jolla, CA, USA

###### **Correspondence:** Marisa Sklar (masklar@health.ucsd.edu)

**Background:** Successful implementation and sustainment of evidence-based practice (EBP) requires alignment of effective leadership at multiple organization levels. Leadership and Organizational Change for Implementation (LOCI) is a multifaceted implementation strategy to support the implementation and sustainment of EBPs. By engaging and developing leadership at clinic and organization levels, LOCI aims to develop a climate for EBP implementation and sustainment within organizations.

**Methods:** The effect of LOCI on the implementation of motivational interviewing (MI) for substance use disorder treatment was tested in a cluster randomized trial. Sixty clinics from nine agencies were randomly assigned to either LOCI, or a leadership webinar condition (control). Repeated survey measures from clinic providers (*n*=380; *n*_*Control*_=179, *n*_*LOCI*_=201) assessed implementation leadership of clinic leaders, and implementation climate of clinics, during engagement in the assigned condition across four timepoints. Three-level multilevel modeling wherein repeated measures (Level-1) were nested within providers (Level-2), nested within clinics (Level-3), was used to assess polynomial trends in leadership and climate over time, and whether these trends differed as a function of condition. Chi-square was used to assess between condition differences in MI reach as defined as the number of providers who engaged in fidelity monitoring.

**Findings:** Between condition differences in quadratic trends were found for supportive (*βhat*=-.22, *p<*.05) and proactive (*βhat*=-.22, *p<*.05) leadership, and the educational support dimension (*βhat*=-.15, *p<*.05) of implementation climate. Follow-up simple slope analyses revealed significant negative quadratic trends for LOCI, but non-significant change over time for the control condition. Reach of MI was significantly greater in LOCI (*n*=134) than control (*n*=101) (*Χ*^*2*^=4.21, *p*=.040).

**Implications for D&I Research:** LOCI was effective in enhancing implementation leadership and climate within organizations, and in enhancing MI reach. Limitations and future directions will be discussed.

**Primary Funding Source:** National Institutes of Health

### S48 Transforming VA to a whole health system of care: The use of implementation strategies to drive national change

#### Rendelle Bolton^1,2^, Juliet Wu^1^, Aishwarya Khanna^1^, A. Rani Elwy^1,3^, Barbara Bokhour^1^, Justeen Hyde^1^

##### ^1^VA Bedford Healthcare System, Veterans Health Administration, Bedford, MA, USA; ^2^Brandeis University Heller School for Social Policy and Management, Waltham, MA, USA; ^3^Warren Alpert Medical School of Brown University, Providence, RI, USA

###### **Correspondence:** Rendelle Bolton (Rendelle.Bolton@va.gov)

**Background:** Since 2011, the Veterans Health Administration (VA) has been transforming to a Whole Health System of Care (WHS) to optimize the health and wellbeing of Veterans and staff alike. This transformation is defined by patient-centered clinical encounters and implementation of discrete services (e.g., complementary integrative health therapies, health coaching), requiring new infrastructure, trainings, and policy. We sought to understand how VA’s national Office of Patient-Centered Care and Cultural Transformation (OPCC&CT) supported transformation across VA’s 170 medical centers (VAMCs) and >1200 community-based clinics.

**Methods:** During a multi-year ethnographic evaluation of VA’s WHS implementation, we conducted semi-structured interviews with 20 OPCC&CT leaders/staff to identify implementation activities at the VAMC, regional, and national levels. We coded activities into a priori categories aligned with 73 implementation strategies recognized by the Expert Recommendations for Implementing Change group. Inductive coding captured barriers/facilitators to strategy use, organizational context, and transformation approach.

**Findings:** OPCC&CT used 64 of 73 implementation strategies with key stakeholders across all levels of VA (central/regional offices, VAMCs, consumers, policy makers, and community partners). Strategies were often bundled or nested together. To facilitate WHS implementation in VAMCs, OPCC&CT conducted trainings/education, readiness assessments, and repeated evaluations; provided resources, implementation guidance, and interactive/technical assistance; and prepared champions, leaders, early adopters, and local workgroups. Regionally and nationally, strategies created a context which enabled system transformation by changing policies, developing infrastructure (e.g., VA-wide records/billing mechanisms, new position descriptions), and establishing relationships and buy-in among key stakeholders. Organizationally, OPCC&CT developed matrixed workgroups to coordinate strategy use among its 70-person staff. National champions and subject-matter experts spanned boundaries between OPCC&CT and the field, providing input on implementation priorities and disseminating information outward. OPCC&CT iteratively developed, piloted, evaluated, refined, and tailored the WHS and the implementation strategies used. Barriers included promoting WHS uptake among front-line staff due to regional priorities that limited OPCC&CT’s ability to directly support clinicians and implement incentives locally.

**Implications for D&I Research:** Findings extend the use of implementation strategies beyond local evidence-based practice implementation to the system level. When paired with supportive organizational structures and continuous learning processes, these strategies can facilitate system transformation by creating policies, infrastructure, and engaging stakeholders to enable implementation.

**Primary Funding Source:** Department of Veterans Affairs

### S49 Project MIMIC: Preliminary data from the first two cohorts of a hybrid type 3 effectiveness-implementation trial

#### Sara Becker^1^, Bryan Garner^2^

##### ^1^Brown School of Public Health, Providence, RI, USA; ^2^RTI International, Research Triangle Park, NC, USA

###### **Correspondence:** Sara Becker (sara_becker@brown.edu)

**Background:** Contingency management (CM) is the most effective behavioral adjunctive treatment in combination with medication for opioid use disorders, but is one of the least available treatments in opioid treatment programs. Project MIMIC (Maximizing Implementation of Motivational Incentives in Clinics) is a 30-site, multi-cohort, hybrid effectiveness-implementation cluster randomized trial testing two multi-level strategies to help opioid treatment programs (OTPs) implement CM. This submission examines process data from Project MIMIC’s first two cohorts of OTPs.

**Methods:** One hundred thirty staff from 18 OTPs were cluster randomized to receive either the Addiction Technology Transfer Center (ATTC) strategy (workshop + feedback + coaching) or the Enhanced ATTC (E-ATTC) strategy, which layered in two additional theory-driven strategies: Pay-For-Performance and Implementation Sustainment Facilitation. Consistent with the exploration, preparation, implementation, and sustainment (EPIS) framework, OTPs engaged in 5 months of preparation and 7 months of implementation activities.

**Findings:** All 18 OTPs completed preparation activities and advanced to the implementation phase. During the preparation phase, a significantly greater proportion of E-ATTC staff completed the didactic CM workshop than ATTC staff (98% vs. 85%, χ^2^(1)=7.0, p=.008). Additionally, skill ratings of CM role plays submitted at the end of the Preparation phase were significantly higher among E-ATTC than ATTC staff, (*t*(62)=2.59, p=.01). In the implementation phase, each OTP sought to enroll 25 patients: OTPs in the E-ATTC condition enrolled a greater proportion of the target than those in the ATTC condition (87% vs. 77%, χ^2^(1)=7.6, p=.006). In addition, a greater proportion of E-ATTC staff met the CM exposure benchmark (28% vs. 12%, χ^2^(1)=5.2, p=.02) Measurement of sustainment is ongoing.

**Implications for D&I Research:** Preliminary process data from 18 OTPs indicate that the theory-driven E-ATTC strategy has been associated with higher training engagement, CM skill on a role play, patient enrollment, and CM exposure. These results suggest that the ATTC implementation strategy, a real-world strategy widely used by a network of SAMHSA-funded training and technical assistance centers, can potentially be enhanced by the inclusion of Pay-for-Performance and Implementation Sustainment Facilitation. Future work is needed to provide a more comprehensive assessment of the E-ATTC strategy's effect on patient-level outcomes, long-term sustainment, and cost effectiveness.

**Primary Funding Source:** National Institutes of Health

### S50 Learning health system programs in delivery systems - contributions and limitations of externally-funded implementation research

#### Michael I. Harrison, Amanda Borsky

##### Agency for Healthcare Research and Quality, Rockville, MD, USA

###### **Correspondence:** Michael Harrison (Michael.Harrison@ahrq.hhs.gov)

**Background:** Learning Health System (LHS) programs deploy researchers in fields like health services, implementation science, human factors, and engineering to address improvement priorities of delivery system leaders (clinicians and administrators). We examine contributions to LHS work and limitations of externally-funded research on implementation of care-delivery change.

**Methods:** Conducted 44 hour-long, semi-structured interviews with 41 system leaders, LHS directors, LHS investigators – reached through snowballing. Rapid qualitative analysis: interviews summarized in structured templates; templates consolidated into study-site matrices. Additional sources: interviews with 12 LHS experts and practitioners, published/grey literature. Examined LHS program profiles: 34 successful LHS projects (including 12 with external funding); 15 projects described as failures or examples of major challenges.

**Findings:**

Multi-year cycles and strict methodological standards limited value of externally-funded LHS projects for system leaders, who often sought rapid responses to problems and were not focused on scientific rigor. Gaps in communication and understanding emerged between system leaders and LHS researchers dedicated to traditional research. In successful projects, LHS investigators often met system needs through short-term applications of pragmatic practices (e.g., rapid analysis of available data, quality improvement, implementation facilitation, human factors analysis). These required additional skills and more collaborative work styles than traditional research and yielded less scientific recognition and funding.

When turnaround time was not critical, some LHS projects constructively used external funding on implementing care-delivery change. The funding supported evaluating current practices; identifying, developing, and implementing care or operational redesigns; deploying practice guides/tools. Additionally, externally-supported projects used internal funding to implement and sustain changes. Sometimes, after internally-funded, quality improvement projects, LHS researchers obtained external funding for multi-site improvement testing and spread.

Regardless of funding source, successful LHS initiatives responded to system priorities by acting quickly and pragmatically. Impactful LHS programs developed strong formal and informal ties to system leaders; proactively identified leaders’ needs and priorities; and translated these into doable project proposals.

**Implications for D&I Research:** Despite challenges, external funding for D& I research can contribute to LHS work within delivery systems. Additionally, highly responsive and reliable LHS work requires consistent internal funding of services and activities that are not often supported by D&I research awards.

### S51 Organizational-level factors associated with provider and staff burnout in HIV clinics at the epicenter of the United States HIV epidemic

#### Jessica Sales^1^, Elizabeth Adam^2^, Chris Root^2^, Katherine Anderson^2^, Ameeta Kalokhe^1^

##### ^1^Emory University, Rollins School of Public Health, Atlanta, GA, USA; ^2^Atlanta, GA, USA

###### **Correspondence:** Jessica Sales (jmcderm@emory.edu)

**Background:** The multidimensional needs of people with HIV, some stemming from histories of trauma, have been linked to work overload, stress, and burnout among the healthcare professionals who serve them, thus underscoring the importance of adoption of trauma-informed care (TIC) in HIV clinics. Having organizational practices that help staff manage stress and emotional fatigue that contribute to burnout is a central tenant of TIC. Such practices include trauma training (i.e., how to set healthy professional boundaries) and staff support (e.g., debrief after a difficult patient, resources to manage stress). This study aims to examine the association between adoption of these TIC practices within HIV clinics and burnout among healthcare professionals, and to explore organizational factors associated with adoption of TIC practices supportive of their well-being.

**Methods:** As part of a larger mixed-methods study, from December 2019-April 2020, we conducted surveys with 318 healthcare professionals of 46 HIV clinics across 8 southeastern states to examine associations between self-reported individual and patient characteristics (i.e., demographics, role, tenure in clinic, perceived trauma among patient population), clinic adoption of TIC practices (training on boundary setting, staff support practices), and burnout (using the ProQOL). We also examined the relationship between organizational factors from the Consolidated Framework for Implementation Research (i.e., leadership engagement, implementation climate, and available resources) and adoption of TIC practices.

**Findings:** In bivariate analyses, receipt of training on establishing professional boundaries and adoption of more TIC staff support practices were significantly associated with lower burnout scores. In a multivariable regression accounting for significant individual/patient characteristics (race, perceived level of trauma among clinic patients), receipt of training on setting healthy professional boundaries remained significantly associated with lower burnout scores. Greater leadership engagement, more positive implementation climate, and having more available resources (e.g., staff/training/time) were all significantly associated with greater adoption of TIC training and staff support practices.

**Implications for D&I Research:** HIV healthcare professionals are critical for the delivery of multidimensional evidence-based care to improve patient outcomes; thus, identifying organizational factors associated with adoption of TIC practices that support healthcare professionals’ well-being is urgently needed, yet under studied. Our research begins to fill this gap.

**Primary Funding Source:** National Institutes of Health

### S52 Dynamic experiences of implementation: The role of time in caregivers’ engagement with screening for autism spectrum disorder

#### Leah Ramella^1^, Ana Schaefer^1^, Marisa Petrucelli^2^, Abbey Eisenhower^2^, Alice Carter^3^, R. Chris Sheldrick^4^, Thomas Mackie^1^

##### ^1^SUNY Downstate Health Sciences University School of Public Health, Brooklyn, NY, USA; ^2^University of Massachusetts Boston, Boston, MA, USA; ^3^University of Massachusetter Boston, Boston, MA, USA; ^4^Boston University School of Public Health, Boston, MA, USA

###### **Correspondence:** Leah Ramella (leah.ramella@downstate.edu)

**Background:** System-level interventions to improve child outcomes require sustained engagement of caregivers over time. Yet, as interventions unfold, the factors influential to treatment, engagement and maintenance may vary. Time is central to the conceptualization of many implementation frameworks, but seldom the specific focus of research studies. Empirically, longitudinal qualitative methods offer a tool to assess temporality and to investigate the dynamic nature of interventions. This paper proposes a seven-step framework for qualitative methods to examine dimensions of time during a complex pediatric intervention.

**Methods:** We engage a seven-step framework tailored to the exploration of time in implementation, ranging from articulation of a time-oriented research question to identification of time-centered analyses. To illustrate application, we offer a case study of the experience of 22 caregivers engaged in a multi-stage autism screening process who participated in a series of longitudinal qualitative interviews (n=63). Our data analyses examined whether factors emerged: (a) across all caregivers at specific intervention stages (pooled cross-sectional analysis), (b) in particular sequences based on the experiences of caregivers over time (trajectory analysis), or (c) a combination of both.

**Findings:** First, results demonstrate that factors routinely emerged across participants at specific intervention stages. For example, administration of the observation-based screening tool in the second stage of the intervention routinely presented an emotional burden for caregivers that impeded progress towards diagnostic resolution. Second, results demonstrated that prior experiences dynamically influenced caregiver engagement over time. Caregivers who had received a borderline score of concern for autism on an early-stage screening tool proceeded to later stages with unique barriers.

**Implications for D&I Research:** Longitudinal qualitative interviews facilitate in-depth understanding of caregiver experiences, providing insight into how and when specific barriers arose. The seven-step analytical framework provides a roadmap for employing longitudinal qualitative methods to investigate the role of time in implementation, with guidance on: (a) optimizing the frequency of data collection, (b) handling attrition, and (c) key decision-points in analyzing longitudinal data. By engaging in time-centered investigations, valuable insights are gained in determining under what conditions to implement specific implementation strategies.

**Primary Funding Source:** National Institute of Mental Health

### S53 Delivery of cancer screening and prevention during the COVID-19 pandemic on – mixed methods analysis

#### Nathalie Huguet, Leah Gordon, Tahlia Hodes, Andrea Baron, Maria Danna, Heather Holderness, Deborah Cohen

##### Oregon Health & Science University, Portland, OR, USA

###### **Correspondence:** Heather Holderness (holdernh@ohsu.edu)

**Background:** Prior to COVID-19, cancer preventive care was primarily delivered in-person to Community Health Center (CHC) patients. The pandemic elicited dramatic shifts in care delivery. Existing data provide little insight into the types of care delivered during the pandemic and what CHCs implemented, adopted, or adapted to deliver preventive care. The objective of this study is to describe the COVID-19 pandemic’s impact on delivery of cancer preventive care and identify processes CHCs used to implement and adapt cancer preventive care.

**Methods:** This mixed methods study uses quantitative electronic health record data from 224 CHCs from the OCHIN Network. Qualitative data collection from a subsample of 8 CHCs with high cancer preventive care performance pre-COVID-19. Practices were purposively selected for variation on geographic region, rurality, and patient demographics. Interviews with 26 practice members from these 8 CHCs were conducted. Outcome measures included: telemedicine and in-person visit rates; cervical and colorectal cancer procedures rates; factors influencing adoption and implementation of telemedicine, and changes to cancer preventive care delivery.

**Findings:** Across the network, telemedicine visit rates increased by 1237% at the onset of the pandemic. By May 2020, rates of cervical and colorectal cancer procedures declined by 61% and 58%, respectively. Interviews showed the importance of Previous Quality Improvement experience, which equipped practices with formal change management tools for introducing alternative care modalities and contributed to staff familiarity and comfort with change. Additionally, CHCs utilized a variety of care-delivery modalities to continue providing cancer preventive screenings not suited to telemedicine (e.g., drive-up and curbside visits, mobile vans, home visits). When hospital referrals for preventive services halted, clinics shifted to offering alternative screening methods that could be managed in-clinic (e.g., mailing fecal kits, arranging on-site mobile mammogram clinics). Lastly, CHCs coordinated outreach efforts to keep patients aware of clinical changes, and managed patient hesitancy about in-person care through a shift in provider messaging.

**Implications for D&I Research:** Innovative CHCs were able to adapt and iterate care delivery during the pandemic and recover from the initial decline in cancer screenings. Approaches to rapid implementation could inform future non-pandemic practice change.

**Primary Funding Source:** National Institutes of Health

### S54 Mixed methods examination of the acceptability of e-connect: A systems intervention to link youth on probation to behavioral health care, guided by the gateway provider model

#### Corianna Sichel^1^, Margaret Ryan^1^, Gail Wasserman^2^, Alexandra Arnold^1^, Kuljit Kaur^1^, Faye Taxman^3^, Michael Dennis^4^, Kate Elkington^2^

##### ^1^Columbia University/New York State Psychiatric Institute, New York, NY, USA; ^2^Columbia University, New York, NY, USA; ^3^George Mason University, Fairfax, VA, USA; ^4^Chestnut Health Systems, Bloomington, IL, USA

###### **Correspondence:** Corianna Sichel (cs4038@cumc.columbia.edu)

**Background:** Youth under community supervision (YCS, i.e., on probation) experience disproportionately high levels of suicide risk and behavioral health (BH) need, and low levels of service uptake, in part due to system-level barriers. Addressing the BH needs of YCS requires cross-system linkage as risk is identified in probation and treatment occurs in community-based care systems. Informed by the Gateway Provider Model (GPM) and the EPIS framework, e-Connect, a digital screen, referral, and linkage system, was developed and piloted in New York State (NYS) as a systems-level intervention to identify YCS’s suicide risk and BH need and facilitate cross-system linkage.

**Methods:** The study was guided by GPM and used a sequential mixed-methods approach for the purpose of complementarity. Surveys from n=58 probation staff, across 10 counties in NYS, who participated in the implementation of e-Connect (36.2% male; 87.9% White/Caucasian; age 24-73, M=43.04, SD=11.08) examined three GPM domains: 1) structural characteristics (e.g., agency communications), 2) psychological climate, and 3) gateway provider perceptions/knowledge, associated with staff ratings of the acceptability of e-Connect. Qualitative data, drawn from six focus groups with n=35 probation and n=11 BH staff, explored acceptability and elaborated on trends in the quantitative data.

**Findings:** Bivariate analyses identified measures of different GPM factors associated with staff ratings of e-Connect acceptability (measured via a 10-item scale assessing staff perceptions of the system). Structural factors were not significantly associated with acceptability. Two linear regressions with robust standard errors further explored the importance of GPM domains in predicting acceptability of e-Connect. In the model addressing psychological climate, perceptions of time burden (B=-0.60, *t*(48)=-7.41, *p*<0.001), and cynicism about the organization (B=-0.26, t(48)=-2.13, *p*=0.04) were significantly associated with acceptability. In the model addressing gateway provider perceptions/knowledge, only perceived usefulness of the e-Connect referral form significantly predicted acceptability (B=0.75, t(48)=3.06, p=0.004). Qualitative feedback from probation and BH staff provided nuanced information about how and why factors contributed to acceptability of the systems-level intervention.

**Implications for D&I Research:** Time burden, usefulness, and cynicism should be prioritized as targets in future iterations of e-Connect and similar systems-level interventions to increase acceptability. Additional implications for dissemination and implementation will be discussed.

**Primary Funding Source:** National Institutes of Health

### S55 Beliefs and attitudes for successful implementation in schools (BASIS): A theoretically-driven, pre-implementation strategy targeting front-line practitioners’ motivation to increase the yield of training and consultation

#### Aaron Lyon

##### University of Washington, Seattle, WA, USA

###### **Correspondence:** Aaron Lyon (lyona@uw.edu)

**Background:** Even in a conducive organizational context, individual behavior change is required for successful implementation. Focusing on individual-level mechanisms of behavior change represents a parsimonious approach to augment standard implementation supports. The education sector is the most common setting for youth behavioral health services, but evidence-based practices (EBPs) are rarely adopted and delivered. Beliefs and Attitudes for Successful Implementation in Schools (BASIS) is a pragmatic, multifaceted, and intervention-agnostic strategy that augments EBP-specific training and consultation and is designed to target mechanisms derived from the Theory of Planned Behavior (TPB) and Health Action Process Approach (HAPA) (attitudes, social norms, self-efficacy, intentions) to enhance implementation and service recipient outcomes. This presentation will discuss findings to date across a series of federally-funded studies of the BASIS strategy with different populations and EBPs, which have refined our understanding of BASIS’s mechanisms.

**Methods:** A series of studies have examined the impact of BASIS on its mechanisms of action and implementation outcomes. These include a pre-post study with 1,181 educators and 62 schools implementing universal behavioral health programs, a pilot randomized trial with 25 school-based clinicians implementing an indicated trauma intervention, and a randomized trial of 83 teachers implementing an evidence-based classroom program. Each randomized trial compared BASIS to an attention control and evaluated effects on attitudes, social norms, self-efficacy, intentions, and intervention adoption. Two additional large-scale randomized trials are ongoing.

**Findings:** BASIS has consistently demonstrated feasibility and acceptability, as well as effects on a subset of target mechanisms and implementation outcomes. For instance, in the pre-post trial, BASIS led to more favorable EBP attitudes (d=1.03), which were associated with two measures of EBP fidelity (d=0.51-0.67). Results have varied, but BASIS has had its strongest and most consistent effects on practitioner self-efficacy and initial adoption of EBPs, although its effects tend to attenuate over time.

**Implications for D&I Research:** Existing compilations of implementation strategies contain very few individually and motivationally focused techniques, and even fewer are explicitly designed to impact well-specified mechanisms of action. BASIS isolates individual-level mechanisms of implementation; the understanding of which can inform the design and tailoring of efficient strategies across settings and EBPs.

**Primary Funding Source:** National Institutes of Health

### S56 Developing capacity for evidence-based practice (EBP) with leadership and organizational change for implementation (LOCI)

#### Marisa Sklar^1,2^, Mark Ehrhart^3^, Gregory Aarons^4^

##### ^1^Child and Adolescent Services Research Center, San Diego, CA, USA; ^2^University of California, San Diego, La Jolla, CA, USA; ^3^University of Central Florida, Orlando, FL, USA; ^4^UC San Diego, San Diego, CA, USA

###### **Correspondence:** Marisa Sklar (masklar@health.ucsd.edu)

**Background:** Implementation of evidence-based practices (EBPs) represents a strategic change in organizations. Leaders across levels within an organization play crucial roles in advancing strategic change initiatives, and effective leadership predicts long-term EBP sustainment. As such, there is a need to combine leadership development with organizational strategies to support EBP implementation. Leadership and Organizational Change for Implementation (LOCI) is a packaged and multifaceted implementation strategy that was developed to support the implementation and sustainment of EBPs. This presentation will review the core principles and components of the LOCI implementation strategy, as well as discrete capacity-building strategies that were employed by participating leaders to develop a climate for EBP implementation and sustainment within their organizations.

**Methods:** The LOCI implementation strategy has been used in a number of service settings and with a variety of EBPs across four NIH-funded trials, and across health trusts in Norway. By engaging leadership at clinic and organization levels, LOCI helps organizations to develop a climate for EBP implementation and sustainment that communicates to clinical providers that EBP use is expected, supported, and rewarded. LOCI utilizes repeated data collection and feedback cycles, leadership training and coaching, and organizational strategy development. LOCI components are designed to improve participants’ transformational and implementation leadership behaviors, subsequently creating an EBP implementation climate within their organizations such that EBPs are delivered with fidelity.

**Findings:** Through engagement in LOCI components, participants representing multiple levels of leadership within service organizations were successful in developing strategies to support EBP implementation. Exemplar capacity-building steps that leaders have taken in LOCI include developing provider exchange programs to enhance lateral communication and diverse learning opportunities, adding EBP-specific language to job descriptions and interview guides, and securing 2 hours/month of productivity credit for providers’ EBP skill development.

**Implications for D&I Research:** Implementation efforts are most successful when leadership, policies, and practices are aligned. Multilevel implementation strategies like LOCI are crucial for establishing alignment for effective implementation and sustainment of EBPs.

**Primary Funding Source:** National Institutes of Health

### S57 “because you can’t rely on just billing:” a fiscal mapping process for sustainable financing of evidence-based practices

#### Marylou Gilbert

##### RAND, Santa Monica, CA, USA

###### **Correspondence:** Marylou Gilbert (marylou@rand.org)

**Background:** There are significant cost-related barriers to sustaining evidence-based practices (EBPs) in behavioral health service agencies. Such agencies need to cultivate strategic planning capacities that support sustained funding for EBPs. This project is developing and evaluating the Fiscal Mapping Process: a multi-step, structured tool that guides behavioral health service agencies through coordinating the optimal combination of financing strategies for EBP sustainment.

**Methods:** We adapted the Fiscal Mapping Process prototype from an established intervention mapping process, and incorporated existing resources into the prototype (e.g., a compilation of 23 financing strategies for behavioral health EBPs). We are engaging 12 behavioral health service agencies in a year-long pilot-test of the Fiscal Mapping Process with either of two youth-focused EBPs: Parent-Child Interaction Therapy (PCIT) or Trauma-Focused Cognitive-Behavioral Therapy (TF-CBT). We provide initial training and monthly coaching for the tool. Throughout the year, we engage service agency representatives and their stakeholder partners (EBP trainers/intermediaries and funding agency representatives; *N* = 48 participants) in mixed-method data collection activities – surveys, focus groups, document review – to achieve consensus on the Fiscal Mapping Process steps while evaluating initial impacts on sustainment capacities (e.g., strategic planning, financial stability) and outcomes (e.g., intentions to sustain PCIT/TF-CBT).

**Findings:** Initial recruitment was challenging, but we successfully engaged 12 service agencies by leveraging their relationships with intermediary organizations that provided training/consultation in PCIT or TF-CBT; this represents an important early lesson learned. For this presentation, we anticipate having finished training in the Fiscal Mapping Process; several months of coaching; one round of survey data collection; and potentially some initial focus groups and document review. The presentation will describe the Fiscal Mapping Process prototype and pilot-testing agencies, and detail initial feedback and modifications made to the tool during the early months of pilot-testing.

**Implications for D&I Research:** This pilot-test will produce a Fiscal Mapping Process that builds behavioral health service agencies’ capacities to sustain funding for EBPs in coordination with stakeholders. We are already gaining insights into how the process and outcomes of Fiscal Mapping unfold within behavioral health service systems, including interactions with other implementation activities (e.g., the benefits of aligning Fiscal Mapping with trusted EBP training/consultation initiatives).

**Primary Funding Source:** National Institutes of Health

### S58 A multiple case study of the collaborative organizational approach to selecting and tailoring implementation strategies (COAST-IS)

#### Rebecca Lengnick-Hall

##### Washington University in St. Louis, St. Louis, MO, USA

###### **Correspondence:** Rebecca Lengnick-Hall (rlengnick-hall@wustl.edu)

**Background:** The Collaborative Organizational Approach to Selecting and Tailoring Implementation Strategies (COAST-IS) is an implementation intervention that targets organizational leaders’ and clinicians’ ability to select and tailor implementation strategies that address their site’s needs. COAST-IS was piloted in a matched-pair cluster randomized pilot study of 8 organizations that were implementing trauma-focused cognitive behavioral therapy (TF-CBT), and was found to be acceptable, appropriate, feasible, and useful to leaders and clinicians. This multiple case study of four organizations that received COAST-IS provides an in-depth understanding of how organizations were guided through the process of Implementation Mapping to tailor strategies to their site-specific needs.

**Methods:** COAST-IS involved site-visits, 5 virtual educational sessions, and 12 coaching sessions which led leaders and clinicians through the Implementation Mapping process (e.g., identifying implementation outcomes, performance objectives, determinants, implementation strategies, and mechanisms). Detailed case summaries were created for each organization so that we could comprehensively review each case and identify similarities and differences across the cases. Data sources included agency websites, site visit notes and recordings, survey data (two time points), coaching session notes and recordings, and implementation plan documents.

**Findings:** Across the cases, there was variation in the number and nature of performance objectives and strategies discussed. Organizations 1 and 4 had a smaller number of objectives and strategies that primarily focused on continuing or refining existing activities. In contrast, Organizations 2 and 3 discussed a range of objectives and strategies that could affect TF-CBT implementation. Organizations 1 and 3 displayed positive group dynamics that reflected collegiality and psychological safety. Organization 2, however, experienced some discontent and group conflict. Finally, there was variation in terms of the degree to which site visit information and baseline data shed light on how coaching calls and implementation plans unfolded.

**Implications for D&I Research:** COAST-IS is an implementation intervention that shows promise for strengthening organizations’ capacity to implement and sustain evidence-based practices by improving their ability to tailor strategies effectively. This study demonstrates the problem of “one size fits all” approaches to implementation, and it illustrates a novel application of Implementation Mapping as a method for tailoring implementation strategies.

**Primary Funding Source:** National Institutes of Health

## Global Dissemination and Implementation Science

### S59 Characterizing adaptations to mobile phone delivery of the adolescent transition package (ATP) in Kenya using the framework for reporting adaptations and modifications to evidence-based implementation strategies (FRAME-IS)

#### Dorothy Mangale^1^, Alvin Onyango^2^, Cyrus Mugo^2^, Caren Mburu^2^, Janet Itindi^2^, Arianna Rubin-Means^1^, Irene Njuguna^2^, Dalton Wamalwa^2,3^, Bryan Weiner^1^, Grace John-Stewart^1,4^, Kristin Beima-Sofie^5^

##### ^1^University of Washington, Seattle, WA, USA; ^2^Kenyatta National Hospital, Nairobi, Kenya; ^3^University of Nairobi, Nairobi, Kenya; ^4^Box 359909, University of Washington, Seattle, WA, USA; ^5^3980 15th Ave NE, University of Washington, Seattle, WA, USA

###### **Correspondence:** Dorothy Mangale (dmangale@uw.edu)

**Background:**

The COVID-19 pandemic resulted in disruptions to routine HIV services for adolescents and youth with HIV (AYHIV). The Adolescent Transition To Adult Care for HIV-infected Adolescents (ATTACH) was a hybrid II cluster randomized trial testing the effectiveness and implementation of an in-person, healthcare worker-delivered disclosure and transition intervention – the adolescent transition package (ATP). We describe adaptations made to the ATP to allow for mobile phone delivery.

**Methods:**

We conducted continuous quality improvement (CQI) meetings twice monthly for three months with healthcare workers (HCWs) involved in mobile phone delivery of the ATP at 10 intervention sites. CQI meetings facilitated by study staff used plan-do-study-act (PDSA) cycles and were audio-recorded. Using the Framework for Reporting Adaptations and Modifications to Evidence-based Implementation Strategies (FRAME-IS), we drafted memos for each recording characterizing adaptations, adaptation targets, reasons, and nature of the changes.

**Findings:**

Across 10 sites, we identified 60 adaptations (24 unique) including: introducing scripts to streamline call content, collaborating with caregivers to schedule calls, assigning adolescents to specific HCWs, using community health volunteers to conduct home visits, increasing staffing, spreading content covered over several calls and repeating material. Overall, 37% (n=22) of adaptations were content-related (information shared), 42% (n=25) were context-related (how phone delivery was implemented), and 8% (n=5) were about evaluation of calls. Primary motivations for adaptation were to: 1) address unreachable AYHIV due to wrong numbers, network issues, or lack of phones (n=17); 2) enhance comprehension and retention of content (n=10); 3) improve ATP reach (n=6); 4) mitigate scheduling challenges (n=4); and 5) improve documentation (n=4). Regarding the nature of modifications, majority, 33% (n=20) added elements to improve phone delivery including scripts, interactive segments, and comprehension assessments; 5% (n=3) involved repeating elements; and 7% (n=4) involved refining. Five percent (n=3) of adaptations involved a drift from, then return to, phone delivery. After the adaptation period, unreachable AYHIV remained the most frequently recurring challenge and motivation for adaptations.

**Implications for D&I Research:**

Adaptation of mobile phone delivery of ATP was a feasible and effective way of addressing challenges with continuity of care for AYHIV during COVID-19. Modifications were primarily additive and frequently addressed the inability to reach clients.

**Primary Funding Source:** National Institutes of Health

### S60 A mixed methods evaluation of task-shifting in management of screen-positive women to reduce loss to follow-up in a cervical cancer prevention program in iquitos, Peru

#### Valerie Paz-Soldan^1^, Anne Rositch^2^, Batel Blechter^3^, Margaret Kosek^4^, Joanna Brown^5^, Rachel Morse^6^, Daniel Lenin de Cuadro Hidalgo^7^, Karina Gonzales Díaz^7^, Magaly Figueredo Escudero^7^, Graciela Meza^8^, Lita Carrillo Jara^7^, Henrry Daza Grandez^7^, Anna Kohler-Smith^7^, Jhonny Cordova Lopez^7^, Patti Gravitt^9^

##### ^1^Tulane University, Lima, Peru; ^2^Johns Hopkins Bloomberg School of Public Health, Baltimore, MD, USA; ^3^New York University School of Medicine, New York City, NY, USA; ^4^University of Virginia School of Medicine, Charlottesville, VA, USA; ^5^AB PRISMA, San Miguel, Peru; ^6^Lima, Peru; ^7^Iquitos, Peru; ^8^Universidad Nacional de la Amazonia Peruana, Iquitos, Peru; ^9^University of Maryland School of Medicine, Baltimore, MD, USA

###### **Correspondence:** Valerie Paz-Soldan (vpazsold@tulane.edu)

**Background:** The effectiveness of cervical screening and management (CSM) programs to reduce cervical cancer burden is a function of high screening coverage and appropriate management of women who screen positive. The standard of care management of screen-positive women in high-income countries (i.e., referral to colposcopy with(out) biopsy) has been difficult to implement in low/middle-income countries (LMICs) who report a high rate of loss to follow-up (LTFU). New WHO-recommended strategies of task-shifting management of screen-positive women to primary health facilities (PHFs) is a promising adaptation to increase completion of care.

**Methods:** The Proyecto Precancer used the Integrative Systems Praxis for Implementation Research (INSPIRE) methodology to understand multilevel barriers in managing screen-positive women in the public health system in Iquitos, Peru. The proportion of screen-positive women reaching the recommended follow-up visit was evaluated for 18 months pre- and up to 12 months post-implementation of the task-shifting strategy to decrease LTFU based on primary monitoring and evaluation data collected. In-depth interviews and focus group discussions were analyzed using (a) pathway analysis to define the health system context and (b) the Health Care Access Barriers Models to examine individual perceptions and beliefs. Group model building with multiple scenario analysis enabled knowledge transfer and participatory decision-making for program adaptation to reduce LTFU.

**Findings:** At study inception, only 32.6% (63/193) of screen-positive women had evidence of follow-up when referred to the hospital for colposcopy. Positive women indicated multiple attempts to follow-up, thwarted by reinforcing cognitive, structural, and financial barriers. Using scenario analyses and mixed methods data, a participatory decision was made to adopt HPV molecular testing for screening and manage all positive women with visual assessment and same day ablative treatment if eligible at PHFs. This task shifting intervention increased follow-up to 75.7% (457/604). Screen-positive women found the new screening strategy acceptable, recommending it as a routine service. Investigations into the remaining 25% LTFU in the new program are ongoing and will be presented.

**Implications for D&I Research:** Use of the INSPIRE participatory systems approach, which includes rapid cycle mixed methods research, enabled adoption of a feasible and acceptable strategy for decreasing loss to follow-up in delivery of complex care cascades in LMICs.

**Primary Funding Source:** National Institutes of Health

### S61 Evaluating the implementation of an mHealth intervention by community health workers in Togo, West Africa

#### Elissa Faro^1^, Jessica Haughton^2^, Désiré Dabla^3^, Maiti Peters^1^, Essodinam Miziou^3^, Dawkin's Kamara^3^, Etonam Sowu^3^, Hyacinthe Awizi^3^, Tsolegnagbo Kossi^3^, Kelly Lue^2^, Sébastien Osterrieth^3^, Amanda Singer^2^

##### ^1^University of Iowa Health Care, Iowa CIty, IA, USA; ^2^Integrate Health, Medway, MA, USA; ^3^Santé Intégrée, Kara, Togo

###### **Correspondence:** Elissa Faro (elissa-faro@uiowa.edu)

**Background:**

In many low- and middle-income countries (LMICs), health services fail to meet patient needs for quality care. In Togo, West Africa, 20,000-30,000 mothers and children die every year of treatable diseases. With fewer than 400 practicing medical doctors in the country, community health workers (CHWs) constitute the critical frontline of the healthcare system and have played an integral role in decreasing inequities in access to quality care. Mobile clinical decision support tools can support CHWs’ ability to accurately assess and diagnose health problems in the field, thereby improving care for treatable clinical conditions. Despite the growing literature on facilitators and barriers of mHealth interventions in LMICs, little data are published on the implementation of these tools, and even less on how mHealth tools impact patient experience. This study assesses both the implementation of CHWs’ use of the tool and patient experience.

**Methods:**

The Practical Robust Implementation and Sustainability Model (PRISM) framework structured the interview guide and defined domains for analysis. Semi-structured interviews were completed with 22 CHWs purposively sampled for maximum variability and 21 patients one-week post-visit in French and the local language, Konkomba. Qualitative data were analyzed using a rapid assessment process by a US and Togolese analysis team.

**Findings:**

CHWs reported feeling empowered and more confident in their consultations and patients reported comfort with CHWs’ use of the tool. Discrepancies between treatment guidance from the tool and in-person clinical training emerged as well as issues with implementation infrastructure such as referral tracking and follow-up and the inability to indicate medication stockouts. Reach, adoption, and implementation measures from tool usage data highlight variation between sites that, linked with the organizational and patient perspectives from the qualitative data, are being shared back with the team in real-time to adapt and improve implementation.

**Implications for D&I Research:**

Providing real-time, actionable data is critical for understanding and adapting the implementation of mobile clinical decision support tools. This research contributes to existing literature both by providing novel information about the patient perspective and illustrating a real-world example of the rapid integration of qualitative data for implementation of an mHealth intervention in a global context.

**Primary Funding Source:** Global Research Partnership Award

### S62 Formative research and protocol adjustments for the intravenous vs oral iron for iron deficiency anemia in pregnant nigerian women (IVON) trial

#### Nadia A. Sam-Agudu^1,2^, Opeyemi R. Akinajo^3^, Mobolanle Balogun^4^, Ochuwa A. Babah^5^, Aduragbemi Banke-Thomas^6^, Rachel A. Quao^5^, Adamu M. Mukhtar^7^, Hameed Adelabu^8^, Titilope Adeyemo^9^, Hadiza S. Galadanci^7,10^, Bosede B. Afolabi^3,5^

##### ^1^International Research Center of Excellence, Institute of Human Virology Nigeria, Abuja, Nigeria; ^2^Institute of Human Virology, University of Maryland School of Medicine, Baltimore, MD, USA; ^3^Department of Obstetrics and Gynaecology, Lagos University Teaching Hospital, Idi-Araba, Lagos, Nigeria; ^4^Department of Community Health and Primary Care, College of Medicine, University of Lagos, Idi-Araba, Lagos, Nigeria; ^5^Department of Obstetrics and Gynaecology, College of Medicine, University of Lagos, Idi-Araba, Lagos, Nigeria; ^6^London School of Economics and Political Science, London, United Kingdom; ^7^Africa Center of Excellence for Population Health and Policy, Bayero University, Kano, Nigeria; ^8^Department of Haematology and Blood Transfusion, Lagos University Teaching Hospital, Idi-Araba, Lagos, Nigeria; ^9^Department of Haematology & Blood Transfusion, College of Medicine, University of Lagos, Idi-Araba, Lagos, Nigeria; ^10^Department of Obstetrics and Gynaecology, Aminu Kano Teaching Hospital, Kano, Nigeria

###### **Correspondence:** Nadia A. Sam-Agudu (nsamagudu@ihvnigeria.org)

**Background:**

Anemia in pregnancy (AIP) affects nearly 50% of pregnant African women, and is a significant cause of maternal-infant morbidity and mortality. Intravenous (IV) iron has been proven effective in treating AIP, however it is not standard-of-care in Nigeria. The IVON randomized clinical trial is the first to evaluate IV vs oral iron for AIP in Nigeria. We conducted a formative assessment to evaluate for contextual determinants to inform trial design adjustments.

**Methods:**

This qualitative study was conducted at all 10 IVON study sites in Kano (North-West Zone) and Lagos (South-West Zone). The Consolidated Framework for Implementation Research (CFIR) informed stakeholder-participant identification and questionnaire design. Transcripts from focus group discussions (FGDs) and key informant interviews (KIIs) were organized with Atlas.ti v8. CFIR also informed our inductive-deductive framework analysis of the qualitative data.

**Findings:**

We conducted 12 FGDs and 53 KIIs among 47 pregnant women, 10 women with AIP, 61 healthcare workers, 24 matriarchs, 23 male partners, eight traditional birth attendants, six community leaders, four health facility managers, and 10 government officials/policymakers. The formative data captured contextual determinants and key trial design adjustments in four CFIR domains. Findings highlighted the potential for COVID-19 conspiracy theory-fueled threats of misinformation relating to IV iron as a new intervention, therefore a robust information script was planned for all pregnant women participating in the trial (1-Individual Characteristics). To address external influences on uptake (2-Outer Setting), pregnant women opined that it was important to provide clear information for their family members as well. To sustain a conducive implementation climate (3-Inner Setting), healthcare workers expressed their need for periodic training, while managers and policymakers submitted that for success of the (4) Implementation Process, stakeholders at all socio-ecological levels needed consideration.

**Implications for D&I Research:**

Key insights from this formative assessment have informed trial design adjustment. This is good practice for strengthening understanding of the role of context, and mechanisms, mediators, and moderators in influencing implementation outcomes. Issues such as the risk involved in initiating a novel intervention in a time of crisis may not have been recognized without a formative assessment. Thus, early stakeholder consultation and continued engagement throughout design and implementation is essential.

**Primary Funding Source:** Bill and Melinda Gates Foundation

### S63 An interrupted time series evaluation of monthly cervical cancer screening rates before and after implementation of an HPV-based screening method allowing for self-collected samples in iquitos, Peru

#### Patti Gravitt^1^, Anne Rositch^2^, Batel Blechter^3^, Margaret Kosek^4^, Graciela Meza^5^, Rachel Morse^6^, Jhonny Cordova Lopez^7^, Lita Carrillo Jara^7^, Javier Vasquez^7^, Anna Kohler-Smith^7^, Valerie Paz-Soldan^8^, Sarah Gilman^9^

##### ^1^University of Maryland School of Medicine, Baltimore, MD, USA; ^2^Johns Hopkins Bloomberg School of Public Health, Baltimore, MD, USA; ^3^New York University School of Medicine, New York City, NY, USA; ^4^University of Virginia School of Medicine, Charlottesville, VA, USA; ^5^Universidad Nacional de la Amazonia Peruana, Iquitos, Peru; ^6^Lima, Peru; ^7^Iquitos, Peru; ^8^Barranco, Tulane Health Office for Latin America, LIMA, MD, Peru; ^9^Washington, USA

###### **Correspondence:** Patti Gravitt (patti.gravitt@nih.gov)

**Background:** Updated cervical cancer screening guidelines from the WHO recommend using HPV DNA detection for primary screening, either with a screen-and-treat or a screen-triage-and-treat management strategy, based on a comprehensive review of the evidence. Few studies, however, have systematically evaluated the temporal impact on screening coverage and follow-up (i.e., reach and effectiveness) when de-implementing ineffective complex screen-and-referral programs and adopting HPV screen-and-treat programs.

**Methods:** The Proyecto Precancer used an interrupted time series (ITS) analysis to evaluate change in monthly screening coverage and follow-up in non-pregnant women aged 30-49 years in 17 primary health facilities (PHFs) in Iquitos, Peru serving ~20,000 screen-eligible women from January 2018 through February 2020 (ITS analysis through November 2020 to reflect COVID-19 disruptions are in progress). Reach (screening coverage) and effectiveness (follow-up with visual assessment and ablative treatment (VAT)) were monitored via paper-based screening registration digitalized to an electronic database. The effect of introducing HPV screening was estimated using an ITS analysis with Poisson regression, adjusted for seasonal variation by including Fourier terms and a scaling parameter to allow for over-dispersion of data. Frequency of screening and follow-up are compared in pre-implementation (01/2018-06/2019), HPV-VAT scale up (07/2019-10/2019), post-implementation (11/2019-03/2020), COVID-19 restriction (04/2020-06/2020), and post-COVID-19 return to routine service (07/2020-11/2020) periods.

**Findings:** Implementation was phased into the 17 PHFs from July 2019 - Nov 2019. Pre-implementation, 1533 women were screened by VIA at a stable rate of 85.2 women/month. Post-implementation, 1427 women were screened by HPV, a significant increase in reach (monthly increase incidence ratio=1.16 (1.01, 1.34)), peaking at 297.7 tests/month from 12/2019-02/2020. During COVID-19 restrictions, screening decreased to 11 women/month, returning to an average of 226 women/month (101% of target) after return to routine service delivery. Similarly, effectiveness (completion of care) increased post-implementation (66.7% vs 29.7% pre-implementation, p<0.0001), with average monthly VAT of 11 visits during HPV scale-up increasing to 38.6 visits post-implementation. VAT procedures were not performed during COVID-19 restrictions, but resumed to 38 visits/month after return to routine service delivery.

**Implications for D&I Research:** Interrupted time series represents a pragmatic implementation research study design to inform scale-up and sustainability, particularly for complex interventions delivered in complex adaptive systems.

**Primary Funding Source:** National Institutes of Health

### S64 Youth led implementation strategies to promote HIV self-testing in Nigeria with eric compilation

#### Karan Modi^1^, Dana Alshekhlee^2^, Chisom Obiezu-Umeh^3^, Ucheoma Nwaozuru^3^, Titilola Gbaja-Biamila^2,4^, David Oladele^4^, Callie Walsh-Bailey^5^, Stacey Mason^3^, Alexis Engelhart^6^, Adesola Musa^7^, Rhonda BeLue^3^, Hong Xian^8^, Oliver Ezechi^7^, Joseph Tucker^9^, Juliet Iwelunmor^3^

##### ^1^College for Public Health & Social Justice, Saint Louis University, Saint Louis, MO, USA; ^2^College for Public Health & Social Justice, Saint Louis University, Saint Louis, USA; ^3^Saint Louis University, Saint Louis, MO, USA; ^4^Clinical Sciences Department, Nigerian Institute of Medical Research, Lagos, Nigeria; ^5^Washington University in St. Louis, St. Louis, MO, USA; ^6^College for Public Health & Social Justice, St. Louis, USA; ^7^Nigerian Institute of Medical Research, Yaba, Lagos, Nigeria; ^8^Saint Louis University, Saint Louis, Nigeria; ^9^University of North Carolina at Chapel Hill, Chapel Hill,, NC, USA

###### **Correspondence:** Karan Modi (karan.modi@slu.edu)

**Background:**

Evidence on the effectiveness of HIV self-testing (HIVST) on increased testing coverage exists, but less is known about how to tailor implementation strategies to meet youth needs, and how this improves uptake of HIVST in a resource-limited setting.Our study uses participatory approaches to identify, refine and test strategies to optimize the implementation and sustainability of HIVST services led by and for Nigerian youth.We also describe implementation strategies to promote HIVST among Nigerian youth.

**Methods:**

Between 2018 and 2020, we organized four participatory activities to increase HIV testing services in Nigeria – an open call, a designathon, apprenticeship training bootcamp, and pilot feasibility trial.The open call solicited creative strategies to promote HIVST among Nigerian youth, then had experts evaluate them.The designathon brought together youth teams to further develop their HIVST service strategies into implementation protocols.Teams that scored highest on pre-determined criteria were invited to a four-week capacity building bootcamp.The five teams that emerged from the bootcamp pilot-tested their HIVST service strategies over a six-month period.We mapped and compiled a matrix of the specified youth-developed service strategies across the nine categories described by the Expert Recommendations for Implementing Change (ERIC) taxonomy of IS strategies.

**Findings:**

A total of 2,962 Nigerian youth participated in the four activities (2,403 for the open calls, 127 for the designathon, 45 for the bootcamp and 387 for the pilot trial). From the ideas presented, a total of five youth-led interventions were conducted at select study sites and mapped under the ERIC framework. Ways in which these interventions crossed lines with the ERIC framework were engaging consumers (utilizing local idea submissions); using evaluative and iterative strategies (auditing and providing feedback on each implementation); adapting and tailoring to the context (tailoring strategies and promoting adaptability); developing stakeholder interrelationships (informing local opinion leaders); supporting clinicians (developing resource sharing agreements); providing interactive assistance (providing local technical assistance and facilitation); training and educating stakeholders (conducting continuous training).

**Implications for D&I Research:**

Findings will potentially add to the limited “how to do it literature” on implementation science strategies in a resource-limited setting and among a youth population traditionally underrepresented in implementation science literature.

**Primary Funding Source:** National Institutes of Health

### S65 Assessing the sustainability of the systems analysis and improvement approach to increase HIV testing in family planning clinics in mombasa, Kenya: Results of a cluster randomized trial

#### Jessica Long^1^, McKenna Eastment^1^, George Wanje^2^, Barbra Richardson^3^, Emily Mwaringa^4^, Kenneth Sherr^5^, Ruanne Barnabas^1^, Kishorchandra Mandaliya^1^, Walter Jaoko^6^, R. Scott McClelland^1^

##### ^1^University of Washington, Seattle, WA, USA; ^2^Ganjoni Clinic, Mombasa, Kenya; ^3^Fred Hutchinson Cancer Research Center, Vaccine and Infectious Disease Division, Seattle, WA, USA; ^4^Mombasa County Department of Health, Mombasa, Kenya; ^5^Global Health, University of Washington, Seattle, WA, USA; ^6^University of Nairobi, Nairobi, Kenya

###### **Correspondence:** Jessica Long (jesslong@uw.edu)

**Background:** In Kenya, 6.6% of women are living with HIV, with highest incidence among reproductive age women. A key HIV mitigation strategy is integration of HIV testing and counseling (HTC) into family planning services, but successful integration remains low. We conducted a cluster-randomized trial using the Systems Analysis and Improvement Approach (SAIA) to identify and address bottlenecks in HTC integration in family planning clinics in Mombasa County, Kenya. This trial was designed to 1) assess the efficacy of this approach, and 2) examine if SAIA could be sustainably incorporated into Department of Health (DOH) programmatic activities. In study Stage 1, SAIA was effective at increasing HTC uptake. Here we present Stage 2, which assessed if observed improvements in integrated HTC uptake would be sustained when implemented by the Mombasa County DOH with minimal study staff support.

**Methods:** This cluster-randomized trial was conducted in 24 family planning clinics in Mombasa County with 1:1 randomization to either the SAIA implementation strategy or standard care. In Stage 1, study staff conducted monthly SAIA visits and collected HTC data for 12 months. In Stage 2, we transitioned SAIA implementation to DOH staff, and compared HTC in intervention versus control clinics one year post-transition. Study staff provided training and minimal support to DOH implementers, and collected quarterly research data on HTC provision.

**Findings:** Only 31% (45/144) of planned SAIA visits were completed, largely due to the COVID-19 pandemic and a prolonged healthcare worker strike. In the final study quarter, 60.5% (118/195) of new family planning clients received HIV testing in intervention clinics, compared to 18.8% (45/240) in control clinics (prevalence rate ratio [PRR]=3.23, 95% confidence interval [CI]=2.29-4.55). HIV counseling was conducted with 81.6% (160/196) of new clients at intervention facilities compared to 22.4% (55/245) in control facilities (PRR=3.64, 95% CI=2.68-4.94).

**Implications for D&I Research:** Intervention clinics demonstrated sustained improvement in HTC under DOH leadership and implementation, even in the context of wide-scale healthcare disruptions and low fidelity to the SAIA implementation strategy. These finding suggest that systems interventions may be successful when integrated into DOH programmatic activities. Ongoing interviews with family planning clinic staff will provide valuable insight into determinants of sustainability.

**Primary Funding Source:** National Institutes of Health

### S66 Stakeholder engagement to identify and adopt acceptable, feasible, and sustainable strategies for cervical cancer prevention

#### Anne Rositch^1^, Patti Gravitt^2^, Rachel Morse^3^, Graciela Meza^4^, Lita Carrillo^5^, Joanna Brown^6^, Magdalena Jurczuk^3^, Margaret Kosek^7^, Valerie Paz-Soldan^3^

##### ^1^Johns Hopkins Bloomberg School of Public Health, Baltimore, MD, USA; ^2^University of Maryland School of Medicine, Baltimore, MD, USA; ^3^Tulane University School of Public Health and Tropical Medicine, New Orleans, LA, USA; ^4^Universidad Nacional de la Amazonia Peruana, Iquitos, Peru; ^5^Dirección de Atención Integral de Salud, Dirección Regional de Salud de Loreto, Iquitos, Peru; ^6^AB PRISMA, San Miguel, Peru; ^7^University of Virginia School of Medicine, Charlottesville, VA, USA

###### **Correspondence:** Anne Rositch (arositch@jhu.edu)

**Background:** Broad adoption, implementation and scale-up of HPV-based screening programs is essential for accelerating cervical cancer control in response to the WHO elimination goals. This requires multilevel stakeholder engagement to ensure acceptability, feasibility and sustainability of proposed changes to screening programs.

**Methods:** We undertook a group model building (GMB) strategy for stakeholder engagement in a process of co-creation for cervical cancer prevention in Iquitos, Peru. Through four ‘design workshops’, we shared visual mental models of the current screening system derived through process mapping to understand the current system, norms, and structures. Problems were identified through a facilitated deliberative dialogue, which enabled bi-directional knowledge transfer of internal system behavior and external evidence-based practice. A scenario analysis tool was used, where assumptions on screening and follow-up test performance/treatment efficacy and participation in each step of the care cascade could be modified. The impact of alternative programmatic choices was evaluated based on relative acceptability, feasibility, and sustainability.

**Findings:** Over 90 stakeholders participated in the GMB process, across multiple sectors of the health system. Several core barriers in the current system were identified, including system fragmentation, low coverage, high loss-to-follow-up, and inadequate training. Scenario analysis facilitated a shared decision that HPV testing would be more feasible and acceptable by increasing coverage due to the self-sampling option, increasing test sensitivity, and requiring less training compared to VIA. Acknowledging a limited capacity for colposcopy, a decision was made to adopt a screen-and-treat strategy using portable thermal ablation at the primary health facilities, which would decrease loss-to-follow-up by making the process simpler and more accessible to women.

**Implications for D&I Research:** Engaging a broad representation of stakeholders involved in the planning and delivery of cervical cancer control programs resulted in a shift in the screening paradigm and an enduring commitment to increase screening and treatment coverage.

**Primary Funding Source:** National Institutes of Health

## Health Policy Dissemination and Implementation Science

### S67 Funder's policies and understanding of implementation science play a role in the development of global implementation science

#### Ana Cardoso-Weinberg^1^, Chris Alley^2^, Linda Kupfer^3^, Garry Aslanyan^4^, Michael Makanga^5^, Fabio Zicker^6^, Ole Olesen^7^

##### ^1^European & Developing Countries Clinical Trials Partnership, EDCTP, The Hague, The Netherlands, The Hague, Netherlands; ^2^Hunter College, NY, NY, USA; ^3^Fogarty International Center, National Institutes of Health, Bethesda, MD, USA; ^4^World Health Organization, Geneva, Switzerland; ^5^European & Developing Countries Clinical Trials Partnership, EDCTP, The Hague, Netherlands, the Hague, Netherlands; ^6^Fundação Oswaldo Cruz (Fiocruz), Rio de Janeiro, Brazil, Rio de Janeiro, Brazil; ^7^European Vaccine Initiative (EVI), Heidelberg, Germany & Global Health Section, Department of Public Health, University of Copenhagen, Denmark., Copenhagen, Denmark

###### **Correspondence:** Garry Aslanyan (aslanyang@who.int)

**Background:**

Implementation research (IR) is an emerging research paradigm that helps research and public health programs achieve impact at scale. Supporting and developing this field is particularly important in low- and middle-income countries (LMICs) where the need to knowhow to implement effective interventions can be lifesaving. We share health research funders’ experiences with the policy of funding IRactivities in LMICs. This includes recommendations on policy level approaches to consider for successful investments in this field.

**Methods:**

Between May and September 2019, we conducted semi-structured interviews with 28 technical managers in charge of IR programmes in 23 research funding agencies which were supporting, have supported, or were interested in supporting IR in LMICs. The findings and discussions were consolidated into key themes.

**Findings:**

We were able to identify and group the funders’ strategies into seven approaches that are considered important by funders, at the policy level, when supporting IR in LMICs. If followed, these policies can help move the field of D&I forward globally:

(1) involve all relevant stakeholders from the outset and throughout the entire research and delivery process; (2) embrace and leverage the diversity of IR funders; (3) increase awareness and readiness to implement new IR knowledge ; (4) consider partnership building as central to IR, especially atthe start; (5) promote prioritization of capacity building for IR within funding agencies and in the extramural community; (6) create and communicate clear funding criteria for IR; (7) address sustainability by ensuring that IR skills and knowledge are embedded in national academic and health systems.

**Implications for D&I Research:**

Our findings corroborate that health research funding agencies are interested in supporting IR and point to opportunities for shaping a global research agenda for IR. The study findings offer broad direction and offer policy level guidance to funding agencies and related partner organizations on important elements to consider when funding and implementing IR in LMICs. Ultimately, this work could help improve the way that funders support global D&I research investments.

**Primary Funding Source:** WHO, NIH, EDCTP

### S68 Designing and implementing state-level fertility preservation health insurance benefit mandates

#### Sara McMenamin^1^, Ricardo Flores^1^, Sara Yoeun^1^, Omar Mesina^1^, Bonnie Kaiser^1^, Irene Su^2^

##### ^1^UC San Diego, La Jolla, CA, USA; ^2^UC San Diego ACTRI Dissemination and Implementation Science Center, La Jolla, CA, USA

###### **Correspondence:** Sara McMenamin (smcmenamin@ucsd.edu)

**Background:** Nearly 70,000 people aged 0 to 39 are diagnosed with cancer annually and thus experience higher risks of infertility due to cancer treatments. Infertility risks can be reduced by the evidence-based practice of fertility preservation (FP) care. Thus, state-level insurance benefit mandates for FP care have been passed in recent years to increase access to and utilization of these services. The goal of this research is to systematically characterize the variation in content and implementation of state-level FP health insurance benefit mandates and regulation and to provide stakeholders with guidance on best practices, gaps, and implementation needs.

**Methods:** Eleven states that had passed fertility preservation legislation as of March 22, 2021 were identified. For each state, final versions of legislative text from the state legislature’s website and implementation guidance from the insurance regulators’ website were obtained. Both the legislative text (n=11) and regulator documents (n=10) were uploaded to MAXQDA 2020 and were coded by two researchers based on the Exploration, Preparation, Implementation, Sustainment (EPIS) implementation science framework. Data were summarized by theme (i.e., code), with structured comparison of each theme across states.

**Findings:** On average, states took 223 days to implement their mandates from the start of the laws’ enactment dates to their corresponding effective dates, and a majority issued regulatory guidance after the law was in effect. Federal policies impacted state level implementation, with the ACA and HIPAA guiding design of fertility preservation benefits. In addition, a majority of states referenced medical society evidence-based clinical practice guidelines in the design of FP mandated benefits.

**Implications for D&I Research:** Our policy scan documented significant variation in the design and implementation of health insurance benefit mandates for FP services. Future considerations for policy development include specificity and flexibility of benefit design, reference to external evidence-based clinical practice guidelines to drive benefit coverage, inclusion of Medicaid populations in required coverage, and consideration of interaction with relevant state and federal policies. In addition, key considerations for implementation include the sufficient length of time for the implementation period, regulator guidance issued prior to the law going into effect, and explicit allocation of resources for the implementation process.

**Primary Funding Source:** Moores Cancer Center and UCSD Academic Senate Pilot Grants

### S69 Waiver of the ryan haight act and implementation of buprenorphine treatment at syringe service programs

#### Barrot H Lambdin^1^, Ricky Bluthenthal^2^, Hansel Tookes^3^, Lynn Wenger^4^, Paul LaKosky^5^, Alex Kral^4^

##### ^1^RTI International, Berkeley, CA, USA; ^2^University of Southern California, Los Angeles, CA, USA; ^3^University of Miami, Miami, FL, USA; ^4^RTI International, San Francisco, CA, USA; ^5^North American Syringe Exchange Network, Tacoma, WA, USA

###### **Correspondence:** Barrot H Lambdin (blambdin@rti.org)

**Background:** In the United States (US), 18% of people with an opioid use disorder (OUD) are receiving treatment. Buprenorphine, a treatment approved for nearly 20 years, reduces OUD and opioid overdose mortality. In addition, major disparities in buprenorphine treatment access have emerged among people who are African American, lack private insurance or have lower income. The Ryan Haight Act is a federal law that has regulated buprenorphine delivery, requiring an in-person examination with a provider before buprenorphine treatment initiation. At the beginning of the COVID-19 pandemic, federal agencies waived in-person examination requirements for buprenorphine treatment initiation, and states followed with emergency orders. We assessed whether the waiver of the Ryan Haight Act improved implementation of buprenorphine treatment at syringe service programs (SSPs) throughout the US.

**Methods:** We surveyed all known SSPs operating in the US in 2021. Out of the 431 SSPs, 325 (75%) responded to the online survey. We utilized logistic regression to assess whether the availability of on-site buprenorphine treatment initiation at SSPs changed after the Ryan Haight Act waiver in March 2020, compared to 2019, and which outer context factors were associated with implementing on-site buprenorphine.

**Findings:** In 2020, 29% of SSPs were implementing buprenorphine treatment, with 23% offering buprenorphine treatment via telehealth. After the Ryan Haight Act waiver, the odds of buprenorphine implementation within SSPs increased 60% (p=0.018). Regarding organizational characteristics, SSPs that were standalone non-profit organizations (aOR 3.45; p=0.001) or part of a larger non-profit organization (aOR: 8.63: p<0.001) had a higher odds of buprenorphine implementation, compared to those that where part of local public health department. In addition, SSPs with larger annual budgets were more likely to implement buprenorphine (aOR: 1.36 per quartile; p=0.032).

**Implications for D&I Research:** Our findings suggest that de-implementation of certain federal and state policies can remove key barriers to implementing evidence-based interventions. State adoption of these changes will be key as state and local emergency orders expire and prescribing potentially becomes more prescriptive. Increasing buprenorphine implementation within SSPs is critical as people who use drugs already engage with and trust these organizations to care for their health.

**Primary Funding Source:** National Institutes of Health

### S70 Examining system-wide implementation of new flexibilities to the national school lunch program

#### Sarah Moreland-Russell^1^, Jason Jabbari^2^, Louise Farah Saliba^3^, Daniel Ferris^4^, Tyler Frank^2^, Jessica Gannon^1^, Rebekah Jacob^3^, Emily Jayne^5^, Eliot Jost^1^, Yung Chun^6^

##### ^1^Prevention Research Center, Washington University in St. Louis, St. Louis, MO, USA; ^2^Washington University in St. Louis, Saint Louis, MO, USA; ^3^Brown School, Prevention Research Center, Washington University in St. Louis, St. Louis, MO, USA; ^4^Washington University in St. Louis, St. Louis, MO, USA; ^5^Washington University in St. Louis, St. Louis, USA; ^6^Washington University, St. Louis, MO, USA

###### **Correspondence:** Sarah Moreland-Russell (Smoreland-russell@wustl.edu)

**Background:** The National School Lunch Program (NSLP) is a complex system involving food suppliers, school food service directors, and student consumers. Policy changes impact this system and can result in disruptions, inefficient implementation, and ultimately patterns of youth food consumption that diverge from the intended goals of policy. A significant change to NSLP guidelines occurred in 2018 when food quality standards were relaxed, allowing flexibilities to school nutrition standards for milk, whole grains and sodium. Understanding how this change affected implementation of the program within the system informs future directions for guideline roll out.

**Methods:** We employed a mixed method approach to understand how recent policy impacted the school food system. Interviews were conducted between January –March 2021 with nine key representatives (3 manufacturers, 3 schools, and 3 USDA staff). We also surveyed a sample (N=118) of Missouri schools to determine implementation of flexibilities. Finally, we merged survey data with school-level meal count data from the Missouri Department of Elementary/Secondary Education from years 2015/16-2019/20. We then used OLS regression to examine how flexibility adoption related to the number of meals served.

**Findings:** Most (87%) survey respondents adopted at least one of the flexibilities, with whole grain (82%) and flavored milk (81%) flexibilities being the most common, followed by sodium (51%) . The most common reasons to adopt were to serve more food (74%) and meet student preferences (72%). Leveraging the time of policy adoption and controlling for changes in school meal enrollment across years, we found adoption of flexibilities was associated with roughly 326 (SE = 35; p-value < 0.001) more lunches being served per month. Manufacturer interviewees shared time to implement was a major challenge (requiring at least 18 months for product development and testing). Schools shared they often had inadequate supply of high demand products.

**Implications for D&I Research:** This study reviews policy implementation across an entire system including those making the policy to those implementing the policy across multiple levels. ***To inform more effective, efficient, and equitable policy development and implementation, it is imperative to understand policy implementation systems. This study provides an example on system-wide incorporation of multiple perspectives using mixed methods.***

**Primary Funding Source:** National Institutes of Health

### S71 Application of the epis framework to state-level tobacco cessation policy implementation: California Medicaid managed care plans

#### Sara McMenamin^1^, Melina Economou^1^, Bonnie Kaiser^1,2^, Sara Yoeun^1^, Erika Crable^2,3,4^

##### ^1^UC San Diego, La Jolla, CA, USA; ^2^UC San Diego ACTRI Dissemination and Implementation Science Center, La Jolla, CA, USA; ^3^Child and Adolescent Services Research Center, San Diego, CA, USA; ^4^University of California, San Diego, La Jolla, CA, USA

###### **Correspondence:** Sara McMenamin (smcmenamin@ucsd.edu)

**Background:** In 2016, the California Department of Healthcare Services (DHCS) released All Plan Letter (APL) 16-014 to its Medicaid managed care plans (MCPs) to provide guidance on implementing evidence-based practices related to tobacco-cessation. The aim of this study is to explore the barriers and facilitators to fidelity implementation of APL policy among California Medi-Cal MCPs using the Exploration, Preparation, Implementation, Sustainment (EPIS) framework.

**Methods:** From fall 2018 through spring 2019, data were collected via semi-structured interviews with MCP health educators (n=24) to assess fidelity of MCP tobacco-cessation policy implementation. Interviews were recorded, transcribed, and reviewed to develop initial themes related to barriers and facilitators to implementation of the APL. Thematic summaries were developed, discussed among the research team, and mapped onto EPIS constructs.

**Findings:** APL 16-014 (i.e., the innovation) was described as lacking clarity and specificity in its guidelines, hindering implementation. Related to the inner context, MCPs described the APL as beyond the scope of their resources, pointing to their own lack of educational materials, human resources, and poor technological infrastructure as implementation barriers. Within the Outer Context, MCPs identified a lack of incentives for providers and beneficiaries to offer and participate in tobacco-cessation programs, respectively. A lack of communication, educational materials, and training resources between the state and MCPs (i.e., missing bridging factors) were barriers to preventing MCPs from identifying smoking rates or gauging success of tobacco-cessation efforts. Facilitators included several MCPs collaborating with each other and using external resources to promote tobacco cessation. Additionally, a few MCPs used fidelity monitoring staff as bridging factors to facilitate provider training, track providers’ identification of smokers, and follow up with beneficiaries participating in tobacco-cessation programs.

**Implications for D&I Research:** The release of the evidence-based APL by DHCS was an important step forward in promoting tobacco-cessation services for Medi-Cal MCP beneficiaries. Improved communication on implementation among tobacco control stakeholders and improved bridging factors such as incentives for providers and patients are needed to fully realize policy goals.

**Primary Funding Source:** Tobacco-Related Disease Research Program

### S72 Scope, nature, and timing of research and non-research evidence use by policy advocates and its influence on the implementation of evidence-based policies

#### Itzhak Yanovitzky, William Bejarano, Calandra Lindstadt, Matthew Weber

##### Rutgers University, New Brunswick, NJ, USA

###### **Correspondence:** Itzhak Yanovitzky (itzhak@rutgers.edu)

**Background:** Research suggests that advocacy organizations are critical to the dissemination and adoption of evidence-based health policies and practices. However, less is known about their role, if any, in policy implementation. We report preliminary findings from an ongoing research project that tracks, compares and assesses mental health advocates and other stakeholders’ use of research and non-research evidence to influence the statewide implementation of universal screening for adolescent depression in schools as recommended by the U.S. Preventive Services Task Force.

**Methods:** The scope, nature, and timing of advocates’ research and non-research evidence use in this context was assessed using a mix of qualitative and quantitative analyses of data obtained from coding a collection of relevant policy documents (N=91), news stories (N=213), public statements (N=27), and social media posts (N=305,646) – all venues that policy advocates utilize for policy implementation-relevant inputs. Additionally, key-informant interviews (N=15) were conducted with policy advocacy directors of major stakeholder groups (mental health advocates, professional associations, teacher unions, etc.) to probe the goals and intended use of research and non-research evidence as well as about additional (informal) venues of engaging with policy implementers.

**Findings:** Across all inputs, references to research evidence are scarce and mostly describe national estimates of rates of depression in youth. Primarily anecdotal or experience-based evidence is presented to justify a position on specific provisions of planned implementation. Policy advocate interviews consistently found lack of implementation-relevant research (e.g., cost, access, needed resources and training, etc.) as a major constraint on research evidence use. The findings also suggest at least three different goals/strategies of using evidence in this context: to educate/enlighten, pressure, and/or negotiate with decisionmakers. In general, use of research evidence to educate/enlighten is preferred early in the process of deliberating policy implementation, whereas pressure and negotiation-driven use is more common as the decisionmaking process nears conclusion.

**Implications for D&I Research:** There is an acute shortage of implementation-relevant research that can inform sound and successful implementation of evidence-based health policies. Equipping policy advocates and other implementation stakeholders with research that is directly relevant to barriers and facilitators to implementation can greatly facilitate the adoption and adaption of evidence-based policies and practices.

**Primary Funding Source:** The William T. Grant Foundation

### S73 Optimizing frameworks to advance policy d&I science: Examples and recommendations

#### Erika Crable^1,2,3^, Rebecca Lengnick-Hall^4^, Nicole Stadnick^2,3,5^, Joanna Moullin^6^, Gregory Aarons^1,2,3^

##### ^1^University of California, San Diego, La Jolla, CA, USA; ^2^Child and Adolescent Services Research Center, San Diego, CA, USA; ^3^UC San Diego ACTRI Dissemination and Implementation Science Center, La Jolla, CA, USA; ^4^Washington University, St. Louis, MO, USA; ^5^University of California San Diego, La Jolla, CA, USA; ^6^Curtin University, Perth, WA, Australia

###### **Correspondence:** Erika Crable (ecrable@health.ucsd.edu)

**Background:** Policy is instrumental in influencing healthcare access, quality, and patient outcomes, yet the complex processes of health policy D&I are understudied. One reason for this is that few theories, frameworks, or models specifically guide policy D&I research. This presentation defines the goals of policy D&I research and describes key recommendations for optimizing D&I frameworks to investigate policy D&I determinants and processes. The Exploration, Preparation, Implementation, Sustainment (EPIS) framework is used to demonstrate the application of each recommendation because of its seminal, flexible nature, although recommendations may be applied when using other D&I frameworks.

**Methods:** We conducted a narrative review to identify examples of policy D&I research that used EPIS and were published between 2011-2021. Articles that investigated ‘Big P’ (e.g., federal/state) and ‘little p’ (e.g., organizational) policy implementation efforts were examined to extract examples of policy characteristics and goals, the focal evidence-based practice, breadth/depth of EPIS construct use and adaptations. Five D&I scientists experienced in framework development and application reviewed the extracted data and engaged in consensus decision-making to develop recommendations for optimizing frameworks for policy D&I research.

**Findings:** 59 articles employed EPIS to investigate policy D&I and served as examples to guide recommendation development. We provide 6 recommendations to advance policy D&I framework research: (1) Specify dimensions of a policy’s function (i.e., define the policy type, multi-level outer and inner contexts where policy exists, capital exchanged) and, (2) form [i.e., policy origin, structure (e.g., un/funded mandate), dynamism (e.g., competing/supporting policies, politics, timing), outcomes (e.g., improve quality, equity, reduce costs)]. (3) Describe non-linear D&I phases, (4) temporal roles of diverse stakeholders across contexts, (5) policy-relevant inner/outer context adaptations (e.g., stigma, media), and (6) bridging factor functions and forms that promote policy D&I across contexts.

**Implications for D&I Research:** Policy D&I is an emergent research area which should be leveraged to increase the use of evidence in health policy and to investigate downstream health outcomes related to policy change. We developed 6 framework recommendations to optimize existing D&I frameworks rather than introducing new untested models. Framework recommendations provide researchers with a needed tool to advance policy D&I research methods.

### S74 From bench to bill: Institutional reforms to facilitate the use of non-partisan research evidence use in California state health policymaking

#### Neda Ashtari^1^, Elizabeth Barnert^1^, Justin Abbasi^2^

##### ^1^David Geffen School of Medicine at UCLA, Los Angeles, CA, USA; ^2^David Geffen School of Medicine, Los Angeles, CA, USA

###### **Correspondence:** Neda Ashtari (nashtari@mednet.ucla.edu)

**Background:** Bridging the translational gap between non-partisan research evidence and health policy in state legislatures requires understanding the systematic barriers to non-partisan research evidence use. Previous studies present limited perspectives on institutional-level barriers to non-partisan research evidence use in health policymaking that operate within specific state legislatures. Through interviews with California state legislators, legislative staff, and support staff, we sought to identify institutional-level barriers to and solutions for enhancing non-partisan research evidence use in health policymaking.

**Methods:** We conducted semi-structured interviews with 24 California state legislators, legislative staff, and support staff. Using purposive sampling, we invited members of the Senate and Assembly Health Committees to participate via email or in-person (92% response rate). Interviews explored stakeholder role, institutional-level barriers to non-partisan research evidence use, and potential institutional-level solutions. We performed thematic analysis of interview transcripts to identify emergent barriers and solutions.

**Findings:** Institutional barriers to non-partisan research evidence use were grouped into the following concepts: Accessibility, Bias, and Capacity (ABCs). Specifically, participants described: limited accessibility of non-partisan research evidence, bias among institutional knowledge-brokers, and insufficient capacity to utilize non-partisan research evidence due to time and resource constraints. Institutional barriers to non-partisan research evidence use increase the legislature’s reliance on partisan knowledge-brokers and reduce evidence use overall. Institutional reforms may improve the dissemination and use of non-partisan research evidence to bridge the “know-do” gap in health policymaking.

**Table 1 (abstract S74).** Institutional barriers and facilitators of non-partisan research evidence use in California state health policymaking

Barriers	Effect	Potential solutions
Lack of *access* to non-partisan research evidence	Overreliance on lobbyists and decreased evidence use	Update data-sharing systems Increase access to peer-reviewed research Connect academia and the legislature
*Bias* among institutional knowledge-brokers	Distrust of research evidence provided	Adopt conflict-of-interest policies Increase non-partisan: partisan staff ratio Create non-partisan review bodies Reform committee structure and practices
Low *capacity* to utilize non-partisan research evidence	Overreliance on lobbyists and decreased evidence use	Limit bill introductions Reform deadline systems Expand legislative workforce

**Implications for D&I Research:** Institutional changes to increase the use of non-partisan research evidence in state health policymaking may enable legislators to enact policies that achieve better health outcomes, reduce expenditures, and advance health equity.

**Primary Funding Source:** National Institutes of Health

## Models, Measures, and Methods

### S75 From systematic reviews to systematic data platforms: Rapid cycle systems modeling to apply the literature on perinatal depression screening to implementation efforts

#### Katherine M. Cooper^1^, Leah Ramella^2^, Slawa Rokicki^3^, Wanlu Xu^1^, Esther Boama-Nyarko^1^, Grace Masters^1^, Nancy Byatt^4^, Thomas Mackie^2^, R. Chris Sheldrick5

##### ^1^University of Massachusetts Medical School, Worcester, MA, USA; ^2^SUNY Downstate Health Sciences University School of Public Health, Brooklyn, NY, USA; ^3^Rutgers School of Public Health, Piscataway, NJ, USA; ^4^University of Massachusetts Medical School - Worcester, Worcester, MA, USA; ^5^Boston University School of Public Health, Boston, MA, USA

###### **Correspondence:** R. Chris Sheldrick (rshldrck@bu.edu)

**Background:** Rapid Cycle Systems Modeling (RCSM) is an implementation strategy designed to actively engage stakeholders in simulation modeling to inform implementation decisions. One potential use of RCSM is to inform the building of the *adaptome*, described by Chambers and Norton (2016) as including “a common data platform to house systematically captured information about variations in delivery of evidence-based interventions across multiple populations and contexts.” To illustrate use of RCSM in building a common data platform, we use the case example of perinatal depression screening.

**Methods:** RCSM is a group process designed to assist stakeholders in using simulation models to examine underlying assumptions, consider alternative strategies, and anticipate downstream consequences of implementation. Each iteration of RCSM includes three steps: (1) identification of the primary research question, (2) development and review of the simulation model, and (3) evaluation of the model and the insights offered. Our team used RCSM to: (1) analyze published evidence on perinatal depression screening, initially focusing on implications of screening results for understanding prevalence; (2) conduct study-specific simulations to estimate prevalence, supplemented by estimates of sensitivity and specificity from published meta-analyses; and (3) evaluate initial simulation models for potential insights to inform further implementation and evaluation.

**Findings:** Highlighting the need for a common data platform, analysis revealed highly variable reporting of depression screening. However, initial rounds of RCSM identified key questions regarding underlying prevalence and 14 papers with data sufficient to support modeling. Simulation-based estimates of underlying prevalence display marked heterogeneity, including several implausible values. Evaluation of initial simulation models suggest that sensitivity and specificity are not stable properties of screening questionnaires, but instead that study-level differences (e.g., patients’ disclosure of symptoms) may play a role. Implications for potential variation in screener sensitivity and specificity were explored.

**Implications for D&I Research:** RCSM highlighted common (but not universal) metrics that could meaningfully inform a common data platform for perinatal depression screening. Further application of RCSM in perinatal depression will include: (a) dialogue with a wider circle of stakeholders to explore implications for equity, (b) extending simulation models to address a broader array of outcomes, and (c) development of common data elements for future screening studies.

**Primary Funding Source:** Patient-Centered Outcomes Research Institute

### S76 Pathways to pre-exposure prophylaxis (PrEP) implementation in family planning clinics: Application of configurational comparative methods

#### Kaitlin Piper^1^, Katherine Anderson^1^, Caroline Kokubun^1^, Anandi Sheth^2^, Jessica Sales^1^

##### ^1^Emory University, Rollins School of Public Health, Atlanta, GA, USA; ^2^Emory School of Medicine, Atlanta, GA, USA

###### **Correspondence:** Kaitlin Piper (kaitlin.piper@emory.edu)

**Background:**

Title X-funded family planning clinics have been identified as optimal sites for delivery of pre-exposure prophylaxis (PrEP) for HIV prevention. However, PrEP has not been widely integrated into family planning services, especially in the Southern U.S., and data suggest there may be significant implementation challenges in this setting. Configurational comparative methods (CCMs), based on Boolean algebra and set theory, are well-suited for identifying multiple pathways and conditions that lead to an outcome. We utilized CCMs to identify the pathways that lead to the successful implementation of PrEP in Title X-funded family planning clinics across the South.

**Methods:**

In 2018, we conducted in-depth qualitative interviews with key informants from 38 family planning clinics (11 clinics prescribed PrEP and 27 did not) across the Southern U.S. (Mid-Atlantic, Southeast, and Southwest regions). Clinics were selected based on key characteristics (e.g., clinic type, urban/rural, region) to enhance representation. Interviews were guided by constructs from the Consolidated Framework for Implementation Research (CFIR). Qualitative comparative analysis (QCA), a type of CCM, was utilized to uncover configurations of CFIR contextual factors that are key to PrEP implementation success in family planning clinics.

**Findings:**

We identified 3 distinct construct configurations, or “solution paths” that led to PrEP implementation in family planning clinics. These three paths collectively explained 100% of the PrEP-providing clinics with 100% consistency: (1) high “Leadership Engagement” AND high “Available Resources”; OR (2) high “Leadership Engagement” among clinics NOT located in the Southeast; OR (3) high “Access to Knowledge and Information” among clinics NOT located in the Southeast. Additionally, there were 2 solution paths that explained 96% of non-PrEP clinics with 100% consistency: (1) low “Access to Knowledge and Information” and low “Leadership Engagement”; OR (2) low “Available Resources” and high “Cosmopolitanism”.

**Implications for D&I Research:**

In the field of implementation science, there is a need for analytic methods that can capture the complexity and heterogeneity in implementation across organizations. To address this gap, CCMs, such as QCA, are specifically designed to examine combinations of explanatory factors that can lead to implementation. This method can pinpoint the most effective implementation-facilitators to promote future intervention uptake.

**Primary Funding Source:** National Institutes of Health

### S77 Developing innovative methodologies with a participatory approach to examine how the complex relationality between healthcare interventions, their contexts, and implementation strategies produce positive outcomes

#### Miriam Bender^1^, Marjory Williams^2^, Maricela Cruz^3^, Claude Rubinson^4^, Zahra Sharifiheris^5^

##### ^1^Sue & Bill Gross School of Nursing, University of California, Irvine, Irvine, CA, USA; ^2^Central Texas Veterans Health System, Veterans Health Administration, Temple, TX, USA; ^3^Kaiser Permanente Washington Health Research Institute, Seattle, WA, USA; ^4^University of Houston, Houston, TX, USA; ^5^University of California, Irvine, Irvine, CA, USA

###### **Correspondence:** Miriam Bender (miriamb@uci.edu)

**Background:** There is growing consensus that implementation and evaluation of complex healthcare delivery system interventions must move past traditional binary questions of efficacy and towards a more sophisticated examination of generalizable determinants of successful implementation and outcomes. Participatory approaches become critical as implementation science acknowledges the complexity and relationality involved in adopting care delivery interventions, in terms of heterogeneous contexts and implementation strategies utilized. Methods to produce knowledge about this heterogeneous relationality are needed, yet also need to fit the requirements of busy health system stakeholders. We report on a hybrid type II implementation-effectiveness study that was designed with a national-level participatory research collaborative to evaluate patterns of care delivery implementation that generate consistent improvements in care quality/safety outcome.

**Methods:** Approaches able to generate context-sensitive yet robust evidence about the causal relationships between care delivery implementation, practice, and outcomes include hybrid implementation-effectiveness research design, comparative case study methodology, interrupted time series (ITS) analysis, and qualitative comparative analysis (QCA). Survey and interview data allow for case comparisons of implementation strategies/success across national healthcare settings. ITS estimates outcome change point, change-point correlation structure and trajectory, and outcome variance pre-post implementation over time. QCA identifies necessary and sufficient CNL implementation configurations (using comparative case study findings) that achieve outcome effectiveness (using ITS findings).

**Findings:** Preliminary results demonstrate nationwide feasibility of study recruitment and data collection procedures, developed through participatory approaches meeting both research and health system needs. We’ve confirmed the sensitivity of our novel ‘Robust-ITS’ modeling approach to detect the empirical change point in measured outcomes as well as changes in outcome score variability pre-post implementation, which to our knowledge is the first study able to reliably quantify outcome consistency pre-post intervention, an important sustainability outcome.

**Implications for D&I Research:** Our research approach and methods show capacity to capture configurations, or ‘causal recipes’ of relational elements that cluster into patterns of care delivery implementation associated with positive outcomes. Importantly, different pattern clusters may achieve the same outcome, or vice versa, depending on the context and dynamics. Methods that can elucidate this contextual, relational knowledge will increase the chances for informed and successful implementation of promising yet complex care delivery interventions.

**Primary Funding Source:** Agency for Healthcare Research and Quality

### S78 Simulation of the effects of Medicaid policy levers on the implementation of smoking cessation programs

#### Chia-Hsiu Chang^1^, Tingting Ji^1^, Wanyu Huang^1^, Takeru Igusa^1^, Elizabeth Stuart^2^, Nae-Yuh Wang^1^, Emma McGinty^2^, Gail Daumit^3^

##### ^1^Johns Hopkins University, Baltimore, MD, USA; ^2^Johns Hopkins Bloomberg School of Public Health, Baltimore, MD, USA; ^3^Johns Hopkins University School of Medicine, Baltimore, MD, USA

###### **Correspondence:** Tingting Ji (tji8@jhu.edu)

**Background:** There are eight common Medicaid reimbursement policy options for smoking cessation (SC) programs that can influence implementation processes: copayment, prior authorization, required counseling for pharmacotherapy, stepped therapy restrictions, limits on duration, limits on smoking quit attempts, and reimbursement rates that can be tied to patient outcomes. Each option can result in multiple pathways of influence that can differentially impact implementation outcomes. There is a need for methods that can help policymakers more fully understand these pathways and make decisions appropriate for their constituents.

**Methods:** Agent-based modeling (ABM) is a simulation method that is being increasingly applied in implementation science. Agents are used to simulate the key actors in an SC program, including providers who deliver the therapies, their patients, and the clinic administrators who manage the treatment delivery and reimbursement processes. These simulated agents interact over time, following rules of behavior governed by treatment protocols, patient quitting behavior, which is calibrated using data from published studies of SC programs, and relevant reimbursement policies. SC programs for people with serious mental illness were used as the case study, because of the extremely high prevalence of tobacco use in this population.

**Findings:** What-if scenarios were simulated by switching on and off the various policy options described in the Background. The model predicted how each combination of policy options impacted implementation outcomes. For instance, limits on duration resulted in alterations of evidence-based practices, while stepped therapy restrictions affected the providers’ ability to select the SC program for each patient. An in-depth analysis of the financial impacts of outcomes-based and fee-for-service reimbursement policies indicated that the cost inefficiencies associated with fee-for-service were partially offset by patient-provider interactions. The model simulations indicated that this is due to increases in provider effort when the SC treatment shows significant promise of influencing patient smoking behavior.

**Implications for D&I Research:** ABMs can be used to simulate candidate policy options and their impacts on SC implementation outcomes of fidelity, feasibility, acceptability, and cost. These impacts can be observed at multiple levels and stages, including at the level of provider-patient interactions, providing valuable insights into pathways of policy influence.

**Primary Funding Source:** National Institutes of Health

### S79 Increasing access to organization theories for use in implementation science

#### Sarah Birken^1^, Jennifer Leeman^2^, Linda Ko^3^, Maria Fernandez^4^, Mary Wangen^5^, Hannah Arem^6^, Terry Huang^7^, Michelle Kegler^8^, Per Nilsen^9^, Prajakta Adsul^10^, Matthew Lee^11^

##### ^1^Wake Forest School of Medicine, Winston-Salem, NC, USA; ^2^School of Nursing, University of North Carolina, Chapel Hill, NC, USA; ^3^University of Washington, Seattle, WA, USA; ^4^The University of Texas Health Science Center at Houston School of Public Health, Houston, TX, USA; ^5^University of North Carolina, Chapel Hill, NC, USA; ^6^MedStar Health Research Institute, Hyattsville, MD, USA; ^7^The City University of New York, New York, NY, USA; ^8^Emory University, Atlanta, GA, USA; ^9^Linkoping University, Linkoping, Sweden; ^10^University of New Mexico, Albuquerque, NM, USA; ^11^NYU Grossman School of Medicine, New York, NY, USA

###### **Correspondence:** Sarah Birken (sbirken@wakehealth.edu)

**Background:** Organization theories offer implementation researchers numerous highly relevant, untapped explanations of the organizational dynamics underlying the implementation of evidence-based practices. The importance of using theory has increasingly been emphasized in implementation research; however, organization theories remain underused in the field. Frameworks that capture organizational constructs (e.g., environmental context and resources) exist (e.g., Consolidated Framework for Implementation Research; Theoretical Domains Framework), but their influence on implementation often remains a ‘black box.’ Limited understanding of how and why organizational constructs influence implementation is needed to improve implementation outcomes. To advance this understanding among implementation scientists, we summarized organization theories most relevant to implementation science.

**Methods:** We surveyed 18 subject matter experts to identify organization theories that they believed to be relevant to implementation science. From 62 key texts describing the theories, two investigators independently abstracted constructs and propositions regarding how or why they influence implementation, described the potential relevance of organization theories’ propositions for implementation, and summarized each theory in an abstraction form. The two investigators then met to reconcile discrepancies until they reached consensus. A third investigator reviewed reconciled abstraction forms for accuracy and completeness.

**Findings:** We identified nine organization theories with relevance for implementation science: contingency, complexity, institutional, network, organizational learning, resource dependence, sociotechnical, and transaction cost economics. From the theories, we abstracted 70 constructs and 65 propositions. Example constructs from institutional theory are coercive, mimetic, and normative pressures (e.g., accreditation standards; care improvement strategy zeitgeist). Example proposition: “Coercive, mimetic, and normative pressures cause organizations to become increasingly similar to each other.” The relevance for implementation is that we may leverage pressures from within an organizational field to promote uptake of evidence-based practices.

**Implications for D&I Research:** The completed abstraction forms are available on the Cancer Prevention and Control Research Network (CPCRN) website. We will teach CPCRN Scholars to use the forms with the goal of increasing knowledge and access to organization theories among an interdisciplinary audience of implementation scientists. Next steps include consolidating the organization theory constructs into domains and translating the resulting framework for use among policymakers and practitioners.

**Primary Funding Source:** Centers for Disease Control and Prevention

### S80 Unifying scale-up frameworks: A review and systems thinking model

#### Cole Hooley^1^, Katherine Marcal^2^, Mia Vogel^3^

##### ^1^Brigham Young University, Provo, USA; ^2^University of Nevada, Las Vegas, Las Vegas, USA; ^3^Washington University in St. Louis, Saint Louis, MO, USA

###### **Correspondence:** Cole Hooley (cole_hooley@byu.edu)

**Background:** Implementation science calls us to scale up effective interventions intentionally, rapidly, equitably, and sustainably. Successful scale-up requires working across multiple sectors. The literature presents several frameworks that identify key drivers of scale-up and important sectors to engage. These frameworks explicate the scale-up process and often indicate feedback inherent among constructs; however, frameworks do not explicitly articulate the dynamic complexity underlying scale-up efforts. Understanding scale-up drivers and the feedback patterns among them can support multi-sector scale-up success.

**Methods:** We applied a two-phase approach to address this gap in the literature. In the first phase, we applied a hermeneutic review approach to identify constructs from scale-up framework review articles indexed in Medline, CINAHL, PsycINFO, Embase, Web of Science, and Google Scholar. Two researchers reviewed the articles independently to confirm inclusion and resolved any discrepancies through consultation. Then two researchers extracted the constructs from the articles. We used thematic analysis to consolidate the constructs from across the frameworks. In the second phase of the study, we used system dynamics to iteratively construct a causal loop diagram (CLD) articulating causal links between scale-up constructs. This method aids in understanding dynamic interrelationships and how those relationships could impact successful scale-up. We built an initial CLD after reviewing the first wave of constructs. Then we iteratively folded in salient constructs postulating relationships among constructs with consultation from scale-up experts and extant literature.

**Findings:** The search yielded 915 articles; we included 22 articles in the extraction process, and 15 have been extracted. The resultant CLD consisted of 21 constructs and identified five reinforcing feedback patterns and three balancing feedback patterns. Key feedback processes emphasized the significance of equitable service delivery; timely, accurate dissemination of intervention benefits; and measures to overcome unintended consequences undermining sustainment of scale-up efforts.

**Implications for D&I Research:** The resulting interpretation and causal loop diagram unify divergent frameworks of scale-up, representing a novel use of system science methods with implications for improving the scale-up of effective interventions across diverse sectors.

**Primary Funding Source:** National Institutes of Health

### S81 Technology-based implementation: A case study for expanding common determinants frameworks

#### Geoffrey Barnes^1^, Emily Sippola^2^, Allison Ranusch^3^, Anne Sales^2^, Jeremy Sussman^4^

##### ^1^Univeristy of Michigan, Ann Arbor, MI, USA; ^2^University of Michigan, Ann Arbor, MI, USA; ^3^Center for Clinical Management Research, Ann Arbor, MI, USA; ^4^CCMR, Veterans Health Administration, Ann Arbor, MI, USA

###### **Correspondence:** Geoffrey Barnes (gbarnes@umich.edu)

**Background:** Frameworks are commonly used to guide the implementation process, identify critical barriers, and select strategies. However, current frameworks may not adequately address emerging factors. Specifically, the rise of technology in daily practice and the use of electronic health records represents areas where commonly used determinants frameworks may not adequately address important barriers to evidence-based care delivery.

**Methods:** We conducted semi-structured interviews with 45 anticoagulation clinic clinicians at 26 clinical sites across the United States about the barriers and facilitators to implementing or preparing to implement a population health dashboard for managing oral anticoagulant medications. Following a rapid qualitative analysis methodology, pre-existing codes corresponding to the Consolidated Framework for Implementation Research (CFIR) determinants framework and Technology Acceptance Model (TAM) were assigned to relevant text.

**Findings:** *Clinician authority and autonomy* emerged as a unique theme not captured in any existing CFIR constructs. *Clinician autonomy* to define their workflow and allocate effort was identified as a key facilitator of successfully implementing the dashboard. Furthermore, limited *authority* for dashboard users (nurses and pharmacists) to make clinical change themselves (e.g., without relying on a physician prescriber) was identified as a key barrier to implementation. Finally, we identified many complex themes around available time, personnel number and expertise, physical technology (e.g., computer, screens), and technological support availability that were coded within the CFIR construct for *available resources.* Notably, both *authority/autonomy* and *available resources* had minimal overlap with the various TAM constructs.

**Implications for D&I Research:** Our case example highlights that common implementation science determinant framework (e.g., CFIR, Theoretical Domains Framework) may not have enough specificity for technology-intensive implementation efforts. While use of technology-specific frameworks (e.g., TAM) can be beneficial, incorporation of other technology-related determinants into common determinant frameworks may help future implementation projects achieve success. This is of particular importance given that many population health tools leverage new technologies and non-physician clinicians to improve care delivery. Future work to update and expand on existing implementation strategies to better address technology-based implementation efforts could improve their utility in coming years.

**Primary Funding Source:** Agency for Healthcare Research and Quality

### S82 Introduction and application of the consolidated framework for implementation research (CFIR): Version 2 (CFIR V2)

#### Laura Damschroder^1,2^, Caitlin Reardon^3^, Marilla Widerquist^3^, Julie Lowery^4^

##### ^1^Center for Clinical Management Research, VA Ann Arbor Healthcare System, Veterans Health Administration, Ann Arbor, MI, USA; ^2^VA PROVE QUERI, Ann Arbor, MI, USA; ^3^VA Ann Arbor Healthcare System, Veterans Health Administration, Ann Arbor, MI, USA; ^4^VA Ann Arbor Center for Clinical Management Research, Veterans Health Administration, Ann Arbor, MI, USA

###### **Correspondence:** Laura Damschroder (laura.damschroder@va.gov)

**Background:**

Organizational context includes many active forces that work for or against implementation of innovations. Determinant frameworks help conceptualize, assess, and address contextual factors to increase the likelihood of successful implementation. The CFIR is among the most frequently cited determinant frameworks within implementation science and real-world settings. However, as implementation science has matured, gaps in the CFIR have been identified and updates are needed.

**Methods:**

We identified recommendations through a literature review of articles that mentioned the CFIR in the title and/or abstract. We surveyed corresponding authors of included articles, who involved their co-authors as appropriate. The survey elicited specific recommendations at the domain and construct levels and ratings of framework quality (e.g., clarity, relevance).

**Findings:**

We identified 377 articles; 130 (39%) of 334 unique corresponding authors responded to the survey. Of those who responded, 67% used the CFIR in more than one project, 83% used the CFIR within health services research, and 55% used the CFIR combined with another framework. Most respondents (>50%) affirmed 10 different quality ratings (e.g., the CFIR is applicable, useful). However, most respondents indicated that the CFIR was not (29%) or only partially (37%) easy for non-researchers to use.

The CFIR V2 will encapsulate changes based on recommendations from the literature and survey responses, including better centering patients, teams, and equity, as well as adding an Outcomes Addendum. Patients will be centered more prominently by adding *Patient-Centered Culture* to the Inner Setting and including *Patients* as a role on the implementation team. The key role of teams will be acknowledged by adding *Teamness* to the Inner Setting and *Teaming* to the Process Domain. Equity will be discussed by linking to the Health Equity Implementation Framework and by adding constructs, e.g., *Community Characteristics* to the Outer Setting, which includes consideration of racism/anti-racism in the community. Finally, an Outcomes Addendum will distinguish between CFIR contextual determinants versus patient determinants and between implementation versus innovation outcomes.

**Implications for D&I Research:**

As implementation science matures as a discipline, frameworks must mature too. The CFIR V2 includes significant improvements based on user recommendations.

**Primary Funding Source:** Department of Veterans Affairs

### S83 A stakeholder-engaged consolidated framework for implementation research: Opportunities to incorporate patient and frontline staff perspectives to improve implementation

#### Nadia Safaeinili

##### University of California, Berkeley, Berkeley, CA, USA

###### **Correspondence:** Nadia Safaeinili (nadiasaf@berkeley.edu)

**Background:** The Consolidated Framework for Implementation Research (CFIR) is widely used in implementation science, with demonstrated value in varied research and practice settings. It lacks, however, explicit attention to patient/end-user needs and domain-spanning constructs to capture team interdependencies impacting implementation. The purpose of this study is to highlight these gaps in CFIR constructs and demonstrate how we incorporate such perspectives in pragmatic CFIR applications.

**Methods:** We applied CFIR to three primary care-based quality improvement projects across various stages of evaluation design, data collection, and analysis: 1.“Primary Care 2.0,” a team-based care model redesign leveraging novel, co-located extended care team members (e.g. pharmacist, dietitian, mental health specialist); 2.“Humanwide,” a whole-person precision medicine pilot integrating digital health monitoring, genetic and pharmacogenomic testing, and tailored health coaching; 3.“Integration of Alternate Pain Management,” an evaluation of team-based primary care clinic readiness for physical therapy and substance use treatment integration.

**Findings:** Qualitative analysis of Primary Care 2.0 surfaced a disproportionate frequency of interview excerpts within the “Patient Needs and Resources” construct (Outer Setting domain), informing our published recommendations for a 6^th^ CFIR domain dedicated to patient perspectives. In subsequent analysis of Primary Care 2.0 “teamness”, we uncovered associations between care team functioning and wellness as they relate to implementation success. Precision Health pilot evaluation observations and interviews with front-line staff and patients highlighted the importance of health equity and cultural identity—factors not considered during initial pilot design but determined to impact pilot adoption, acceptability, and sustainability. Finally, applying CFIR as an observation framework to the Integration of Alternate Pain Management evaluation also surfaced team-based care themes across CFIR domains and highlighted that “teamness” influences organizational readiness for implementation.

**Implications for D&I Research:** CFIR V2’s attention to stakeholder needs across hierarchical levels and domains of implementation, whether as patients/end-users or frontline deliverers of an intervention, will add valuable depth and adaptation of interventions to better meet stakeholder assets and needs and ultimately improve implementation outcomes. We believe these new constructs will better capture patient priorities, teamness, and health equity and culture, considerations crucial for successful implementation.

**Primary Funding Source:** Department of Veterans Affairs

### S84 Incorporating a health equity focus in the consolidated framework for implementation research

#### Eva Woodward^1,2^

##### ^1^VA Center for Mental Healthcare and Outcomes Research, North Little Rock, AR, USA; ^2^University of Arkansas for Medical Sciences, Little Rock, AR, USA

###### **Correspondence:** Eva Woodward (Eva.woodward2@va.gov)

**Background:** The originally published version of the Consolidated Framework for Implementation Research (CFIR) , though comprehensive in its description of contextual determinants, did not include consideration of equity. CFIR V2, a recent update, does address equity but not to the extent addressed by the Health Equity Implementation Framework, that explicitly incorporates three health equity domains informed by literature. The aim of this presentation is to describe the Health Equity Implementation Framework, its application in a pilot study, and to highlight how this framework can help extend CFIR to address disparities.

**Methods:** Informed by literature searches, three health equity domains were determined to be key to understanding health care disparities and were not otherwise explicitly mentioned by CFIR. Using a consensus process among our research team, we compiled a list of sample qualitative and quantitative measures of each domain. We also piloted the three health equity domains from Health Equity Implementation Framework as an addition to CFIR domains in qualitative interviews. Interviews are being conducted via telephone with 33 providers and patients about perspectives of ongoing implementation of hospital policies to affirm lesbian, gay, bisexual, queer, and transgender (LGBTQ) Veterans in Veterans Health Administration.

**Findings:** The three health equity domains are: 1) societal context including social norms and stigmas and policies, economic influences, and physical structures in the built environment; 2) culturally relevant factors of recipients involved in implementation (e.g., end users, such as patients, and intermediaries, such as clinicians, staff); and 3) descriptors of the clinical encounter or moment when the adopters of the innovation interact with end users to offer the innovation. We present a definition, illustrative example, and sample quantitative and qualitative measures of each domain. We are finding the interview guide incorporating the three health equity and CFIR domains to be feasible and to capture more comprehensive data than using either set of domains alone.

**Implications for D&I Research:** Centering equity in implementation is urgently needed. In addition to using refined versions and tools for CFIR V2, researchers should link to the three domains from Health Equity Implementation Framework to better focus on issues and opportunities related to equity and disparities.

**Primary Funding Source:** Department of Veterans Affairs

### S85 Adaptation of Systems Engineering Methods to Support Practical Tailoring of Telehealth Implementation

#### Reid Parks^1^, Lelia Gessner^2,3^, Jane Evered^4^, Olayinka Shiyanbola^5^, Elizabeth Cox^2^, Edmond Ramly^3^

##### ^1^University of Wisconsin, Department of Industrial and Systems Engineering, Madison, WI, USA; ^2^University of Wisconsin School of Medicine and Public Health, Madison, WI, USA; ^3^University of Wisconsin-Madison College of Engineering, Madison, WI, USA; ^4^University of Wisconsin-Madison, Madison, WI, USA; ^5^University of Wisconsin-Madison, School of Pharmacy, Madison, WI, USA

###### **Correspondence:** Reid Parks (rparks5@wisc.edu)

**Background:** Tailoring implementation of interventions involves selecting strategies to address contextual determinants (barriers/facilitators) and improves implementation success. However, tailoring often yields too many determinants and strategies to be manageable in practice, creating a need for rational, systematic approaches to prioritization. Our objective was to demonstrate the need for prioritizing strategies and to adapt systems engineering methods into a process for prioritizing strategies by prioritizing determinants to support practical tailoring.

**Methods:** We conducted three 60-minute interviews with implementation facilitation professionals (n=5) about the practical relevance of our systematic process to prioritize determinants and support tailoring. We engaged stakeholder groups (n=12 patients, n=8 clinic staff) in separate 90-minute co-design sessions to identify determinants of telehealth implementation. We adapted two systems engineering methods to analyze outputs. First, Affinity Diagrams grouped stakeholder comments into conceptually-related determinants. Second, Interrelationship Digraphs examined relations among the determinants to define priority determinants as root causes (determinants influencing many other determinants) and key issues (determinants interrelated with many other determinants). Priority determinants were coded into the Consolidated Framework for Implementation Research (CFIR), which enabled selecting strategies from the Expert Recommendations for Implementing Change (ERIC) using the CFIR-ERIC Mapping Tool (Waltz et al. 2019).

**Findings:** Professional implementation facilitators professionals reinforced the need to systematically prioritize strategies to support practical tailoring. Representative quotes include, “My concern lies in the quantity. Is there a way to rank or limit strategies...as we know organizations have limits on their time...” and, “Essentially what is the critical path based on the challenges identified?” Compared to no prioritization, our process resulted in 72% fewer ERIC strategies to address CFIR determinants of telehealth implementation identified by stakeholders. Patient group comments represented 19 determinants mapping to 22 strategies. Prioritization led to 4 determinants mapping to 8 strategies. Clinic group comments represented 23 determinants mapping to 25 strategies. Prioritization led to 5 determinants mapping to 5 strategies

**Implications for D&I Research:** Prioritization is needed to support practical tailoring and our prioritization process using adapted systems engineering methods can be practically conducted using brief stakeholder sessions. This study addresses a critical gap by making tailored implementation practical and supports future work to develop and compare prioritization tools.

**Primary Funding Source:** NCATS CTSA Program

### S86 Implementation mapping to increase the use of evidence-based interventions in the HIV continuum of care

#### Katelin Hoskins^1,2^, Amanda Sanchez^1^, Carlin Hoffacker^1^, Florence Momplaisir^1,3^, Robert Gross^1,3^, Kathleen Brady^4^, Amy Pettit^5^, Kelly Zentgraf^1^, Chynna Mills^1^, Rinad Beidas^1,6,7^

##### ^1^University of Pennsylvania, Perelman School of Medicine, Philadelphia, PA, USA; ^2^University of Pennsylvania, Penn Implementation Science Center at the Leonard Davis Institute of Health Economics, Philadelphia, PA, USA; ^3^University of Pennsylvania, Center for Clinical Epidemiology and Biostatistics, Philadelphia, PA, USA; ^4^Philadelphia Department of Public Health, Philadelphia, PA, USA; ^5^Independent Consultant, Boston, MA, USA; ^6^University of Pennsylvania, Leonard Davis Institute of Health Economics, Philadelphia, PA, USA; ^7^University of Pennsylvania, Penn Medicine, Philadelphia, PA, USA

###### **Correspondence:** Katelin Hoskins (katelin.hoskins@pennmedicine.upenn.edu)

**Background:** Implementation mapping is a systematic, collaborative, and contextually attentive method for developing implementation strategies. As an exemplar, we apply this method to strategy development for Managed Problem Solving (MAPS), an evidence-based intervention for HIV medication adherence and viral suppression, which will be delivered by community health workers in an upcoming trial.

**Methods:** After conducting rapid analysis of 31 stakeholder interviews to identify determinants of MAPS implementation in clinics serving people living with HIV, our team held a first virtual stakeholder meeting to present preliminary findings and check our interpretations. We synthesized stakeholder feedback and mapped confirmed determinants to strategies listed in the ERIC taxonomy. Five strategies (e.g., warm handoffs) were identified directly from interview data. Strategies were cross-checked with the CFIR-ERIC Matching Tool to ensure that strategies with ≥25% endorsement were not overlooked. We operationalized each strategy with specific examples for clinic settings and linked them to relevant behavior change theories to provide a mechanistic understanding of their function. This content served as inputs into a logic model. We then held a second virtual stakeholder meeting to present our menu of implementation strategies and glean generalizable insights for (1) how these strategies might look in each stakeholder’s clinic and (2) which strategies were most feasible and impactful per stakeholders. We grouped strategies by their conceptual cluster to organize the menu presentation.

**Findings:** Implementation mapping produced a menu of 34 total strategies including revise professional roles, promote adaptability, leverage existing patient identification and referral processes, and change record systems. Identified strategies for outer setting determinants (e.g., stigma, technology disparities) were more limited. Stakeholder feedback was further synthesized to finalize a core menu of strategies to inform MAPS deployment. The process of implementation mapping generated key challenges and lessons for implementation strategy development: conceptual precision between facilitators and strategies; strategy identification for structural determinants; delineation of strategy form versus function; strategy temporality across phases of implementation; translation of implementation science constructs to stakeholders; and potential for prospective tracking of strategy use.

**Implications for D&I Research:** This work advances both MAPS implementation and implementation science methods by furthering our understanding of the use of implementation mapping to derive strategies.

**Primary Funding Source:** National Institutes of Health

### S87 Usability and initial findings of the longitudinal implementation strategy tracking system (LISTS) in the IMPACT consortium

#### Justin Smith^1^, Wynne Norton^2^, Whitney Batestilli^3^, Lila Rutten^4^, Sandra Mitchell^2^, Don Dizon^5^, Dr. Aaron Leppin^4^, Christine Cronin^6^, September Smith^7^, Jennifer Ridgeway^4^, Raymond Osarogiagbon^8^, Nadine McCleary^6^, Joan M. Griffin^4^, Joshua Richardson^9^, Frank Penedo^10^, Dr. Bryan Weiner^11^, Deborah Schrag^12^, Lisa DiMartino^3^

##### ^1^Room 1N410, University of Utah School of Medicine, Salt Lake City, UT, USA; ^2^National Cancer Institute, National Institutes of Health, Rockville, MD, USA; ^3^RTI International, Research Triangle Park, NC, USA; ^4^Mayo Clinic, Rochester, MN, USA; ^5^Brown University, Providence, RI, USA; ^6^Dana-Farber Cancer Institute, Boston, MA, USA; ^7^Northwestern University Feinberg School of Medicine, Chicago, IL, USA; ^8^Baptist Centers for Cancer Care, Memphis, TN, USA; ^9^Digital Health and Clinical Informatics, RTI International, Chicago, IL, USA; ^10^University of Miami, Coral Gables, FL, USA; ^11^University of Washington School of Public Health, Seattle, WA, USA; ^12^Dana-Farber/Harvard Cancer Center, Boston, MA, USA

###### **Correspondence:** Justin Smith (jd.smith@hsc.utah.edu)

**Background:** Understanding dynamic change of implementation strategies over time within and across healthcare intervention efforts is critical to implementation science. Yet, systematic approaches reporting strategies longitudinally are understudied, and existing approaches necessitate compromises between comprehensiveness and feasibility. We present findings regarding the usability and utility of the Longitudinal Implementation Strategy Tracking System (LISTS) within the National Cancer Institute-funded Improving the Management of Symptoms during and Following Cancer Treatment (IMPACT) Research Consortium, which includes three Research Centers (RCs) engaged in effectiveness-implementation trials testing routine symptom surveillance with evidence-based symptom management in ambulatory oncology care settings.

**Methods:** LISTS uses existing taxonomies and recommendations for specifying implementation strategies (e.g., actor, temporality, dose) and their modifications (FRAME-IS). Research team members and local implementers completed LISTS collaboratively at each RC using timeline-follow back procedures for 16 months and entered the results into a REDCap project created to align with the features of LISTS. Each RC completed a survey about LISTS use and each of the features, including the 10-item System Usability Scale (SUS) and open-ended questions.

**Findings:** 141 discrete strategies were used across the trials. Evaluative and iterative strategies accounted for 24%, followed by educate stakeholders (22%). Some trials more frequently used certain strategies (e.g., develop stakeholder interrelationships) in comparison to the other RCs. Although procedures for completing LISTS varied, those shared across RCs included using strategies from the Expert Recommendations for Implementing Change (ERIC) taxonomy as prompts; using calendars and meeting notes to verify LISTS elements; and having team/unit leaders review and sign off on strategies. Respondents rated the LISTS tool to be usable but with room for improvement (SUS Mean=67.5). “Dose” and “temporality” dimensions were rated as the most difficult to report. Open-ended feedback suggested familiarity with strategies, determinants, and implementation theory were critical to valid and reliable use.

**Implications for D&I Research:** LISTS advances implementation strategy measurement and tracking, characterizing dynamic features of change over time. Using a shared tracking approach within a consortium allows for comparison and synthesis across trials. Efforts are underway in other multi-site implementation studies to evaluate and refine LISTS to strengthen its measurement properties and interpretability.

**Primary Funding Source:** National Institutes of Health

### S88 Coaching to competency: Reducing implementation discontinuance and improving rates of success

#### Lisa Saldana, Zoe Alley, Jason Chapman, Holle Schaper

##### Oregon Social Learning Center, Eugene, OR, USA

###### **Correspondence:** Lisa Saldana (lisas@oslc.org)

**Background:**

Though many evidence-based practices (EBP) have associated purveyor-driven implementation strategies to improve practice uptake, the rate of EBP adoption remains dismal. Over the last decade, research with the Stages of Implementation Completion (SIC) has focused on operationalizing, tracking, and examining implementation process across a range of EBPs and strategies. Patterns of optimal implementation process have been identified by SIC Proportion (of implementation activities completed) and Duration (length of time to complete implementation activities) scores, and were used to inform development of a web-based performance system. SIC-Coaching then was designed to help purveyors guide newly adopting sites toward successful implementation by referencing the performance system. The impact of COVID-19 and its disruption on implementation process was considered.

**Methods:**

Three EBPs of varying levels of maturity, that utilize a purveyor-driven implementation process, were recruited. Each were ongoing users of the SIC to track implementation process and outcomes. Thus, historical SIC scores were available. Associated purveyors were recruited to participate including access to the performance system, coaching calls to guide interpretation, and use of the data collected from ongoing implementation.

**Findings:**

Fifty-six sites have been recruited across participating EBPs. Historical data were considered from EBP A (*n* = 369), B (*n* = 99) and C (*n* = 14). Compared to historical performance, SIC-Coaching sites showed a significant reduction in the odds that sites would discontinue implementation, but this reduction varied by EBP (A = 86% vs. 28%, *p* < .001; B = 46% vs 18%, *p* < .01; C = 71% vs 61% *p* = n.s.). Outcomes measured at time since implementation initiation (i.e., “age” of the implementation) suggest that despite COVID-19-imposed delays (measured by SIC outer context module), by 12-months post-implementation initiation, EBP A (*p* < .01), B (*p* < .001), and C (*p* < .01) all performed better than historical discontinuing sites, and similarly (*p* = n.s.) to historical sites that go on to succeed.

**Implications for D&I Research:**

The addition of SIC-Coaching to typical purveyor-driven implementation support has the potential to decrease the rates of discontinuing implementation efforts. Even in the midst of outer context challenges, use of SIC-Coaching holds promise for increased adoption of EBPs.

**Primary Funding Source:** National Institutes of Health

## Prevention and Public Health

### S89 Adapting a clinical exercise intervention for community delivery using the replicating effective programs framework

#### Taren Swindle^1,2^, Janna Martin^3^, Audrey Martinez^2^, Christi Arthur^4^, Daphne Gaulden^3^, Dong Zhang^4^, Elisabet Borsheim^2,4^, Aline Andres^2,3^

##### ^1^Univesity of Arkansas for Medical Sciences, Little Rock, AR, USA; ^2^Arkansas Children's Nutrition Center, Little Rock, AR, USA; ^3^University of Arkansas for Medical Sciences, Little Rock, AR, USA; ^4^University of Arkansas for Medical Sciences, Little Rock, USA

###### **Correspondence:** Taren Swindle (tswindle@uams.edu)

**Background:** Most pregnant women in the United States fail to achieve recommended physical activity (PA) levels. Innovative approaches to address barriers to PA during pregnancy are needed. The purpose of this study is to use the Replicating Effective Programs (REP) framework to adapt a clinic-based intervention for PA in pregnancy for community-based delivery. Reflecting stakeholder feedback, we report on the developmental phases of REP and the proposed implementation strategies.

**Methods:** The pre-conditions development phase of REP identified potential barriers through 10 interviews and 3 focus groups with participants who participated in the clinic-based intervention and had high, medium, and low compliance with the PA intervention. An Evidence-Based Quality Improvement (EBQI) Panel including 11 community stakeholders from diverse perspectives (e.g., Women, Infants and Children, faith-based organizations, perinatal mental health, Head Start, insurance) met on 5 occasions across 11 months to guide adaptations. The EBQI panel engaged in processes of concept mapping to generate and prioritize adaptations as well as collaborative discussion to review materials, advise on training, plan implementation strategies, and refine core intervention elements.

**Findings:** Interviews and focus groups identified 5 salient barriers: (1) time/schedule, (2) accessibility of location, (3) motivation/energy, (4) health concerns, and (5) care of other children. The EBQI panel prioritized 4 adaptions to address these barriers: (1) transition to virtual delivery format, (2) addition of trained mentor moms who were past participants, (3) addition of standardized educational content, (3) addition of post-partum support, and (4) shift in incentive structure (i.e., fewer monetary but more non-monetary incentives). Core elements maintained included the use of certified personal trainers as well as target PA duration, intensity, and frequency. Implementation strategies conceptualized included: (1) building community-academic partnerships to support implementation, (2) centralized technical assistance through a virtual platform, and (3) involving participant’s family members.

**Implications for D&I Research:** The adaptation to telehealth delivery reflects the needs of prior study participants and perspectives of key stakeholders for a sustainable intervention approach with potential to reach a broader audience for promotion of PA during pregnancy. REP provided a standardized, stakeholder-engaged model for guiding the process of adaptation of a clinical intervention for community delivery.

**Primary Funding Source:** United States Department of Agriculture

### S90 An evaluation of machine learning methods to improve the feasibility of fidelity monitoring of family-based prevention in primary care

#### Cady Berkel^1^, Dillon Knox^2^, Nikolaos Flemotomos^2^, David Atkins^3^, Shri Narayanan^2^

##### ^1^Arizona State University, Phoenix, AZ, USA; ^2^University of Southern California, Los Angeles, CA, USA; ^3^University of Washington, Seattle, WA, USA

###### **Correspondence:** Cady Berkel (cady.berkel@asu.edu)

**Background:** Evidence-based parenting programs effectively prevent the onset and escalation of child and adolescent behavioral and physical health problems. When programs have been taken to scale, declines in implementation fidelity diminish intervention effects. Gold standard methods of fidelity monitoring are cost-prohibitive and impractical in resource-scarce delivery systems. Technological developments using computational linguistics and machine learning offer an opportunity to assess fidelity in a low burden, timely, and comprehensive manner. In this study, we test automated machine learning methods to assess fidelity to the Family Check-Up 4 Health (FCU4Health) program. Implementation in the FCU4Health, is assessed via the COACH measure of competent adherence. FCU4Health is individually-tailored and uses Motivational Interviewing (MI) to engage parents in tailored support. As such, the COACH is highly focused on providers’ use of MI skills. Following the implementation cascade model, we examine how program providers’ delivery of the FCU4Health is associated with multiple indicators of parent engagement, which have been demonstrated to predict improvements in parenting and child outcomes.

**Methods:** This study makes use of data from a type 2 hybrid effectiveness-implementation trial of the FCU4Health conducted in partnership with primary care clinics. We analyzed transcripts from 113 families who participated in the program to develop machine ratings of fidelity, which we compared to human-rated COACH scores, multiple indicators of program engagement, and ultimately parenting and child outcomes. We trained and evaluated models using representations derived solely from the transcripts, including a bag-of-words representation and a multilingual pretrained embedding.

**Findings:** Computational models trained using only lexical information to predict COACH measures achieved a reliability similar to that of humans, and these modeling approaches trained to directly predict outcomes achieved performance significantly above baseline approaches. Specifically, using mean squared error, we were able to improve prediction of human ratings from a range of 0.83-1.02 to a range of 0.62-0.76, resulting in an approximate average improvement of 24%. Similarly, we were able to improve prediction of engagement indicators from a range of 0.81-27.3 to a range of 0.62-19.50, resulting in an approximate average improvement of 18%.

**Implications for D&I Research:** Computational models can be used to monitor fidelity to evidence-based interventions.

**Primary Funding Source:** Centers for Disease Control and Prevention

### S91 Making naloxone more convenient and anonymous to reach young adults

#### Nicole Wagner^1^, Dr. Allison Kempe^1^, Juliana Barnard^1^, Deborah Rinehart^2^, Edward Havranek^2^, Megan Morris^1^

##### ^1^University of Colorado Anschutz Medical Campus, Aurora, CO, USA; ^2^Denver Health, Denver, CO, USA

###### **Correspondence:** Nicole Wagner (nicole.wagner@cuanschutz.edu)

**Background:**

Naloxone, an opioid antagonist medication, can effectively reverse opioid overdose events with limited adverse events. The young adult population, at highest risk of opioid overdose, face barriers to naloxone access. Vending machine needle exchange programs in Europe, have reported increasing reach to young adults missed by other distribution strategies. Similar harm reduction vending machine (HRVM) programs have begun in the United States and have incorporated naloxone distribution. However, little is currently known about HRVM acceptability in young U.S. adults and what factors may contribute to reach. We conducted interviews with young adults to explore factors contributing to obtaining naloxone under current, ideal and HRVM distribution strategies.

**Methods:**

Our qualitatively trained researchers conducted interviews with 17 young adults receiving substance treatment services within an integrated urban safety net healthcare system. Participants were 18-30 years old and had witnessed or experienced an opioid overdose, or used nonmedical opioids in the last 4 months. This study used the practical, robust implementation and sustainability model (PRISM) to inform the interview guide, data collection and data analysis. Interviews were professionally transcribed and coded by team-based methods. Themes were developed using an inductive-deductive iterative approach and defined through consensus.

**Findings:**

Preliminary results (analysis completion anticipated for fall 2021) suggest barriers to naloxone under current distribution strategies for opioid users prior to treatment include: awareness of the strategy, cost, convenience, and anonymity. When describing ideal naloxone distribution implementation, participants prioritized convenience through multiple access points (including peers, parks, and concerts). Participants had overall positive feedback on HRVM, identifying cost, inclusion of sundries (birth control, socks), and external environment factors (location and law enforcement monitoring) contributing to perceived use. Female participants highlighted a need for potential safety measures.

**Implications for D&I Research:**

Young U.S. adults using substances are often difficult to reach using current health service strategies. Our preliminary results suggest providing anonymous and convenient access points to naloxone in the community will increase reach and uptake of naloxone, resulting in effective harm reduction. HRVM may present an opportunity to overcome barriers to reach. However, features of the physical location and cost should be considered in implementation decisions.

**Primary Funding Source:** National Institutes of Health

### S92 Adapting an evidence-based dating abuse prevention program for online delivery to promote engagement among teens and moms

#### Eliana Armora Langoni, Vangie A. Foshee, Natalie Blackburn, Kara Giannone, H. Luz Mcnaughton Reyes

##### University of North Carolina - Chapel Hill, Chapel Hill, NC, USA

###### **Correspondence:** Eliana Armora Langoni (elianaa@ad.unc.edu)

**Background:**

Research has found that adolescents who have been exposed to intimate partner violence (IPV) in the home are at higher risk for experiencing and perpetrating dating abuse (DA). Evidence-based interventions targeting youth to prevent DA exist, but are largely in-person or paper-based. Thus, we sought to describe the first phase of a feasibility trial in which we adapted Moms and Teens for Safe Dates (MTSD), an evidence-based six-booklet DA prevention program for moms who have experienced IPV to complete together with their teens, into an interactive and engaging online program.

**Methods:**

We followed the Iterative Decision-Making for Evaluation of Adaptations (IDEA) framework: ideation, prototype making, user testing, refining, and usability testing. Data on adaptation were collected from interviews with the research and web development teams (n=3), meeting minutes and reports, and feedback from target users in two sessions of a mother-teen advisory group (n=8), three rounds of user testing with mother-teen dyads (n=6), and one round of usability-testing (n=8).

**Findings:**

Phase one resulted in the development of a six-module online program for moms and teens. We converted 18 written stories from MTSD into animated and narrated still-image videos and 3 information sections into interactive audiovisual experiences. New content included one homepage video and six introductory animated videos at the beginning of each module. To ensure theory-based content remained in the adapted program, the research team identified the behavior change techniques used in the booklets and then website. User testing and research team experience identified the following considerations as central to adaptation for the online platform: balancing cost with impact, reducing literacy level and text burden while keeping content, establishing clear roles and responsibilities between web developers and researchers, and the need for a team with diverse skills to create interactive content.

**Implications for D&I Research:**

Adapting a “paper-based” program to an online format required numerous conversions of material into engaging formats and multiple rounds of testing and refining to ensure acceptability and functionality among end-users. Tracking theoretical constructs before and after adaptation ensured fidelity to the original evidence-based program. Other researchers and program developers can follow this process to adapt paper-based programs to online platforms.

**Primary Funding Source:** Centers for Disease Control and Prevention

### S93 The COVID-19 community-engaged risk communication (CERC) project: Using a soft systems approach to develop tailored implementation strategies in a rural black faith-based organization

#### Snigdha Peddireddy^1^, Monica Taylor^2^, Jada Gailliard^2,3^, Maya Wright^3^, McKayla Williams^2,4^, Leah Frerichs^3^, Christy Arnold^2^, Stephanie Battle^2^, James Gailliard^2^, Patsy Polston^3^, Lori Carter-Edwards^3^, Rohit Ramaswamy^3^

##### ^1^Emory University, Atlanta, GA, USA; ^2^Word Tabernacle Church, Rocky Mount, NC, USA; ^3^UNC Chapel Hill Gillings School of Global Public Health, Chapel Hill, NC, USA; ^4^UNC Chapel Hill, Chapel Hill, NC, USA

###### **Correspondence:** Snigdha Peddireddy (speddir@emory.edu)

**Background:**

The deeply inequitable experience of the COVID-19 pandemic between White and Black, Indigenous, and People of Color (BIPOC) communities underscores the need for culturally meaningful and context-appropriate public health risk communication messages. Social distancing constraints necessitated innovative implementation strategies to deliver these messages remotely. The COVID-19 Community-Engaged Risk Communication (CERC) Project is a partnership between an academic institution and Word Tabernacle Church (WTC), a Black faith-based organization. The collaboration used a soft systems participatory approach to explore social connections and access to technology in order to develop and deliver targeted messages about mitigating COVID-related insults.

**Methods:**

A purposive sample of congregants and church leaders was selected to construct “rich pictures” to visually represent key communication pathways among WTC leaders and congregants and communication-relevant emotional and experiential values. Rich pictures were assembled remotely, enriched through individual phone conversations and focus groups, and translated to adjacency matrices representing links between church leaders, congregants, and the broader community. Links were also described with attributes of information, means of communication, and emotional value. Matrix data were consolidated to create an overarching rich picture of connections across the entire WTC community, which was used to develop COVID-19 risk communication strategies.

**Findings:**

Systems mapping helped identify the most important communication flows among church leaders, congregants, ministries, and counseling and wellness groups. We also identified shared principles of communication, including giving and receiving love and prayer, investing in others for their elevation, and creating a platform where all can thrive, helping WTC establish deep-rooted connections important for creating trustworthy messages. These connections enabled a variety of viable modes for remote COVID-19-related messaging, including hand-written letters, e-newsletters, social media, and YouTube to accommodate the WTC congregants’ diverse needs.

**Implications for D&I Research:**

Faith-based communities are influential pro-social organizations whose social connections play a valuable role in engendering feelings of trust and belonging. Understanding how these connections function provides important contextual information for developing tailored implementation strategies for risk mitigation interventions. Participatory soft systems approaches that intentionally bring multiple stakeholders together to document their worldviews are powerful yet underutilized tools for learning and engagement in underserved community settings.

**Primary Funding Source:** UNC Chapel Hill Gillings Innovation Lab Award

### S94 Understanding the impact of COVID-19 on naloxone distribution from syringe service programs in the United States

#### Barrot Lambdin^1^, Lynn Wenger^2^, Ricky Bluthenthal^3^, Hansel Tookes^4^, Paul LaKosky^5^, Alex Kral^2^

##### ^1^RTI International, Berkeley, CA, USA; ^2^RTI International, San Francisco, CA, USA; ^3^University of Southern California, Los Angeles, CA, USA; ^4^University of Miami, Miami, FL, USA; ^5^North American Syringe Exchange Network, Tacoma, WA, USA

###### **Correspondence:** Barrot Lambdin (blambdin@rti.org)

**Background:** Syringe service programs (SSPs) have pioneered implementation of naloxone distribution. Naloxone is an opioid antagonist that reverses opioid overdoses, and studies have shown that higher levels of naloxone distribution reduce community-level opioid overdose mortality rates. SSPs are ideal venues for naloxone distribution, with staff who excel in providing culturally appropriate services for people who use drugs. We assessed the impact of the COVID-19 pandemic on naloxone distribution from SSPs and examined internal and external factors associated with higher levels of naloxone distribution during the pandemic.

**Methods:** We surveyed all known SSPs operating in the US in 2021. Out of the 431 SSPs, 325 (75%) responded to the online survey. We utilized mixed effects negative binomial regression to assess which factors were associated with the increasing naloxone distribution, adjusting for regional opioid overdose deaths from the prior year and number of annual SSP participants. We included SSP as a random effect.

**Findings:** SSPs distributed 662,954 naloxone doses in 2019 and 1,101,686 naloxone doses in 2020. The level of naloxone distribution from SSPs increased significantly in 2020 during COVID-19, compared to 2019 [adjusted incidence rate ratio (aIRR)=1.37; 95% CI: 1.21-1.56; p<0.001]. Compared to SSPs that were part of a local or state health department, SSPs that were a standalone non-profit organization, or part thereof, tended to have a significantly higher level of naloxone distribution [aIRR=3.47; 2.03-5.95; p<0.001], and SSPs with larger annual budgets tended to have significantly higher levels of naloxone distribution [aIRR= 1.72 per quartile; 95% CI: 1.37-2.14; p<0.001]. Regarding internal context, SSPs that had mobile delivery of naloxone (aIRR=1.47; 95% CI: 1.05-2.06; p=0.027) and secondary distribution of naloxone (aIRR=1.66; 1.13-2.42; p=0.009) tended to have higher levels of naloxone distribution.

**Implications for D&I Research:** We identified higher levels of SSP-based naloxone distribution during COVID-19. We also identified factors from the external and internal setting that were associated with higher levels of naloxone distribution. Ensuring non-profit SSPs are adequately resourced and that public health department SSPs have sufficient flexibility to adapt delivery models to meet the needs of the community are critical to optimize naloxone distribution and address the nation’s opioid overdose crisis.

**Primary Funding Source:** National Institutes of Health

### S95 Preparing to take the med-south lifestyle program to scale

#### Jennifer Leeman^1^, Carmen Samuel-Hodge^2^, Kiira Lyons^3^

##### ^1^School of Nursing, University of North Carolina, Chapel Hill, NC, USA; ^2^University of North Carolina, Chapel Hill, NC, USA; ^3^University of North Carolina at Chapel Hill, Chapel Hill, NC, USA

###### **Correspondence:** Jennifer Leeman (jleeman@email.unc.edu)

**Background:** Barker’s scale-up framework guided our four-phase process for scaling-up the Med-South Lifestyle Program (Med-South), an evidence-based lifestyle counseling intervention that translates the Mediterranean dietary pattern for the southeastern U.S. In Phase 1 (set-up), we created an advisory board with stakeholders from underserved communities and regional and state-level organizations and decided to scale-up in federally qualified health centers (FQHCs) and health departments (HDs). In Phase 2 (develop the scalable unit), we partnered with our Advisory Board and four FQHCs/HDs to develop and pilot test the *Med-South change package* (e.g., intervention protocols, participant handbook, workflows for identifying and referring participants, etc.) and began to develop *scale-up strategies* (2014-2019). In Phase 3 (test scale-up), we piloted scale-up strategies in four sites (2020-2021) and in Phase 4 (going to scale) will take Med-South to scale across 20 sites (2021-2023). We report methods and findings from the Phase 3 pilot.

**Methods:** We used a pre-post, hybrid type 3 implementation-effectiveness design. Each site (n=4) identified staff to deliver and implement Med-South. Scale-up strategies included (1) education materials, (2) training, and (3) monthly facilitation calls. Due to COVID-19, we transitioned to virtual delivery of scale-up strategies and the intervention. Quantitative measures (surveys, tracking logs, and biologic) included counselor self-efficacy to deliver Med-South, implementation outcomes (engagement, reach, acceptability, feasibility, fidelity, and cost), and effectiveness outcomes (weight, blood pressure, physical activity, dietary intake). Qualitative measures (interviews) assessed implementation determinants and local adaptations. Quantitative data will be analyzed using summary statistics. The CFIR and FRAME will guide content analysis of determinants and adaptations.

**Findings:** Staff engagement in scale-up strategies was high. Reach to participants was lower than planned due to COVID-19; 41 participants enrolled (54% AA, 81% women), 20 of whom have completed all 7 sessions, 11 are in process, and 3 dropped out. Data collection and analysis will be completed in August 2021.

**Implications for D&I Research:** Findings from the pilot will be used to further refine scale-up strategies, which we will then test across 20 sites. A multiphase approach is key to strategically engaging multilevel stakeholders to design both a change package and the strategies needed to take an intervention to scale statewide.

**Primary Funding Source:** Centers for Disease Control and Prevention

### S96 Involving pharmacy stakeholders to identify barriers and facilitators of opioid use disorder prevention interventions

#### Deepika Rao^1^, Megan Mercier^2^, Christine Mcatee^3^, James H Ford II^1^, Olayinka Shiyanbola^4^

##### ^1^School of Pharmacy, University of Wisconsin-Madison, Madison, WI, USA; ^2^Univerity of Wisconsin-Madison, Madison, WI, USA; ^3^Madison, WI, USA; ^4^University of Wisconsin-Madison, School of Pharmacy, Madison, WI, USA

###### **Correspondence:** Deepika Rao (dmrao@wisc.edu)

**Background:**

Opioid use disorder (OUD) is a public health crisis, exacerbated by lack of access to prevention and treatment. Community pharmacists who routinely interact with patients are easily accessible to provide OUD services. Pharmacists can aid in prevention of OUD by contributing to pain management plans, counseling patients, screening for opioid misuse, and dispensing naloxone. However, interventions designed to increase the role of the pharmacist in OUD prevention have not been translated into practice. Dissemination and implementation research focusing on evidence-based interventions (EBI) for OUD in community pharmacies is lacking. Pharmacist needs and barriers must be addressed to implement these EBI effectively. The study objective was to qualitatively explore barriers and facilitators regarding OUD prevention among community pharmacists.

**Methods:**

Using the Consolidated Framework for Implementation Research (CFIR), we conducted 11 semi-structured 60-min interviews with community pharmacists. A purposeful sample of English-speaking pharmacists practicing in different types of pharmacies (small independent, large-chain, specialty retail) and in varied positions (managers, owners, full-time/part-time pharmacists) was used. The interview guide was piloted in 2 interviews and probing questions were added. Transcriptions were analyzed using deductive content analysis based on CFIR domains, followed by an inductive in-vivo coding to identify themes.

**Findings:**

Themes around the CFIR domains of Individual Characteristics, Inner Setting, and Outer Setting were identified. Pharmacists communicated a lack of knowledge regarding screening and harm reduction services, described stigmatizing attitudes towards patients, and did not a perceive a strong need for prevention, which are potential barriers to implementation at the individual level. Individual and structural level facilitators included importance of educating patients about OUD; a motivation to improve patient outcomes and relationships; and a belief that OUD prevention is compatible with their organization goals and pharmacy structure.

**Implications for D&I Research:**

Effective translation of EBI for OUD begins with addressing barriers and facilitators. We identified barriers to implementation, which could be addressed by pharmacy-based strategies focused on increasing tailored knowledge among pharmacists, reducing bias, and emphasizing a need for change. Successful translation of EBI also requires emphasizing facilitators found at the individual and structural levels, such as pharmacist motivation and using existing work processes, such as tele-pharmacy, to adapt interventions.

**Primary Funding Source:** University Award

### S97 Common challenges among established mobile market organizations and implications for implementation of evidence based interventions

#### Christina Kasprzak^1^, Anne Lally^1^, Julia Schoonover^2^, Deanna Gallicchio^3^, Lindsey Haynes-Maslow^4^, Leah Vermont^1^, Alice Ammerman^5^, Samina Raja^6^, Laurene Tumiel-Berhalter^1^, Jill Tirabissi^3^, Lucia Leone^2^

##### ^1^University at Buffalo School of Public Health, BUFFALO, NY, USA; ^2^University at Buffalo, Buffalo, NY, USA; ^3^University at Buffalo, Buffalo, USA; ^4^Raleigh, USA; ^5^The University of North Carolina at Chapel Hill, Chapel Hill, NC, USA; ^6^University at Buffalo School of Architecture and Planning, Buffalo, NY, USA

###### **Correspondence:** Christina Kasprzak (cmk27@buffalo.edu)

**Background:**

Mobile produce markets are becoming an increasingly prevalent, accepted, and effective strategy for increasing fruit and vegetable access and consumption in lower-income and underserved communities. However, there is limited published research on mobile market operations. This research aims to identify the challenges mobile markets face and develop ways to potentially mitigate those challenges to improve implementation of the Veggie Van program, an evidence-based mobile market model.

**Methods:**

We conducted 21 semi-structured key informant (KI) interviews to assess common practices of mobile market organizations operating for 2+ years. We asked KIs about their organizational structure, operations, procurement and logistics, evaluation efforts, marketing and community engagement, success, and challenges. All interviews were recorded, transcribed, and analyzed by two independent coders using ATLAS.ti 8.0 qualitative software. A secondary analysis of code reports and memos identified subthemes related exclusively to common challenges and remedial practices. An inductive coding process was applied to match identified challenges to the appropriate Consolidated Framework for Implementation Research (CFIR) domains.

**Findings:**

The leading challenges cited by KIs correspond to the CFIR domains of inner setting (e.g., funding and resources), outer setting (e.g., navigating regulations), and process (e.g. engaging community partnership). Highlighted practices employed by organizations may mitigate persistent challenges. For example, maximizing ancillary services may enhance financial sustainability of the mobile market. Adopting innovative staffing structures such as a community advocate or champion model may be more cost effective and ensure that staff are representative of the communities visited. Formalizing agreements and expectations with community partners may strengthen relationships with host sites and increase the viability of market sites.

**Implications for D&I Research:**

This research demonstrates the utility of CFIR to uncover contextual factors that may impact implementation of evidence-based interventions in community organizations. Specifically, our team will be adapting the Veggie Van model to address these challenges prior to further implementation and dissemination, but the recommended practices could be applied across various community settings.

**Primary Funding Source:** National Institutes of Health

### S98 Assessing the implementation of the national diabetes prevention program using the consolidated framework for implementation research

#### Lillian Madrigal^1^, Olivia Manders^1^, Mary Beth Weber^1^, Linelle Blais^1^, Regine Haardörfer^2^, Michelle Kegler^1^

##### ^1^Emory University, Atlanta, GA, USA; ^2^Rollins School of Public Health, Emory University, Atlanta, GA, USA

###### **Correspondence:** Lillian Madrigal (lmadrig@emory.edu)

**Background:** The CDC’s National Diabetes Prevention Program (DPP) has made great strides in raising awareness for and accessibility to its evidence-based lifestyle change program. Currently over 1,500 organizations nationwide deliver the program. To date, no systematic and rigorous study of the organizational- and structural-level causal factors related to National DPP implementation outcomes has been conducted.

**Methods:** The Consolidated Framework for Implementation Research (CFIR) is a meta-theory comprised of constructs that have been associated with effective implementation. As part of a mixed methods study to evaluate the National DPP implementation, we applied CFIR’s inner and outer setting constructs to describe organizational- and structural-level influences on the program. We used a qualitative cross-case construct rating methodology to assess which CFIR constructs contributed (both in magnitude and valence) to the organization’s current level of implementation reach (measured by cumulative participant enrollment).

Eligible organizations were stratified into high, medium, and low implementation levels based on enrollment numbers at the time of the interview, then purposively selected to sample the diversity of implementers including healthcare systems, clinics, community-based organizations, government agencies and academic institutions by length of program delivery, urbanicity, populations served, and size. Thirty National DPP organization key informants located in 24 states and territories were interviewed between August 2020 and January 2021.

Transcripts were double-coded and intercoder agreement was performed on one third of the transcripts to ensure coder reliability. Two coders assessed all transcripts by construct for valence (positive or negative influence on implementation) using construct rating criteria and scored with a 5-point bi-polar scale. An equal mix of positive and negative influences received a score of zero.

**Findings:** The preliminary analysis found several distinguishing CFIR inner and outer setting constructs by level of implementation. Higher implementation organizations more often discussed positive implementation influences within the “culture”, “compatibility”, “available resources”, and “cosmopolitanism” constructs. Low implementation organizations described more negative influences across all constructs, particularly within “structural characteristics” and “patient needs & resources”. Full analysis will be completed this fall.

**Implications for D&I Research:** This research aims to exemplify how CFIR can be used to identify the complex causal factors to improve dissemination and implementation of evidence-based prevention programs.

**Primary Funding Source:** Centers for Disease Control and Prevention

### S99 Evaluation of a large-scale school wellness intervention through the consolidated framework for implementation research (CFIR): Implications for dissemination and sustainability

#### Gabriella McLoughlin^1,2^, Rachel Sweeney^3^, Laura Liechty^3^, Joey Lee^4^, Richard Rosenkranz^5^, Gregory Welk^6^

##### ^1^Washington University in St. Louis, St. Louis, MO, USA; ^2^Temple University, Philadelphia, PA, USA; ^3^Iowa State University, Ames, IA, USA; ^4^University of Colorado Colorado Springs, Colorado Springs, CO, USA; ^5^Kansas State University, Manhattan, KS, USA; ^6^Iowa State University, Ames, USA

###### **Correspondence:** Gabriella McLoughlin (gabriella.mcloughlin@temple.edu)

**Background:** The need for sustainable and scalable comprehensive school wellness interventions is evident, and the lack of attention toward capacity-building models warrants investigation. Furthermore, there is a dearth of understanding regarding implementation determinants grounded in dissemination and implementation (D&I) frameworks. This study sought to address: 1) implementation determinants of adoption, fidelity, and penetration for school-wide wellness programming; and 2) nuanced determinants between schools with prior experience and those new to the program, to enhance tailored implementation support and sustainability.

**Methods:** The School Wellness Integration Targeting Child Health (SWITCH®) capacity-building intervention was adopted in 52 elementary and middle (22 new; 30 experienced) schools across Iowa, United States in the 2019–2020 academic year. Mixed methods data collection and analysis procedures followed the Consolidated Framework for Implementation Research (CFIR) protocols, adapted to school settings. Implementation outcomes included: 1) fidelity/compliance to established quality elements; 2) adoption of best practices in multiple settings; and 3) penetration of behavior change practices across classrooms and grade levels. Assessed determinants comprised organizational readiness/capacity and CFIR constructs via interviews and surveys. Interview data were scored using a systematic process; each CFIR domain was assigned a score (ranging between -2 and +2) to denote either a positive or negative influence on implementation. Independent t-tests were conducted to capture potential differences between new and experienced schools, followed by Pearson bivariate correlation analyses to determine relationships between CFIR determinants and implementation outcomes.

**Findings:** Experienced schools reported insignificantly higher fidelity ( t =-1.86 p= .07) and higher rates of adoption ( t =-2.03 p= .04) compared to new schools. Correlation analyses revealed positive relationships between implementation outcomes and CFIR determinants including innovation source, culture and relative priority, and leadership engagement. Negative relationships were observed in tension for change and networks and communications. Specific negative relationships for new schools between determinants and outcomes included relative advantage, engaging key stakeholders, and reflecting/evaluating, among others.

**Implications for D&I Research:** Findings highlight the specific relationships between implementation outcomes and determinants; nuanced challenges for new schools highlight the need for a more tailored approach to implementation support and offer insights for sustainability. Adapted CFIR protocols provide opportunities for replication in other school-and community-based projects.

**Primary Funding Source:** United States Department of Agriculture

## Promoting Health Equity and Eliminating Disparities

### S100 Equitable implementation to eliminate cardiovascular health disparities on Chicago’s south side: A description of implementation preparation activities

#### J.D. Smith^1^, Paris Davis^2^, Abel Kho^3^, Allison Carroll^4^, Simon Gordan^5^

##### ^1^University of Utah School of Medicine, Salt Lake City, UT, USA; ^2^Triedstone Full Gospel Baptist Church, Chicago, IL, USA; ^3^Northwestern University, Feinberg School of Medicine, Chicago, IL, USA; ^4^Northwestern University Feinberg School of Medicine, Chicago, IL, USA; ^5^Triedstone Church of Chicago, Chicago, IL, USA

###### **Correspondence:** J.D. Smith (jd.smith@hsc.utah.edu)

**Background:**

Health inequities in the US healthcare system are due to disparities in access, insurance coverage, disinvestment in communities of color, and are rooted in systemic racism and medical mistrust. Implementation science has begun to explicitly focus on ways to eliminate health disparities through “equitable implementation” approaches. Central to understanding how to achieve equitable implementation are community-driven solutions built on trusted voices from within the community. This inclusion in the study and practice of implementing health care innovations for health disparity populations will set a precedence for forming the foundation needed to address common goals among stakeholders. We describe a community-driven approach for equitable implementation strategies being used in a two-stage project aimed at eliminating cardiovascular health disparities among African Americans on Chicago’s South Side.

**Methods:**

First, participatory approaches were used with community stakeholders to select an evidence-based blood pressure control intervention, and through a pilot study that leverages both faith-based organizations (FBOs)—a trusted source of health information for African Americans—and federally-qualified health centers (FQHCs) in South Side neighborhoods, the intervention is being adapted using the Dynamic Adaptation Process. Second, the adapted intervention, and the coordinated implementation strategy between FBOs and FQHCs resulting from the pilot, will be tested using a Type III hybrid effectiveness-implementation trial. The primary outcome—Public Health Impact (reach rate * blood pressure control effect size) will be computed for comparison with usual care in West Side Chicago as a non-randomized comparison group.

**Findings:**

We identified a need for consistency in language and expectations at the onset of the project among all community participants and stakeholders to foster steady forward progress toward fulfilling the project's goals. Doing so resulted in selecting an intervention (Kaiser bundle) and creating trust between stakeholders.

**Implications for D&I Research:**

Community-driven approaches to implementation of interventions for conditions with widespread health disparities is desperately needed if equity is to be achieved. Applying the concept of equitable implementation requires a shift in current implementation science practices toward tailoring strategies to communities in ways that recognize the role of racism and other societal and healthcare system factors.

**Primary Funding Source:** National Institutes of Health

### S101 Using community defined evidence to guide equitable implementation for underserved communities

#### Linda Callejas^1^, Gilberto Perez^2^, Francisco Limon^3^

##### ^1^University of South Florida, Tampa, FL, USA; ^2^Bienvenido Community Solutions, Goshen, IN, USA; ^3^Bienvenido Community Solutions, Winterville, NC, USA

###### **Correspondence:** Linda Callejas (callejas@usf.edu)

**Background:** Despite the continued influence of the evidence-based practice movement in behavioral health over the last several decades, the use of EBPs has not reduced disparities in Latinx and other racialized communities. In response, researchers and practitioners have called for increased ‘community involvement’ in effectiveness studies and program implementation efforts to improve service delivery and promote widespread community well-being. Community involvement in the implementation of behavioral health interventions often focuses on applying strategies to identify a population’s behavioral health needs, on engaging hard-to-reach communities in the use of interventions developed and tested in clinical settings, and, in some cases, on soliciting input to modify existing interventions and make them more relevant to members of these communities. Yet, these strategies are researcher-initiated and rely on community members as informants rather than experts in their own right.

**Methods:**

We present community-defined evidence (CDE) as a potential framework for equitable implementation. CDE depends on active collaboration of local residents in the development and use of culturally responsive, community-focused interventions that address their social and behavioral conditions, as these residents define them. As part of our discussion on CDE we will discuss the Bienvenido Program, a mental health promotion program co-developed in Ligonier, Indiana by Latinx practitioners and community members.

**Findings:**

Participants reported increased use of strategies for coping with stressors and improved access to mental health services. Participants also reported increased confidence about participating in community meetings, interacting with local elected officials, and speaking to health professionals about their health and potential treatments. Bienvenido established a partnership with the National Network to Eliminate Disparities in Behavioral Health to train mental health professionals and leaders in 20 community-based organizations around the country.

**Implications for D&I Research:** The current movement toward equitable implementation requires centering community perspectives in implementation research and practice. This example provides strategies for developing and testing community-defined evidence.

**Primary Funding Source:** SAMHSA

### S102 Equitable implementation at work

#### Allison Metz^1^, Beadsie Woo^2^, Audrey Loper^3^

##### ^1^UNC School of Social Work, Chapel Hill, NC, USA; ^2^Annie E Casey Foundation, Baltimore, MD, USA;^3^Frank Porter Graham Child Development Institute, Carrboro, NC, USA

###### **Correspondence:** Allison Metz (allison.metz@unc.edu)

**Background:**

Implementation frameworks, theories, and models have not explicitly focused on how implementation can advance equity. We propose a new lens of *equitable* implementation: an explicit and intentional integration of implementation science and equity that attends to what is being delivered, for whom, and under what conditions; and how delivery should be tailored to best meet the needs of the focus population. Equitable implementation occurs when strong equity components—including explicit attention to the culture, history, values, assets, and needs of the community—are integrated into the principles, strategies, frameworks, and tools of implementation science.

**Methods:** Ten recommendations emerged through thematic analysis of diverse case examples in the Stanford Social Innovation Review supplement on equitable implementation. Recommendations were vetted with authors.

**Findings:**

1. Take the time to build trust through small, frequent interactions.

2. Shed the solo leader model of implementation and support community members tp develop a shared understanding of problems and potential solutions.

3. Distribute decision-making authority and information to those whose lives are most affected by the implementation.

4. Engage in deliberate and transparent decision-making.

5. Engage community members in interpreting and using data to support implementation.

6. Develop community-defined evidence by co-designing interventions with community members.

7. Seek locally based service delivery platforms which will increase access for and uptake by local residents.

8. Address issues of social justice through program adaptations that address barriers to care that are rooted in systemic and structural racism.

9. Develop implementation strategies that impact the contextual factors that contribute to disparities in outcomes such advocacy and policy implementation strategies focused on the macro context.

10. Seek long-term outcomes that advance equity by selecting programs that have the potential of a spillover effect in outcomes is a mechanism for equitable implementation.

**Implications for D&I Research:** Recommendations inform the development of research questions, the methods used, and how implementation scientists approach work with communities.

**Primary Funding Source:** Annie E Casey Foundation

### S103 Developing methods for equity guided implementation and adaptation in cancer control interventions

#### Kelly Aschbrenner^1^, Gina Kruse^2^, Karen Emmons^3^, Dr. Stephen Bartels^4^

##### ^1^Geisel School of Medicine at Dartmouth College, Nashua, NH, USA; ^2^Massachusetts General Hospital, Boston, MA, USA; ^3^Harvard University, Boston, MA, USA; ^4^Massachusetts General Hospital, Harvard Medical School, Boston, MA, USA

###### **Correspondence:** Kelly Aschbrenner (kelly.aschbrenner@dartmouth.edu)

**Background:** To advance equitable implementation of evidence-based interventions (EBIs), researchers and healthcare partners need tools and strategies to identify and prioritize health equity targets and plan, adapt and monitor EBI implementation. In our Harvard Implementation Science Center for Cancer Control Equity (ISCCCE), we are engaging healthcare partners in developing methods at the intersection of quality improvement and implementation science to promote health equity. Our methods pilot study developing and evaluating an equity-guided adaptation process (EGAP), a collaborative process of using data and stakeholder input to guide implementation of cancer control interventions, illustrates this work.

**Methods:** We are evaluating the feasibility and acceptability of EGAP informed by the Dynamic Adaptation Process in a pilot hybrid implementation-effectiveness study of paired promotion of colorectal cancer and social needs screening at four Federally Qualified Community Health Centers (FQHCs). Using a mixed methods convergent design, we are evaluating the feasibility and acceptability of EGAP and engaging FQHC leadership and staff in model development. We have identified adaptations and other strategies used to promote health equity across dimensions prioritized by the FQHCs (e.g., race/ethnicity, gender, disability.).

**Findings:** FQHC implementation team size has ranged from 4 to 7 members and each team has included quality improvement or population health personnel. With external facilitation, sites have used clinic data to identify patient groups experiencing inequities in reach and effectiveness of the dual screening intervention. For example, language accessibility gaps have been common across sites. Adaptations to address these gaps have included modifying pre-visit planning to identify and pair patients with limited English proficiency with interpreter services and translation and cultural adaptation of patient materials from English to other languages.

**Implications for D&I Research:** Partnering with healthcare stakeholders to develop methods that combine data with practice experience to prioritize health equity targets, adapt, and monitor EBI implementation and evaluate health equity outcomes will advance the D&I goal of rigorous and relevant methods for improving health equity.

**Primary Funding Source:** National Institutes of Health

### S104 Targeting rural health disparities in lung cancer screening by co-creating a decision aid

#### Amy Huebschmann^1^, Jodi Holtrop^2^, Dan Matlock^3^, Russell Glasgow^1^

##### ^1^University of Colorado School of Medicine, Aurora, CO, USA; ^2^University of Colorado Anschutz Medical Campus, Denver, USA; ^3^University of Colorado, Aurora, CO, USA

###### **Correspondence:** Amy Huebschmann (amy.huebschmann@cuanschutz.edu)

**Background:** In our Colorado Implementation Science Center for Cancer Control and Prevention (Colorado ISC3), we developed innovative methods to improve inequities in rural cancer prevention and control. Guided by our expanded Reach, Effectiveness, Adoption, Implementation and Maintenance (RE-AIM) framework, we explicitly consider factors related to equity and representativeness among multi-level stakeholders in rural primary care. We operationalized these methods to target disparately low rates of the evidence-based practice of lung cancer screening (LCS) in rural areas, by co-creating a decision aid to facilitate the shared-decision making (SDM) mandate of insurers.

**Methods:** According to our expanded RE-AIM framework, we qualitatively assessed the rural cultural and contextual factors related to lung cancer screening (LCS) delivery, with particular attention to perspectives among rural patients and primary care clinicians/staff, as well as the organizational capacity of clinics, and external environment policy factors. Interviews were conducted with rural patients, primary care leaders, clinicians, staff, and community members. In a human-centered design process, we then iteratively developed a novel LCS decision aid with these stakeholders, in order to address multi-level barriers to LCS implementation.

**Findings:** We identified key barriers to LCS implementation that a decision aid could address, including the need to build clinician’s self-efficacy to provide SDM for LCS through specific prompts (e.g., assessing patients’ values), and to overcome some patients’ resistance to LCS counseling due to a lack of perceived benefit. Rural clinicians did not currently use decision aids to support LCS counseling, and found current decision aids infeasibly long to use. In our iterative process of developing an acceptable LCS decision aid for our stakeholders, we addressed preferences for brevity and clear representation of risks and benefits, using visuals and simple language appropriate for low-literacy populations.

**Implications for D&I Research:** Our resultant brief, pragmatic decision aid to guide SDM for LCS counseling has great potential to increase equitable adoption of guideline implementation and equitable reach in rural areas. Using the expanded RE-AIM framework to conceptualize barriers at multiple socio-ecological levels and to co-create solutions using human-centered design methods offer opportunities to overcome inequities by meeting the cultural needs and priorities of stakeholders.

**Primary Funding Source:** National Institutes of Health

### S105 Adoption of telemedicine and adaptation of cancer care delivery in community health centers during the COVID-19 pandemic

#### Nathalie Huguet, Susan Flocke, Heather Angier, Jennifer DeVoe, Leah Gordon, Tahlia Hodes, Andrea Baron, Maria Danna, Heather Holderness, Deborah Cohen

##### Oregon Health & Science University, Portland, OR, USA

###### **Correspondence:** Nathalie Huguet (huguetn@Ohsu.edu)

**Background:** Guided by the Practice Change Model and Strategic Implementation Framework, the BRIDGE-C2 Center focuses on identifying the factors influencing adoption of and approaches to tailor implementation strategies and the level of implementation support needed. With COVID-19, community health centers (CHCs) rapidly changed the way they delivered healthcare. Learnings from how practices have adapted to ensure equitable delivery of cancer preventive care, the tools they implemented, and how they overcame barriers will greatly inform future implementation science efforts.

**Methods:** This mixed methods study used electronic health record data from 224 CHCs and data from 26 interviews from a sample of 8 CHCs with high cancer screening performance pre-COVID-19. Practices were purposively sampled for variation on geographic region, rurality, and patient demographics. Data were from January 2020 (pre-COVID-19) to October 2020. Outcome measures included change in telemedicine visit rates and cervical, colorectal cancer screening rates, and barriers/facilitators influencing adoption and implementation of telemedicine and changes to cancer screening.

**Findings:** Across the CHCs, telemedicine visit rates increased by 1237% at the onset of the pandemic in March 2020, while cancer screening rates (cervical=-61%; colorectal=-58%) showed large declines. By October, rates at urban CHCs had returned to pre-COVID-19 levels, while rural CHCs rates were at half their pre-COVID-19 levels. CHC interviewees reported challenges to care delivery related to technology (e.g., insufficient broadband especially in rural areas), local hospital closures (e.g., limited colonoscopy access), inadequate staff capacity, and patient reluctance for in-person care. Clinics reported overcoming challenges to rapid care delivery adaptation with leadership support, quality improvement experiences that guided adoption of telemedicine (e.g., using PDSA processes), and patient outreach. CHCs also developed ways to shift cancer screening care away from the office (e.g., mailed fecal tests) to reach more patients.

**Implications for D&I Research:** COVID-19 forced CHCs to adopt and adapt new ways of providing routine and cancer screening care. Rural sites and their patients experienced greater challenges to change. These findings highlight the importance of the practices’ outer context and of developing tailored approaches to ensure successful implementation and equitable care delivery.

**Primary Funding Source:** National Institutes of Health

### S106 Rapid development and implementation of a sars-cov-2 testing community-based intervention

#### Lara Savas^1^, Meghan Haffey^2^, Christine Markham^2^, Belinda Reininger^3^, Paula Cuccaro^4^, Maribel Sifuentes^3^, Emily Adlparvar^2^, Carlton Allen^5^, Rodrigo Hernandez^2^, Jessica Molina^2^, Sarah Montgomery^2^, Paola Vidal^3^, Margaret Goetz^6^, Maria Fernandez^4^

##### ^1^University of Texas Health Science Center at Houston (UTHealth), Houston, TX, USA; ^2^University of Texas Health Science Center at Houston, Houston, TX, USA; ^3^University of Texas Health Science Center at Brownsville, Brownsville, TX, USA; ^4^The University of Texas Health Science Center at Houston School of Public Health, Houston, TX, USA; ^5^University of Texas Health Science Center at Tyler, Tyler, TX, USA; ^6^ProSalud, Houston, TX, USA

###### **Correspondence:** Lara Savas (lara.staub@uth.tmc.edu)

**Background:** Rapid, evidence-based behavioral intervention response to increase SARS-CoV-2 testing during the COVID-19 pandemic has been essential. We used Intervention Mapping (IM) as the foundational framework to guide rapid development for implementing a COVID-19 testing motivation and access intervention targeting underserved populations in Texas.

**Methods:** We used the IM framework and a community-engaged approach to guide rapid development and implementation of a SARS-CoV-2 testing intervention. The six-step systematic planning process included conducting a needs assessment, selecting determinants based on SARS-CoV-2 testing behaviors, barriers and facilitators, and identifying evidence-based strategies (e.g. use of role modeling of accessing testing and testimonials for testing success stories).To accelerate development of implementation strategies, we simultaneously developed program design components based on knowledge and evidence from previous community-based behavioral health interventions (e.g., use of community health workers, CHWs, social marketing strategies, and health coaching to address psychosocial and healthcare access barriers); thus using a “right to left and left to right” approach to development of strategies. That is, we used systematic planning and rapid development, with validation and tailoring informed by the planning and community engagement efforts. Our use of IM and community engagement facilitated a process for rapid adaptation of materials and implementation planning strategies to reflect changes in the environment and community preferences, an approach we refer to as the “Community Just in Time Adaptive Intervention – Community JITIAI.”

**Findings:** This simultaneous process supported rapid development of theoretical and evidence-based multi-level implementation strategies. The IM methodology provided a systematic framework to plan the methods and strategies that were embedded in the concurrent development of program components, including CHW-delivered education and motivation, social marketing materials, and one-one-one telephone-based education. This “meeting-halfway on the six steps” resulted in rapid development and implementation of a theoretically informed and evidence-based multicomponent program.

**Implications for D&I Research:** To ensure program relevance, program planners continue to use this approach to rapidly adapt program content to the changing testing behaviors and demands of the pandemic. The COVID-19 pandemic compelled researchers to expedite the program development approach. This study provides a model for using IM for rapid program development.

**Primary Funding Source:** National Institutes of Health

### S107 A community-engaged approach to inform adaptations to a technology-based intervention to support pre-exposure prophylaxis (PrEP) adherence among sexual and gender minorities

#### Jessica Montoya^1,2,3^, Vanessa Serrano^1,4^, Arin Fisher^1^, Alvy Rangel^5^, Phoebe Lyman^5^, Risa Flynn^5^, Cathleen Willging^6^, Maile Karris^1,2^, Gregory Aarons^3,7^, David Moore^1,2^

##### ^1^UC San Diego, San Diego, CA, USA; ^2^UC San Diego Center for AIDS Research, La Jolla, CA, USA; ^3^UC San Diego Altman Clinical and Translational Research Institute Dissemination and Implementation Science Center, La Jolla, CA, USA; ^4^San Diego State University/UC San Diego Joint Doctoral Program in Clinical Psychology, San Diego, CA, USA; ^5^Los Angeles LGBT Center, Los Angeles, CA, USA; ^6^Behavioral Health Research Center of the Southwest, Pacific Institute for Research and Evaluation, Albuquerque, NM, USA; ^7^UC San Diego, La Jolla, CA, USA

###### **Correspondence:** Jessica Montoya (jlmontoy@health.ucsd.edu)

**Background:**

Preventing new HIV transmissions by using pre-exposure prophylaxis (PrEP) is a key pillar in the U.S. Ending the HIV Epidemic (EHE) initiative. Adherence to PrEP is necessary to confer adequate protection from HIV. The individualized Texting for Adherence Building (iTAB) system is recognized by the CDC as an evidence-based intervention for PrEP adherence. iTAB has several key components, including two-way communication, individually-tailored content, and messages sent to match preferred dose time. The objective of the present study is to describe efforts toward exploring and preparing for implementation of iTAB at a large community-based organization serving sexual and gender minorities.

**Methods:**

We conducted 20 semi-structured key-informant interviews with clients of the Los Angeles LGBT Center who have experience taking PrEP. Participants self-identified as sexual (men who have sex with men) and/or gender (transgender and gender nonconforming) minorities. We used a rapid qualitative analytic approach to analyze interview data, while also turning to the Exploration, Preparation, Implementation, Sustainment (EPIS) framework to identify implementation determinants and innovation factors related to iTAB. Three team members applied a summary template with key domains drawn from EPIS and the interview guide. Key points from the summary templates were placed into a matrix to assess the breadth of information for each domain.

**Findings:**

Clients expressed highly favorable perceptions of the acceptability and fit of iTAB, with many identifying the customization, affirming/positive content, variety, brevity, and interactivity of text messages as appealing components. Clients also recommended several adaptations to iTAB to increase its fit: 1) provide access to a calendar documenting previous responses to iTAB adherence prompts; 2) incorporate messages to encourage prescription refills; 3) integrate options for self- and provider-assisted setup of iTAB; and 4) enable the provision or withholding of consent to share iTAB data with PrEP providers.

**Implications for D&I Research:**

The findings demonstrate the value of eliciting client feedback prior to intervention implementation as clients identified additional intervention adaptations not previously considered. This community-engaged research establishes a model for how EHE efforts can productively leverage implementation science models and methods to prepare for the strategic implementation of evidence-based interventions to support PrEP adherence among individuals at risk for HIV.

**Primary Funding Source:** National Institutes of Health

### S108 Seeking equity-centered implementation through co-production: Analysis of an IPO implementation support intervention

#### Megan Stanton^1^, Samira Ali^2^

##### ^1^Eastern Connecticut State University, Willimantic, CT, USA; ^2^University of Houston, Houston, TX, USA

###### **Correspondence:** Megan Stanton (stantonmeg@easternct.edu)

**Background:**

Multisectoral approaches to implementation require individual and institutional-level knowledge about diverse stakeholder engagement. Further, for approaches to meaningfully address health inequity, organizations must involve client community stakeholders as equal partners in intersectoral negotiation. Co-production refers to shared decision making between organizations and client communities around policy, programs, and implementation. Little is known about organizational capacity building strategies to facilitate co-production. The aims of this study are to 1) present an implementation support intervention (ISI) developed by an Intermediary/ Purveyor Organizations (IPO) which uses the ‘EPIS’ Implementation framework to facilitate co-production in HIV service change projects and 2) explore the impact of the ISI on implementation outcomes and organizational structure.

**Methods:**

Data were collected by the IPO evaluation team and consisted of: 1) IPO intervention protocols and field notes 2) in-depth interviews with organizational leadership (N=20) and 3) pre/post organizational assessments (N= 24). Organizational assessments were analyzed for over-time change. Field notes and interviews were analyzed using thematic analysis.

**Findings:**

The ISI identified co-production goals at each EPIS phase. Implementation support strategies mapped to these goals. For example, preparation phase goals included developing project decision-making structures that center the client community; hiring community members for the project team; and training staff to value co-production. ISI strategies to facilitate this included training on co-production and coaching (ex: guided implementation plan development, budget analysis and feedback). Organizational assessments demonstrated that after a year of receiving the ISI 100% of organizations increased their use of co-production strategies (10 items, mean=4.3, range 1-10) and 83% created sustainable co-production mechanisms. Interviews revealed how co-production influenced project implementation outcomes (acceptability, appropriateness, feasibility and adoption) in ways that better met the needs of community stakeholders.

**Implications for D&I Research:**

Analysis of the ISI demonstrates how an IPO can be a critical bridging factor to support integration of co-production into implementation, thus promoting equitable implementation outcomes, building sustainable organizational structures for power sharing and helping bridge the divide between institutions and client communities. Though utilized in participatory governance in the public sector, co-production has been under-considered as a strategy to advance equity-centered implementation.

**Primary Funding Source:** The Gilead COMPASS Initiative

### S109 When adaptation creates an unsanctioned environment: Understanding implementation and effectiveness of the first supervised consumption site in the United States

#### Barrot Lambdin^1^, Peter Davidson^2^, Lynn Wenger^3^, Erica Browne^3^, Alex Kral^1^

##### ^1^RTI International, Berkeley, CA, USA; ^2^San Diego, CA, USA; ^3^RTI International, San Francisco, CA, USA

###### **Correspondence:** Barrot Lambdin (blambdin@rti.org)

**Background:** The United States (US) has faced an unprecedented epidemic of drug overdose deaths for decades. Similarly, medical complications from injection drug use have increased. In 2014, an organization in an undisclosed location in the United States adapted the delivery of evidence-based interventions for people who inject drugs (PWID) and implemented an unsanctioned safe consumption site (SCS). The SCS ensured people had access to clean needles and naloxone during drug injection events, which were monitored by trained staff. We assessed the effectiveness and implementation of the unsanctioned SCS.

**Methods:** We describe adaptations made to the delivery of evidence-based interventions using the Model for Adaptation Design and Impact. We sought to understand implementation of the SCS using in-depth interviews with staff (n=11), community stakeholders (n=13) and participants (n=10) and analyzed the data using an inductive thematic approach. We evaluated the effectiveness of the SCS using service utilization data from the SCS, an interrupted time series analysis of police incident reports and a prospective cohort study using inverse probability of treatment weighting to account for time-varying confounding

**Findings:** Community members and participants adapted how evidence-based interventions were delivered to PWID such that the services operated in an unsanctioned environment. Themes from qualitative data included broad community support for SCS to improve health, concern that SCS might increase crime in the community, and fears of potential arrest by PWID and staff. All overdoses that occurred at the SCS were effectively reversed. The community surrounding the SCS experienced a statistically significant decline in criminal activity following SCS implementation, compared to two control communities (p<0.001). Of the 494 participants enrolled in the cohort study, 59 (12%) used the SCS at least once. People using SCS were 27% (95%CI: 12%–46%) less likely to visit the emergency department and 32% (95%CI: 4%–57%) less likely to be hospitalized than those who did not use the SCS.

**Implications for D&I Research:** A community engaged process led to delivery adaptations that were not authorized at the local, state or federal level. The unsanctioned environment led to implementation challenges, but despite these, the adaptations were effective at preventing drug use-related harms.

**Primary Funding Source:** Arnold Ventures

### S110 Implementation and outcomes of a system-wide women’s health “team goal” to reduce maternal morbidity for black women

#### Rebecca Hamm^1,2^, Elizabeth Howell^2^, Abike James^2^, Robert Faizon^3^, Tina Bloemer^4^, Jennifer Cohen^5^, Sindhu Srinivas^1,2^

##### ^1^University of Pennsylvania Leonard Davis Institute of Health Economics, Philadelphia, PA, USA; ^2^University of Pennsylvania Perelman School of Medicine, Philadelphia, PA, USA; ^3^Penn Medicine Lancaster General Health, Lancaster, PA, USA; ^4^Penn Medicine Princeton Medical Center, Princeton, NJ, USA; ^5^Chester County Hospital, West Chester, PA, USA

###### **Correspondence:** Rebecca Hamm (rebecca.feldmanhamm@pennmedicine.upenn.edu)

**Background:** In response to the unacceptable racial disparities in US obstetric outcomes, our health system established a bold, formal goal to reduce maternal morbidity for Black women. Here, we describe our process for meeting this equity-focused goal in the context of diverse implementation climates at 5 inpatient obstetric sites.

**Methods:** To meet the system goal, we established a collaborative of multidisciplinary, site-based teams. The validated 18-question Implementation Climate Scale (ICS) was distributed to all site clinicians at baseline. Sites were asked to focus on hemorrhage, performing case reviews of Black women meeting morbidity criteria using a standardized case report form. Comparing cases by site, site-specific areas for improvement in hemorrhage risk assessment, prevention, and management emerged. Evidence-based practices (EBPs) were then selected, tailored, and implemented by site. Monthly system-wide team meetings included (1) metric tracking, and (2) site presentations with discussions around barriers/facilitators to EBP implementation. Maternal morbidity rates (Vizient 4/5/6+CDC criteria) among Black women were compared the year before goal development (7/1/2019-6/30/2020) to the year after (7/1/2020-6/30/2021).

**Findings:** Mean ICS scores for inpatient obstetric units differed by site (p=0.005), with climates more supportive of implementation at urban/academic hospitals (Table). In response to case reviews, sites reported implementing 2 to 8 EBPs to meet the team goal, such as coding reviews, implicit bias training, and standardized treatment of prenatal anemia. Despite different ICS scores, this process was associated with significant reductions in maternal morbidity for Black women from pre- to post-goal development overall and Sites 1, 2, and 3, with non-statistically but likely clinically significant reductions at Sites 4 and 5 (Overall: -29.4% reduction, p<0.001).

**Implications for D&I Research:** A health system goal of reducing maternal morbidity for Black women led to a data driven, collaborative model for implementing site-tailored interventions. If health systems prioritize equity-focused goals, sites with an array of implementation climates can be supported in implementing EBPs that improve care for BIPOC populations.

**Table 1 (abstract S110).** Site outcomes

Site	ICS Score[0-4]	N	% Reduction in Black Maternal Morbidity Pre-Post	p-value
1	2.19(+/-0.52)	5698	-29.3%	<0.001
2	1.93(+/-0.55)	3326	-26.9%	<0.001
3	1.85(+/-0.62)	679	-44.6%	0.04
4	1.42(+/-0.78)	525	-48.1%	0.16
5	No responses	422	-23.8%	0.47

### S111 Evaluation of a statewide tobacco learning collaborative to support tobacco treatment integration in safety net health systems

#### Elisa Tong^1^, Tasleem Chechi^2^, Shannon Haggitt^3^, Moreen Sharma^4^, Cindy Valencia^3^, Ulfat Shaikh^5^

##### ^1^Internal Medicine, University of California, Davis, Sacramento, CA, USA; ^2^UC Davis, Sacramento, CA, USA; ^3^UC Davis, sacramento, CA, USA; ^4^University of California, Davis, Sacramento, CA, USA; ^5^University of California Davis, Sacramento, CA, USA

###### **Correspondence:** Elisa Tong (ektong@ucdavis.edu)

**Background:** Tobacco treatment is an important measure of quality care reported by safety net health systems. To support systems change, CA Quits is a statewide project funded by the California Department of Public Health. In partnership with California’s Medicaid program, CA Quits hosts a statewide Tobacco-cessation Learning Collaborative (TLC) on evidence-based tobacco treatment practices. This study evaluates the characteristics, engagement, and outcomes with participating systems.

**Methods:** The TLC framework was based on the Institute for Healthcare Improvement Breakthrough Series model which focuses on shared learning and improvement. The TLC had two cohorts: Public Hospital Clinic Systems, which consist of large Designated Public Hospitals (DPH) and smaller District Municipal Public Hospitals (DMPH), and Community Health Centers (CHC). Recruitment for DPH/DMPH were through the state Medicaid quality improvement incentive program and for CHC were through public health partners funded for tobacco control. Participants engaged in monthly learning sessions and interim action sessions for a 10-month duration in 2019 and/or 2020. Systems were assessed for workflow standardization, referrals to tobacco treatment, and tobacco assessment and treatment quality metrics (National Quality Forum metric 0028).

**Findings:** A total of 36 safety-net health systems participated in the TLC (7 DPH, 14 DMPH, 15 CHC). All DPH were urban while 64% DMPH and 40% CHC had rural designations. Nearly two-thirds fully participated in either one (4 DMPH, 10 CHC) or two years (2 DPH, 5 DMPH, 2 CHC). TLC participants improved standardization of clinical workflows for tobacco treatment by 124%. About two-thirds established electronic referrals for tobacco cessation (57% DMPH, 71% DPH, 73% CHC). For median values of quality metric improvement before and after the TLC, DMPH had the greatest increase (n=4, 57.5% to 97.9%), DPH had plateaued (n=7, 92.7% to 92.6%) and CHC increased slightly (n=15, 85.2% to 88.7%).

**Implications for D&I Research:** Collaborative learning environments are a promising strategy for public health partners to support tobacco treatment in safety net health systems. While a third of systems did not fully participate, nearly a quarter elected to participate for a second year. DMPH, which were predominantly rural, had the greatest improvement in tobacco quality metrics among participating systems.

**Primary Funding Source:** State tobacco control program

### S112 Advancing health equity through state, managed care, and health care inter-organizational Medicaid data alignment

#### Lauren Peterson^1^, Sivan Spitzer-Shohat^2^, Shilpa Patel^3^, Jaclyn Martin^1^, Marshall Chin^1^, Anne Smithey^3^, Nadia Glenn^4^

##### ^1^University of Chicago, Chicago, IL, USA; ^2^Bar-Ilan University, Safed, Israel; ^3^Center for Health Care Strategies, Hamilton, NJ, USA; ^4^Institute for Medicaid Innovation, Washington, DC, USA

###### **Correspondence:** Lauren Peterson (lapeterson@uchicago.edu)

**Background:** The Advancing Health Equity initiative established a Learning Collaborative composed of seven teams of state Medicaid agencies, managed care organizations (MCOs), and healthcare organizations (HCOs). Each team designs and implements a care delivery transformation and supportive payment innovation with a goal of reducing a health care disparity. The aim of this study is to identify barriers and facilitators of inter- and intra-organizational data collection, utilization, sharing, and alignment throughout the process.

**Methods:** We conducted semi-structured interviews with 49 Learning Collaborative stakeholders from state Medicaid agencies, MCOs, and HCOs across seven states. Analysis was guided by a theoretical conceptual model based on the Consolidated Framework for Implementation Research and May’s Theory of Implementation. Coding of interview transcripts included content and thematic analysis.

**Findings:** Some teams analyzed existing data to identify a disparity, while others first selected a disparity that guided their use of data. Regardless of approach, data resources, or state context, stakeholders expressed challenges with data availability, accuracy, and completeness in addition to limited data alignment across organizations and sectors. Focusing on one disparity and designing a data-informed, integrated care delivery and payment intervention to address the disparity as a multi-stakeholder team served as a catalyst to engage stakeholders in data discussions, which helped to identify data limitations and complementary data resources, and to improve inter-organizational data collection, sharing, and alignment, particularly for race, ethnicity, and language data. One state Medicaid stakeholder observed, “They have come to a better understanding of the data that they each have and where it comes from and what its limitations might be and how to share it... I do think that sorting out data discrepancies was more time-consuming than anyone expected it to be... the information that each side had was just further apart than I think anyone expected.”

**Implications for D&I Research:** Coalescing government actors, payers, and health care providers around a disparity can facilitate inter-organizational data sharing, integration, and alignment. Using inter-organizational data to examine a disparity in multiple contexts, including care delivery and payment, enables stakeholders to better understand limitations of existing data, identify complementary data resources, and specify needs for future data collection and integration.

**Primary Funding Source:** The Robert Wood Johnson Foundation

### S113 Evaluating inpact intervention-context fit in low-resource school districts in central Michigan

#### Amy Wassmann^1^, Michele Marenus^2^, Andria Eisman^3^, Thomas Templin^2^, Lynn Malinoff^4^, Ronald Zernicke^2,5^, Lexie Beemer^6^, Anna Schwartz^2^, Tiwaloluwa Ajibewa^2^, Rebecca Hasson^2,5^

##### ^1^Saginaw Intermediate School District, Saginaw, MI, USA; ^2^University of Michigan School of Kinesiology, Ann Arbor, MI, USA; ^3^Wayne State University School of Education, Detroit, MI, USA; ^4^Eastern Michigan University, Ypsilanti, MI, USA; ^5^University of Michigan Exercise & Sport Science Initiative, Ann Arbor, MI, USA; ^6^University of Michigan, School of Kinesiology, Ann Arbor, MI, USA

###### **Correspondence:** Amy Wassmann (wassmanna@sisd.cc)

**Background:** Classroom-based physical activity (PA) interventions are often adopted immediately without any consideration of the organizational capacity and system-level policies in place that influence implementation. The purpose of this study was to conduct pre-implementation assessments of the intervention-context fit of the Interrupting Prolonged sitting with ACTivity (InPACT) intervention in one low-resource, low-active intermediate school district (ISD; 32 schools and 16 districts) in central Michigan.

**Methods:** Assessments were conducted by the Regional School Health Coordinator along with other ISD support staff during the 2019-2020 and 2020-2021 academic years. The Hexagon Discussion and Analysis Tool was used to assess need, fit, support, evidence, usability, and capacity related to InPACT program implementation. The assessment team rated each indicator on a 5-point Likert scale. Total scores were calculated by summing the indicator scores; higher scores indicated strong intervention-context fit. Qualitative notes from the meetings were recorded and analyzed with quantitative scores.

**Findings:** Total score for the first assessment was 19 (out of 30), and 28 for the second assessment, resulting in a 47% increase in intervention-context fit over a one year period. Qualitative data revealed that improvements in need, fit, and capacity were related to increased awareness of the need to provide more PA opportunities for students in school during the COVID-19 pandemic. Improvements in support, evidence, and usability were related to increased engagement of the Regional School Health Coordinator with program developers and increased knowledge of the InPACT program.

**Implications for D&I Research:** Conducting an intervention-context fit assessment during the pandemic informed how a central Michigan ISD moved from exploration to preparation to increase district-wide intervention effectiveness and sustainment. The Hexagon Tool is a pragmatic resource that is useful for school administrators to evaluate organizational capacity and system-level policies in place when seeking to adopt and implement new PA interventions in school settings.

**Primary Funding Source:** Michigan Health Endowment Fund

